# Tandem Allylboration–Prins Reaction for the Rapid Construction of Substituted Tetrahydropyrans: Application to the Total Synthesis of (−)‐Clavosolide A

**DOI:** 10.1002/anie.201511140

**Published:** 2016-01-14

**Authors:** Alba Millán, James R. Smith, Jack L.‐Y. Chen, Varinder K. Aggarwal

**Affiliations:** ^1^ School of ChemistryUniversity of BristolCantock's CloseBristolBS8 1TSUK

**Keywords:** allylboration, lithiation–borylation, natural products, Prins reaction, total synthesis

## Abstract

Tetrahydropyrans are common motifs in natural products and have now been constructed with high stereocontrol through a three‐component allylboration‐Prins reaction sequence. This methodology has been applied to a concise (13 steps) and efficient (14 % overall yield) synthesis of the macrolide (−)‐clavosolide A. The synthesis also features an early stage glycosidation reaction to introduce the xylose moiety and a lithiation‐borylation reaction to attach the cyclopropyl‐containing side chain.

Creating increasingly efficient syntheses of common structural motifs found in Nature is a long‐running objective in organic synthesis. For example, substituted pyrans are frequently encountered in the family of polyketide natural products.^1^ Clavosolide A^2^, ^3^ is a contemporary example, whose correct structure was established following total syntheses by Willis,3a Lee3b and Smith3c (Figure 1). This molecule has been prepared by a variety of strategies and the tetrasubstituted tetrahydropyran (THP) core alone has been constructed in ≥6 steps.^3^ In some cases additional steps were employed to construct THPs with high structural complexity towards the end of the synthesis.

**Figure 1:**
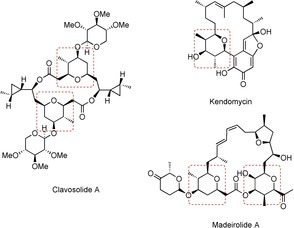
Examples of THP‐containing natural products.

Lithiation–borylation^4^ has emerged as a powerful tool for the synthesis of chiral boronic esters including allylic boronic esters.^5^ We reasoned that this methodology, in combination with our improved allylboration of aldehydes^6^ followed directly by a Prins cyclisation^7^, ^8^ could lead to a short, and highly stereoselective synthesis of the THP core of (−)‐clavosolide A in just three steps. In this paper we report our success in not only developing the three‐component allylboration–Prins reaction for the rapid stereocontrolled assembly of substituted THPs but also in developing further improvements to our lithiation–borylation protocol and addressing other issues of stereocontrol so that every step in our short synthesis is highly stereoselective (>95:5 dr). Furthermore, and as previously described in earlier syntheses of (−)‐clavosolide A3f,3i,3j and B,^9^ conducting the glycosidation step early in the synthesis rather than at the end avoids the formation of statistical mixtures of anomeric stereoisomers (α,α; α,β; β,β), thereby improving the overall yield.

Our retrosynthetic analysis is shown in Scheme 1 and involves the initial disconnection of the macrocyclic lactone to the hydroxy acid **2**. We then envisaged incorporation of the cyclopropyl unit through a lithiation–borylation reaction between carbamate **3** and boronic ester **4**. Whilst substrate‐controlled cyclopropanation of allylic alcohols is well established^10^ and has previously been employed in the synthesis of (−)‐clavosolide A,3c,3e–3g unfortunately it gives the undesired diastereoisomer and therefore requires additional steps for correction. The carbamate bearing the THP core **3** could potentially be assembled by a three‐component allylboration–Prins reaction from boronic ester (*R*)‐**6 a** and aldehyde **7 a**.

**Scheme 1:**
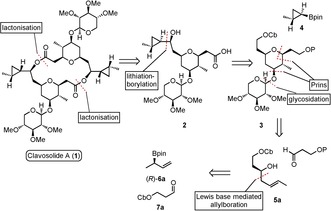
Retrosynthetic analysis for the synthesis of (−)‐clavosolide A. OCb=*N*,*N*‐diisopropyl carbamate; pin=pinacol.

The allylboration–Prins reaction was initially investigated using our improved Lewis base mediated allylboration reaction (with *n*BuLi and TFAA additives).

As shown in Table 1, a control experiment was conducted involving the allylboration (without the use of additives) of CyCHO with allylic boronic ester **6 a,** followed directly by a TFA‐mediated Prins reaction with a second portion of the same aldehyde. The reaction occurred in high yield but with low diastereoselectivity (35:65 dr; entry 1). The diastereoselectivity was reversed and substantially improved when applying our recently developed Lewis base mediated allylboration reaction conditions (with *n*BuLi and TFAA additives) to this process (87:13 dr; entry 2). This three‐component allylboration–Prins reaction enabled boronic esters (**6 a**/**6 b**) to be sequentially reacted with two different aldehydes to give THPs **8 b**–**g** in good to high yields and good diastereoselectivities (entries 3–8). We have previously employed neopentyl boronic esters in related reactions, but their instability towards silica gel purification necessitated their use in crude form, which resulted in considerably lower yields.^8^ The current protocol, using the more stable pinacol boronic esters, is more practical and leads to higher yields.

**Table 1:** Three‐component allylboration/Prins cyclization. 
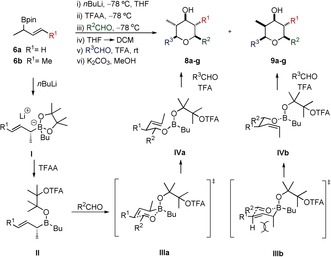

Entry^[a]^	R^1^	R^2^	R^3^	**8**	Yield [%]^[b]^	dr (**8:9**)
1^[c]^	H	Cy	Cy	**8 a**	91	35:65
2	H	Cy	Cy	**8 a**	84	87:13
3	H	Cy	Ph(CH_2_)_2_	**8 b**	75	87:13
4	H	Ph(CH_2_)_2_	Cy	**8 c**	57	89:11
5	H	Ph(CH_2_)_2_	Ph(CH_2_)_2_	**8 d**	65	88:12
6	Me	Ph(CH_2_)_2_	Ph(CH_2_)_2_	**8 e**	89	90:10
7	Me	Ph(CH_2_)_2_	Cy	**8 f**	81	91:9
8	Me	Cy	Cy	**8 g**	86	88:12

[a] General procedure: i) **6 a**–**b** (1.0 equiv), *n*BuLi (1.1 equiv), THF, −78 °C, 15 min; ii) TFAA (1.2 equiv), −78 °C, 30 min; iii) R^2^CHO (1.05 equiv), −78 °C, 2 h then RT, 16 h; iv) solvent exchange to DCM; v) R^3^CHO (3 equiv), TFA:DCM (1:3), RT, 2 h; vi) K_2_CO_3_ (1.5 equiv), MeOH, RT, 15 min. [b] Combined isolated yields of **8** and **9**. [c] Control experiment: i) **6 a** (1 equiv), CyCHO (1.05 equiv), THF, RT, 16 h; iv) solvent exchange to DCM; v) CyCHO (3 equiv), TFA:DCM (1:3), RT, 2 h; vi) K_2_CO_3_ (1.5 equiv), MeOH, RT, 30 min; TFAA=trifluoroacetic anhydride; TFA=trifluoroacetic acid; THF=tetrahydrofuran; DCM=dichloromethane.

The general protocol involved initial treatment of the allylic boronic ester with *n*BuLi and TFAA to give intermediate borinic ester **II**, which was reacted with the first aldehyde to give intermediate **IV**. Without isolation, and following solvent exchange to DCM, subsequent reaction with a second aldehyde in the presence of TFA followed by base mediated hydrolysis furnished the hydroxy THPs **8 a**–**g**. The modified allylboration reaction occurs via the more reactive borinic ester **II**, which reacts with the aldehyde through a Zimmerman–Traxler chair transition state (TS) **III**. The reduced steric hindrance around boron in the borinic ester when compared to the pinacol ester results in greater preference for the reaction to occur via TS **IIIa**, with the methyl group situated in a pseudo‐equatorial position, leading to the higher observed diastereoselectivity. The diastereoselectivity of THP **8 a** directly reflects the *E*/*Z* selectivity obtained in the initial allylboration of the aldehyde.^6^

In order to apply this methodology to the synthesis of (−)‐clavosolide A, we required the reaction of allylic boronic ester (*R*)‐**6 a** with aldehyde **7 a**, followed by aldehyde **10** (Scheme 2). Boronic ester (*R*)‐**6 a** was obtained in two steps from ethanol using our lithiation–borylation methodology with (−)‐sparteine, in high yield and high er. However, the three‐component allylboration–Prins reaction gave THP **11** in low yield (due to concomitant cleavage of the silyl protecting group) but good diastereoselectivity (88:12 dr). In search for an alternative group to a silyl ether (TIPS and TBDPS silyl ethers were also labile under the reaction conditions), we considered the use of the simplest unsaturated aldehyde, acrolein.^11^ We found that the three component allylboration‐Prins reaction worked well when using acrolein, furnishing the THP **12 a** in high yield and good diastereoselectivity (88:12 dr). Taking advantage of the considerably higher reactivity of the borinic ester intermediate, we were able to improve the diastereoselectivity (96:4 dr) by simply reducing the temperature of the reaction to −100 °C. Thus, with this straightforward protocol we were able to convert the simple reagents (*R*)‐**6 a**, **7 a** and acrolein into the complex THP **12 a** in high yield and with high stereocontrol.

**Scheme 2:**
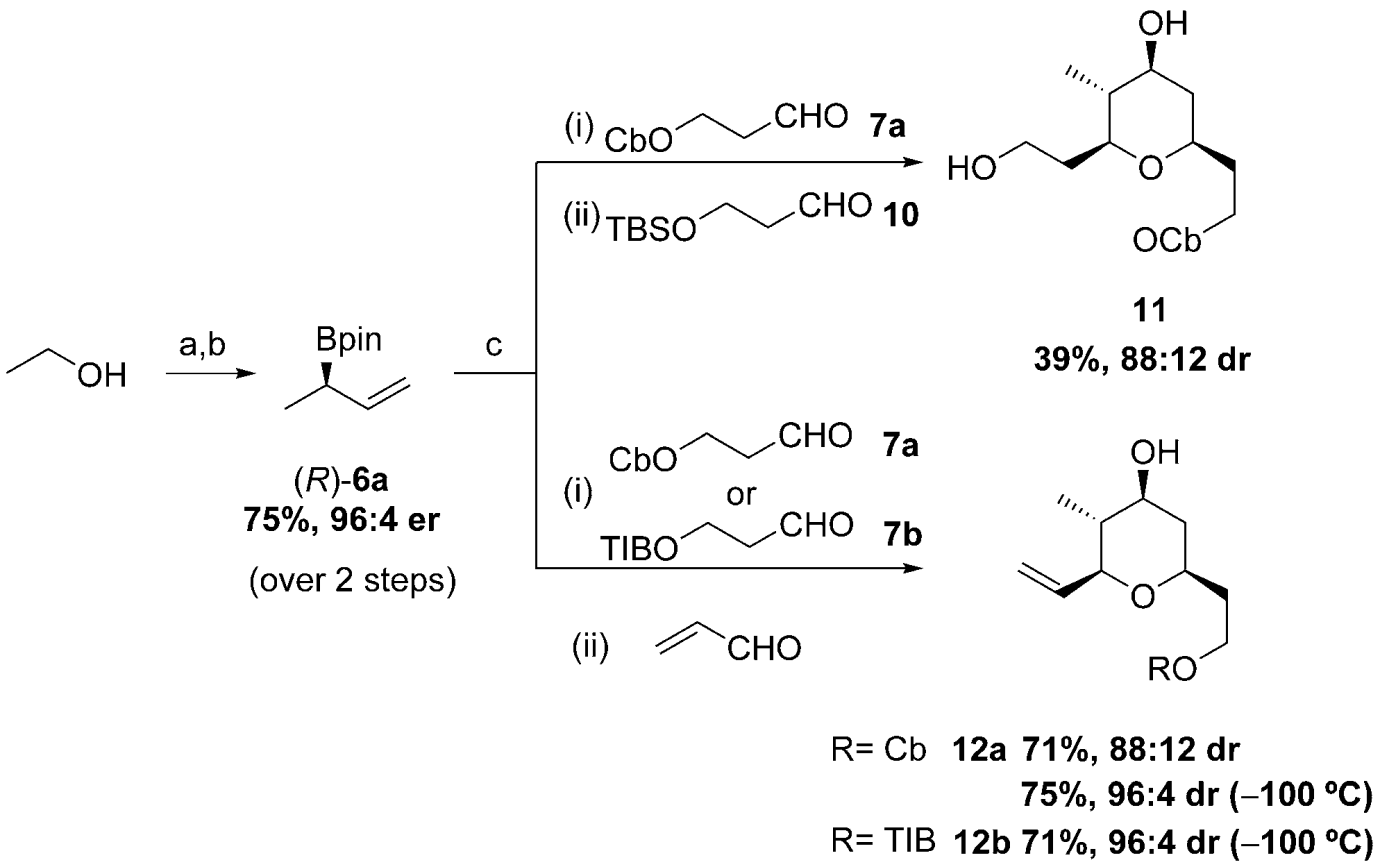
Synthesis of the THP core. a) Cb‐Cl (1 equiv), Et_3_N (1.2 equiv), 16 h, reflux; b) i) *s*BuLi (1.1 equiv), (−)‐sp (1.2 equiv), Et_2_O, −78 °C, 5 h; ii) vinyl boronic acid pinacol ester (1.1 equiv), −78 °C, 1 h; iii) MgBr_2_⋅OEt_2_ (2 equiv), −78 °C, 10 min, then reflux, 16 h; c) i) *n*BuLi (1.1 equiv), THF, −78 °C, 15 min; ii) TFAA (1.2 equiv), −78 °C, 30 min; iii) **7 a** or **7 b** (1.5 equiv), −78 °C (or −100 °C), 2 h then RT, 16 h; iv) solvent exchange to DCM; v) **10** (2.5 equiv,) or acrolein (4 equiv), TFA:DCM (1:3), RT, 2 h; vi) K_2_CO_3_ (1.5 equiv), MeOH, RT, 30 min; TBS=*tert‐*butyldimethylsilyl; OTIB=2,4,6‐triisopropylbenzoate; sp=sparteine.

Having developed a short three‐step route towards the THP core, we considered the glycosidation next. Since the xylose moiety was ultimately required in the target molecule, we believed that it could also serve as a protecting group, thereby minimising the number of additional steps. Unfortunately, using the permethylated glycosyl donor analogous to **13** either a 1:1 mixture of diastereoisomers (α,β) or no reaction was observed under a variety of reaction conditions.^12^ We therefore turned to exploiting neighbouring group participation to control the desired β‐selectivity.^13^ Both the perbenzoate **13** 14 and corresponding peracetate^15^ were tested, but the latter suffered from competing acetylation of the hydroxy group in the pyran ring.^16^ Thus, reaction of the trichloroacetimidate **13** with pyran **12 a** in the presence of TMSOTf gave the corresponding adduct in high yield and with perfect stereocontrol. Subsequent hydrolysis of the benzoate, followed by permethylation gave glycoside **3 a** in 88 % yield over the three steps. Finally, hydroboration, oxidation and protection gave the silyl ether **14 a**, setting the stage for the final lithiation–borylation reaction to introduce the cyclopropyl moiety.^17^

The final C−C bond construction required a late‐stage^18^ lithiation–borylation reaction and this step proved to be quite challenging. Lithiation of the highly oxygenated carbamate **14 a** under our standard conditions [*s*BuLi (1.1 equiv), (+)‐sparteine (1.2 equiv), in Et_2_O at −78 °C, for 5 h], followed by borylation with the known boronic ester **4** (96:4 er)^19^ and subsequent oxidation gave the desired alcohol **15 a** in 23–48 % yield and >95:5 dr, together with recovered starting material (≈40 %). Longer reaction times or increased amounts of base did not improve the yield and led to less recovered starting material. Analysis of the crude reaction mixture showed that competing deprotonation was occurring on the glycoside ring,^20^ perhaps because of competing complexation of the organolithium with the highly oxygenated moiety. We therefore turned to the tri‐isopropylbenzoyl (TIB) ester in place of the carbamate. Although this group has been used previously to promote 1,2‐migration in difficult lithiation–borylation reactions involving poor migrating groups,^21^ we reasoned that its greater electron withdrawing capacity (which made it a better leaving group) might also increase the acidity of the α‐protons, promoting lithiation.^22^ We therefore brought the TIB ester **12 b** through the same sequence of steps to the carbamate. This time, lithiation–borylation of the TIB ester **14 b** gave the desired alcohol **15 a** in 73 % yield and >95:5 dr (Scheme 3).

**Scheme 3:**
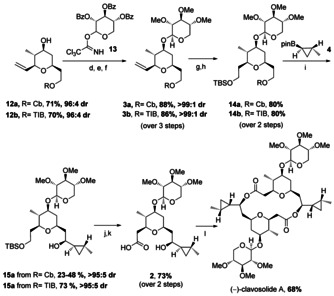
Synthesis of (−)‐clavosolide A. d) **13** (1.5 equiv), TMSOTf (0.3 equiv), 4 Å MS, DCM, −20 °C to RT, 3 h; e) NaOMe (3.6 equiv), MeOH, RT, 1 h; f) NaH (8 equiv), MeI (8 equiv), DMF, RT, 16 h; g) i) (cHex)_2_BH (6 equiv), THF, 0 °C, 4 h; ii) H_2_O_2_, NaOH, 0 °C to 55 °C, 2 h; h) TBSCl (1.2 equiv), Et_3_N (1.2 equiv), DCM, RT, 16 h; i) when R=Cb: i) *s*BuLi (1.1 equiv), (+)‐sp (1.2 equiv), Et_2_O, −78 °C, 5 h; ii) **4** (1.2 equiv), −78 °C, 1 h, then reflux, 16 h; iii) NaOH (2 m): H_2_O_2_ (30 %) (2:1), RT, 2 h; when R=TIB: i) *s*BuLi (1.1 equiv), (+)‐sp (1.2 equiv), Et_2_O, −78 °C, 1 h; ii) **4** (1.2 equiv), −78 °C, 1 h, then reflux, 2 h; iii) NaOH (2 m): H_2_O_2_ (30 %) (2:1), RT, 2 h; j) 1 % HCl, EtOH, 20 min, 80 %; k) TEMPO (0.01 equiv), KBr (0.1 equiv), NaHCO_3_, NaOCl, H_2_O, DCM, 0 °C, 5 min, 87 %; l) i) 2,4,6‐trichlorobenzoyl chloride (1.1 equiv), Et_3_N (1.3 equiv), THF, RT, 2.5 h; ii) DMAP (5 equiv), toluene, reflux, 16 h. OBz=benzoate; TEMPO=2,2,6,6‐tetramethylpiperidine 1‐oxyl; DMAP=4‐(dimethylamino) pyridine.

In order to demonstrate the versatility of this methodology towards making alternative stereoisomers without modifying the route, further homologations of TIB ester **14 b** were conducted. As shown in Scheme 4, using either of the two chiral diamines (+)‐sparteine/(−)‐sparteine (L) with either of the two enantiomeric boronic esters **4** (96:4 er)/*ent*‐**4** (99:1 er), enabled us to prepare each of the four diastereoisomers **15 a**–**d** selectively and in good yield. A small matched/mis‐matched effect was observed in the lithiation step, presumably as a result of competing complexation with the internal pyran oxygen, which led to lower diastereoselectivity in the cases of **15 b**/**15 d**.^23^ Interestingly, both matched and mis‐matched cases were equally efficient. Furthermore, this stereodivergent synthesis, enabling other diastereomers to be accessed very simply without changing the route,^24^ is especially relevant and important for structures like clavosolide A, whose stereochemistry had initially been incorrectly assigned. Alcohol **15 c** would have led to the originally proposed structure of clavosolide A, whilst **15 a** leads to the synthesis with the correct structure.

**Scheme 4:**
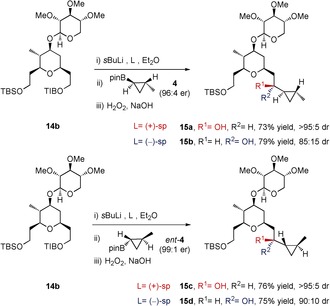
Synthesis of alternative diastereoisomers of alcohol **15 a** using the lithiation–borylation reaction. Reaction conditions: i) *s*BuLi (1.1 equiv), L (1.2 equiv), Et_2_O, −78 °C, 1 h; ii) Boronic ester **4** or *ent*‐**4** (1.2 equiv), −78 °C, 1 h, then reflux, 2 h; iii) NaOH (2 m): H_2_O_2_ (30 %) (2:1), RT, 2 h.

The completion of the synthesis involved acid‐catalysed deprotection of the silyl group, selective oxidation of the primary alcohol to the carboxylic acid^25^ **2** and dimerization under Yamaguchi's conditions.^26^ This gave synthetic (−)‐clavosolide A in good yield, whose ^1^H and ^13^C NMR spectra were identical to the natural product.^2^

In conclusion, we have shown that commonly occurring substituted tetrahydropyrans can be assembled in just 3 steps with high stereocontrol using a three‐component allylboration–Prins reaction. This has been applied to a concise and efficient synthesis of (−)‐clavosolide A in just 13 steps and 14 % overall yield, where all steps occurred with >95:5 selectivity. Additional noteworthy features include 1) an early stage diastereoselective glycosidation reaction to introduce the xylose moiety and 2) diastereoselective lithiation–borylation reaction of a highly oxygenated hindered TIB ester, which shows enhanced acidity over standard carbamates, enabling improved lithiations leading to significantly higher yields.

## Supporting information

As a service to our authors and readers, this journal provides supporting information supplied by the authors. Such materials are peer reviewed and may be re‐organized for online delivery, but are not copy‐edited or typeset. Technical support issues arising from supporting information (other than missing files) should be addressed to the authors.

Supplementary
