# Supplementary material for: Tandem Allylboration–Prins Reaction for the Rapid Construction of Substituted Tetrahydropyrans: Application to the Total Synthesis of (−)‐Clavosolide A
Source: Angew Chem Int Ed Engl. 2016 Jan 14;55(7):2498–502. doi: 10.1002/anie.201511140 (PMC4755224; doi:10.1002/anie.201511140)
Supplement: Supplementary file 1 — Supplementary [file ANIE-55-2498-s001.pdf]

Angewandte Chemie
Eine Zeitschrift der Gesellschaft Deutscher Chemiker

Supporting Information

Tandem Allylboration–Prins Reaction for the Rapid Construction of Substituted Tetrahydropyrans: Application to the Total Synthesis of (−)-Clavosolide A

*Alba Millán, James R. Smith, Jack L.-Y. Chen, and Varinder K. Aggarwal**

anie_201511140_sm_miscellaneous_information.pdf

Contents

1. General information……………………………………………………………………………… 3

2. Materials and reagents………………………………………………………………………… 4

3. Studies on the three-component allylboration-Prins reaction…………………… 5
   a.) Synthesis of allylic boronic ester 6a……………………………………………… 5

   b.) Synthesis of allylic boronic ester 6b……………………………………………… 7

   c.) General procedure for the synthesis of 4-hydroxy THPs 8a-g (Table 1)… 8

4. Total synthesis of of (−)-clavosolide A………………………………………………… 14

5. ^{1}H and ^{13}C NMR data of natural and synthetic clavosolide A …………………… 33

6. ^{1}H and ^{13}C NMR spectra……………………………………………………………… 34

7. HPLC and GC analysis………………………………………………………………………… 61

1. General information

All required fine chemicals were used directly without purification unless mentioned. All air- and water-sensitive reactions were carried out in flame-dried glassware under nitrogen atmosphere using standard Schlenk manifold technique.

^{1}H- and ^{13}C- Nuclear Magnetic Resonance (NMR) spectra were acquired at various field strengths as indicated, and were referenced to $CHCl_3$ (7.27 and 77.0 ppm for ^{1}H and ^{13}C respectively). ^{1}H NMR coupling constants are reported in Hertz and refer to apparent multiplicities and not true coupling constants. Data are reported as follows: chemical shift, multiplicity (s = singlet, br s = broad singlet, d = doublet, t = triplet, q = quartet, quin = quintet, m = multiplet, dd = doublet of doublet, etc.) and integration. ^{11}B-NMR spectra were recorded with complete proton decoupling using $BF_3 \cdot Et_2O$ (0.0 ppm) as an external standard.

High resolution mass spectra were recorded using Electron Spray Ionization (ESI). All IR data was obtained on a Perkin-Elmer Spectrum One FT-IR spectrometer. Optical rotations were obtained on a Perkin-Elmer 241MC polarimeter. Melting points were determined with a Boetius hot stage apparatus and were not corrected.

Analytical TLC were performed using aluminium backed plates pre-coated (0.25 mm) with Merck Silica Gel 60 F254. Compounds were visualized by exposure to UV-light or by dipping the plates in phosphomolybdic acid (PMA) or $KMnO_4$ followed by heating. Flash column chromatography was performed using Merck Silica Gel 60 (40-63 µm). All mixed solvent eluents are reported as v/v solutions.

Chiral HPLC was performed using a Diacel Chiralpak AS-H column (4.6 × 250 mm × 5 µm) and AD-H (4.6 × 250 mm × 5 µm) fitted with guards (4 × 10 mm), and monitored by DAD (Diode Array Detector). GC was performed on Agilent Technologies 6890N Network GC system using an Agilent Technologies HP-5 column (15 m × 2.5 mm x 0.25 µm), and monitored by FID (Flame Ionisation Detector).

2. Materials and reagents

All reagents were used as received unless otherwise stated. Anhydrous THF, CH_2Cl_2, toluene, hexane and Et_2O were dried by passing through a modified Grubbs system of alumina columns, manufactured by Anhydrous Engineering. Anhydrous Et_2O, THF and CH_2Cl_2 were stored over 3 Å molecular sieves. Petroleum ether refers to the fraction collected between 40-60 °C. TMEDA, DBU and Et_3N were distilled over CaH_2 and stored in a Young's tube under N_2. (−)-Sparteine was obtained from the commercially available sulfate pentahydrate salt (ABCR chemicals) and isolated according to literature procedure.[1] (+)-Sparteine was obtained as the free base (BOC sciences), distilled over CaH_2 or NaOH and stored in a Young's tube under N_2. The sparteine free base readily absorbs atmospheric carbon dioxide (CO_2) and should be stored in a Young's tube under inert atmosphere at −20 °C. Cyclohexanecarboxaldehyde, hydrocinnamaldehyde and benzaldehyde were distilled over molecular sieves using a Hickman distillation apparatus and stored in a Young's tube under N_2. NaH (60% oil dispersion) was washed three times with dry hexane under N_2 prior to use. Copper (I) chloride was purified by the addition of 37% aq. HCl followed by the addition of water and filtration and was dried under high vac overnight and kept under N_2. Organolithiums were periodically titrated using N-benzylbenzamide.[2]

Compounds **4**,[3] ***4-ent***,[3] **10**[4] and **18**[5] were prepared according to the literature and all spectroscopic data match with the reported ones.

---

[1] N. A. Nikolic, P. Beak, *Org. Synth.* **1997**, *74*, 23.
[2] A. F. Burchat, J. M. Chong, N. Nielsen, *J. Organomet. Chem.* **1997**, *542*, 281.
[3] H. Lin, W. Pei, H. Wang, K. N. Houk, I. J. Krauss, *J. Am. Chem. Soc.* **2013**, *135*, 82.
[4] X. Han, P. E. Floreancig, *Angew. Chem. Int. Ed.* **2014**, *53*, 11075.
[5] J. L. Stymiest, G. Dutheuil, A. Mahmood, V. K. Aggarwal, *Angew. Chem. Int. Ed.* **2007**, *46*, 7491.

3. Studies on the three-component allyboration- Prins reaction.

*a) Synthesis of allylic boronic ester **6a***

Racemic compound **6a** was synthesized following two different methodologies: a copper(I)-catalyzed allylic borylation reaction[6] (method A) and a lithiation-borylation reaction (method b).

- Method A

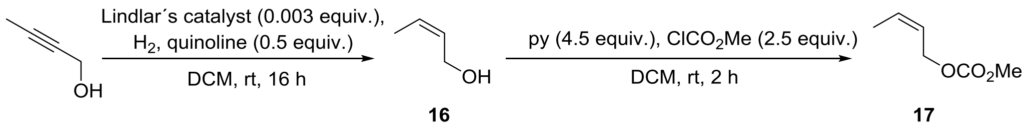

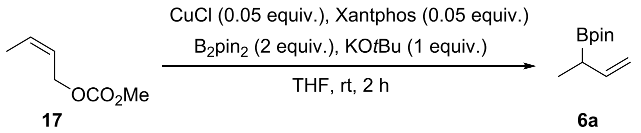

*Synthesis of (Z)-allylic carbonate **17**[7]*

A mixture of 2-butyn-1-ol (2.13 g, 30.4 mmol, 1 equiv.), quinoline (1.8 mL, 15 mmol, 0.5 equiv.) and Lindlar catalyst (5% w/w Pd mixed with $CaCO_3$ and doped with Pb, 223 mg, 0.003 equiv.) in dry DCM (30 mL) were stirred at rt under a H_2 atmosphere (balloon) overnight. The mixture was then filtered through a short silica pad eluting with dry DCM (70 mL). The crude solution was submitted to the next step without further purification.

To the 100 mL crude solution was added pyridine (10.9 mL, 135 mmol, 4.5 equiv.) at room temperature. Methyl chloroformate (5.8 mL, 75 mmol, 2.5 equiv.) was added at 0 °C and the reaction was stirred for 2 h at rt. Brine was added to the reaction mixture and the phases were separated. The aqueous phase was extracted with Et_2O (x3) and the combined organic layers were washed with $NaHCO_3$ sat. (x2) and water (x1), dried over $MgSO_4$, filtered and evaporated under reduced pressure. The residue was purified by flash column chromatography (SiO_2, pentane: Et_2O, 20:1). (*Z*)-allylic carbonate **17** was obtained as a colourless oil in 58% yield over 2 steps (2.28 g, 17.4 mmol).

^{1}H NMR (400 MHz, $CDCl_3$) δ (ppm) 5.81 – 5.69 (m, 1H), 5.61 – 5.52 (m, 1H), 4.69 (d, *J* = 6.9 Hz, 2H), 3.76 (s, 3H), 1.70 (d, *J* = 7.0 Hz, 3H).

^{13}C NMR (101 MHz, $CDCl_3$) δ (ppm) 155.9 (C), 130.4 (CH), 123.7 (CH), 63.5 (CH_2), 54.8 (CH_3), 13.2 (CH_3).

---

[6] H. Ito, T. Miya, M. Sawamura, *Tetrahedron* **2012**, 3423.

[7] H. Ito, S. Ito, Y. Sasaki, K. Matsuura, M. Sawamura, *J. Am. Chem. Soc.* **2007**, *129*, 14856.

The spectroscopic data are in accordance with the literature.[7]

*Copper(I)-catalyzed allylic borylation for the synthesis of allylic boronic ester **6a*** [6]

Into a flask, bis(pinacolato)diboron (2.54 g, 10.0 mmol, 2 equiv.), copper(I) chloride (25 mg, 0.25 mmol, 0.05 equiv.), and Xantphos (145 mg, 0.250 mmol, 0.05 equiv.) were placed. The flask was evacuated and filled with N_2. A 1 M THF solution of KO*t*Bu (5.0 mL, 5.0 mmol, 1 equiv.) and THF (5 mL) were added (*exothermic*). After stirring for 30 min, allylic carbonate **17** was added (651 mg, 5.00 mmol, 1 equiv.) and the reaction was stirred for 3 h at rt. The reaction mixture was passed through a short silica path with an eluent (pentane: Et_2O, 9:1) and the solvent was evaporated under reduced pressure at 0 °C. The crude reaction mixture was purified by flash column chromatography (SiO_2, pentane: Et_2O, 20:1) obtaining the corresponding boronic ester **6a** in 75% yield (682 mg, 3.75 mmol) as a colourless oil.

Note 1: To obtain the final compound in good yield, the chromatography should be completed within 10 min, since the allylboronic ester decomposes readily on silica gel.

^{1}H NMR (300 MHz, $CDCl_3$) δ (ppm) 5.93 (ddd, *J* = 17.4, 10.3, 7.1 Hz, 1H), 5.03 – 4.83 (m, 2H), 1.96 – 1.79 (m, 1H), 1.22 (m, 12H), 1.09 (d, *J* = 7.3 Hz, 3H).

^{13}C NMR (75 MHz, $CDCl_3$) δ (ppm) 141.0 (CH), 112.0 (CH_2), 83.3 (2xC), 24.75 (2xCH_3), 24.72 (2xCH_3), 22.4 (CH), 14.2 (CH_3).

^{11}B NMR (96 MHz, $CDCl_3$) δ (ppm) 33.

The spectroscopic data are in accordance with the literature.[7]

- Method B

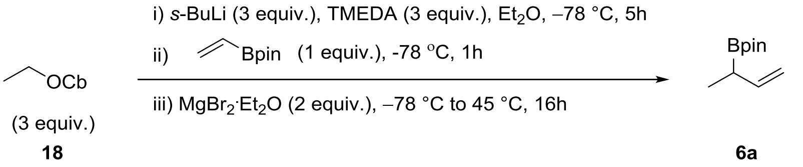

*Synthesis of allylic boronic ester **6a** via lithiation-borylation*

To a solution of primary carbamate **18** (3.00 g, 17.3 mmol, 3 equiv.) and TMEDA (2.59 mL, 17.3 mmol, 3.0 equiv.) in Et_2O (85 mL) at −78°C was added *s*-BuLi (13.3 mL, 1.30 M in hexanes, 17.3 mmol, 3.0 equiv.) dropwise. The resulting mixture was stirred for 5 h at −78 °C and then vinyl boronic acid pinacol ester (0.97 mL, 5.7 mmol, 1.0 equiv.) was added dropwise. The reaction mixture was further stirred at −78 °C for 1 h. $MgBr_2$•OEt_2 in Et_2O, made as follows, was added to the reaction mixture and stirred for 10 min: *1,2-Dibromoethane (0.98 mL, 11 mmol, 2 equiv.) was added to a suspension of magnesium (278 mg, 11.4 mmol, 2 equiv.) in Et_2O (14 mL) at room temperature. The reaction flask was then placed into a water bath in order to control the exothermic reaction and was stirred for 2 h.* The reaction was then warmed to room temperature and refluxed for 16 hours. The reaction mixture was allowed to cool down to room temperature,

filtered through a short silica pad (using pentane as eluent) and evaporated under reduced pressure at 0 °C. The crude reaction mixture contained a mixture of **6a**:vinyl boronic acid pinacol ester (9:1 by ^{1}H NMR). Purification was achieved by flash column chromatography (SiO_2, pentane: Et_2O, 97:3) to afford compound **6a** (as a single compound) as a colourless oil (312 mg, 30% yield), and a mixture of **6a**:vinyl boronic acid pinacol ester (290 mg, 81:19 ratio).
R_f = 0.35 (pentane: Et_2O, 19:1).
^{1}H NMR (300 MHz, $CDCl_3$) δ (ppm) 5.93 (ddd, *J* = 17.4, 10.3, 7.1 Hz, 1H), 5.03 – 4.83 (m, 2H), 1.96 – 1.79 (m, 1H), 1.22 (s, 12H), 1.09 (d, *J* = 7.3 Hz, 3H).
^{13}C NMR (75 MHz, $CDCl_3$) δ (ppm) 141.0 (CH), 112.0 (CH_2), 83.3 (2xC), 24.75 (2xCH_3), 24.72 (2xCH_3), 22.4 (CH), 14.2 (CH_3).
^{11}B NMR (96 MHz, $CDCl_3$) δ (ppm) 33.
Spectroscopic data are in accordance with the literature.[7]

b) Synthesis of allylic boronic ester **6b**

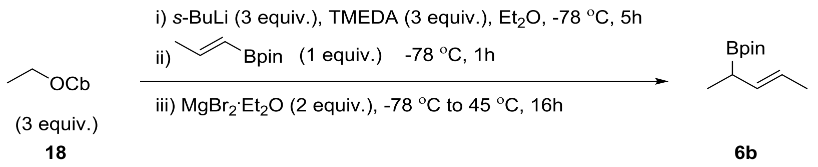

<u>*Synthesis of allylic boronic ester **6b** via lithiation-borylation*</u>

To a solution of primary carbamate **18** (3.00 g, 17.3 mmol, 3 equiv.) and TMEDA (2.59 mL, 17.3 mmol, 3.0 equiv.) in Et_2O (85 mL) at −78°C was added *s*-BuLi (13.3 mL, 1.3 M in hexanes, 17.3 mmol, 3.0 equiv.) dropwise. The resulting mixture was stirred for 5 hours at −78 °C and then *trans*-1-propenylboronic acid pinacol ester (1.09 mL, 5.71 mmol, 1.0 equiv.) was added dropwise. The reaction mixture was further stirred at −78 °C for 1 hour. $MgBr_2$•OEt_2 in Et_2O, made as follows, was added to the reaction mixture and stirred for 10 min: *1,2-Dibromoethane (0.98 mL, 11 mmol, 2 equiv.) was added to a suspension of magnesium (278 mg, 11.4 mmol, 2 equiv.) in Et_2O (14 mL) at room temperature. The reaction flask was then placed into a water bath in order to control the exothermic reaction and was stirred for 2 h.* The reaction was then warmed to room temperature and refluxed for 16 hours. The reaction mixture was allowed to cool down to room temperature, filtered through a short silica pad (using pentane as eluent) and evaporated under reduced pressure at 0 °C. The crude reaction mixture contained a mixture of **6b** and *trans*-1-propenylboronic acid pinacol ester (9:1 by ^{1}H NMR). Purification was achieved by flash column chromatography (SiO_2, pentane: Et_2O, 97:3) to afford compound **6b** (as a single compound) as a colourless oil (331 mg, 30% yield), and a mixture of **6b** and vinyl boronic acid pinacol ester (430 mg, 83:17 ratio).
R_f = 0.42 (pentane: Et_2O, 19:1).

^{1}H NMR (400 MHz, $CDCl_3$) δ (ppm) 5.50 (ddq, *J* = 15.2, 7.1, 1.4 Hz, 1H), 5.37 (dqd, *J* = 15.2, 6.2, 1.2 Hz, 1H), 1.88 – 1.72 (m, 1H), 1.64 (dt, *J* = 6.2, 1.4 Hz, 3H), 1.23 (s, 12H), 1.04 (d, *J* = 7.4 Hz, 3H).
^{13}C NMR (101 MHz, $CDCl_3$) δ (ppm) 133.2 (CH), 122.7 (CH), 83.0 (2xC), 24.67 (2xCH_3), 24.62 (2xCH_3), 18.1 (CH_3), 15.2 (CH_3). *The carbon attached to boron was not observed due to quadrupolar relaxation.*
^{11}B NMR (96 MHz, $CDCl_3$) δ (ppm) 33.
Spectroscopic data are in accordance with the literature.[8]

c) General procedure for the synthesis of 4-hydroxy THPs **8a-g** (Table 1).

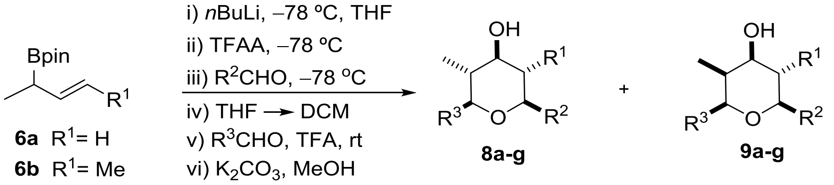

To a stirred solution of the corresponding allylic boronic ester (0.27 mmol, 1 equiv.) in THF (2.7 mL) at −78 °C under N_2 was added *n*-BuLi (1.5 M in hexanes, 0.20 mL, 0.30 mmol, 1.1 equiv.) dropwise, and the solution was allowed to stir for 15 mins. To this mixture was added TFAA (46 µL, 0.33 mmol, 1.2 equiv.) dropwise and the reaction was stirred for a further 30 min at −78 °C. The first corresponding aldehyde (0.29 mmol, 1.05 equiv.) was then added dropwise and the mixture was stirred at −78 °C for 2 h and then allowed to slowly warm up to room temperature overnight. The solvent was evaporated under high vacuum. After refilling the flask with N_2, the reaction mixture was dissolved in DCM (2.5 mL) and the second corresponding aldehyde (0.82 mmol equiv.) was added. After 5 mins of stirring, TFA (0.8 mL) was added at 0 °C and the reaction was then stirred at room temperature for 2 h. The reaction was quenched upon dropwise addition of an aqueous $NaHCO_3$ sat. solution. The two layers of the resulting biphasic solution were separated and the aqueous layer was extracted with DCM (x2). The combined organic layers were dried over $MgSO_4$ and the solvent was removed under reduced pressure. The residue was dissolved in MeOH (1 mL) and stirred with K_2CO_3 (57 mg, 0.41 mmol, 1.5 equiv.) for 30 mins. MeOH was then removed under reduced pressure and water (10 mL) was added. The mixture was extracted with DCM (x3), the combined organic layers were dried over $MgSO_4$ and the solvent was removed under reduced pressure. The resulting 4-hydroxytetrahydropyrans were purified by flash column chromatography.

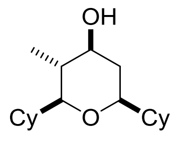

8a

Compound **8a** was obtained in 84% yield and 87:13 *dr* (65 mg, 0.23 mmol) as a white solid after purification by flash column chromatography (SiO_2, petroleum: EtOAc, 9:1) using allylic boronic ester **6a** (50 mg, 0.27 mmol),

[8]J. Whitesell, *J. Org. Chem.* **1985**, *50*, 4978.

cyclohexanecarboxaldehyde as the first (32 mg, 0.29 mmol) and second aldehyde (92 mg, 0.82 mmol), as described in the general procedure.

R_f= 0.18 (petroleum: EtOAc, 9:1).

^{1}H NMR (400 MHz, $CDCl_3$) δ (ppm) 3.29 (ddd, *J* = 10.8, 9.7, 4.7 Hz, 1H), 2.95 (ddd, *J* = 11.3, 6.6, 2.0 Hz, 1H), 2.69 (dd, *J* = 9.9, 1.8 Hz, 1H), 1.99 – 1.87 (m, 2H), 1.78 – 1.53 (m, 9H), 1.52 – 1.30 (m, 5H), 1.27 – 1.08 (m, 8H), 1.03 – 0.94 (m, 1H), 0.91 (d, *J* = 6.5 Hz, 3H).

^{13}C NMR (101 MHz, $CDCl_3$) δ (ppm) 84.4 (CH), 79.6 (CH), 74.8 (CH), 42.9 (CH), 40.4 (CH), 38.8 (CH), 38.4 (CH_2), 31.1 (CH_2), 29.0 (CH_2), 28.9 (CH_2), 27.0 (CH_2), 26.7 (CH_2), 26.6 (2xCH_2), 26.3 (CH_2), 26.1 (CH_2), 24.9 (CH_2), 12.3 (CH_3).

HRMS (ESI) calcd. for $C_{18}H_{32}O_2Na$ [M+Na]$^+$: 303.2295; found: 303.2299.

m.p.= 97-98 °C.

IR ν_{max} (neat)/cm^{-1}: 3441, 2917, 2849, 1443, 1378, 1339, 1096, 1055, 887, 635.

It should be noted that the *E/Z* ratio of the intermediate homoallylic alcohol leading to the formation of **8a** was 87:13. This was obtained in a similar but separate experiment:

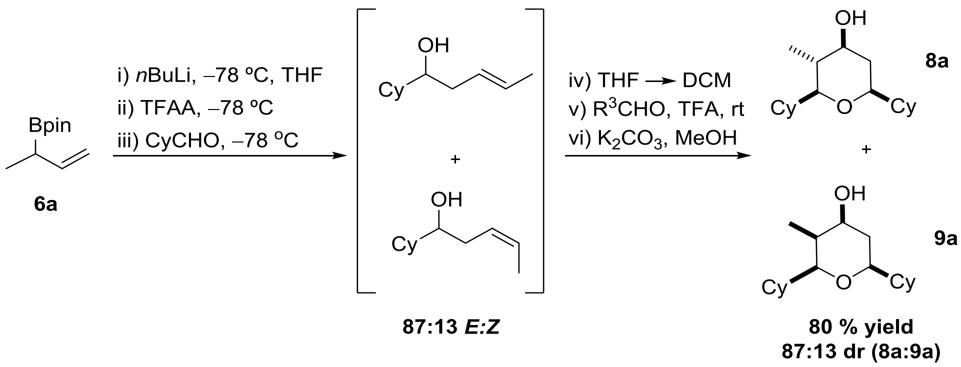

To a stirred solution of allylic boronic ester **6a** (60 mg, 0.33 mmol, 1 equiv.) in THF (3.3 mL) at −78 °C under N_2 was added *n*-BuLi (1.5 M in hexanes, 0.24 mL, 0.36 mmol, 1.1 equiv.) dropwise, and the solution was allowed to stir for 15 mins. To this mixture was added TFAA (56 µL, 0.40 mmol, 1.2 equiv.) dropwise and the reaction was stirred for a further 30 min at −78 °C. Cyclohexanecarboxaldehyde (39 mg, 0.35 mmol, 1.05 equiv.) was then added dropwise and the mixture was stirred at −78 °C for 2 h and then allowed to slowly warm up to room temperature overnight. The solvent was evaporated under high vacuum. After refilling the flask with N_2, the reaction mixture was dissolved in DCM (3.0 mL) and a ***0.5 mL*** *aliquot was removed from the reaction mixture: this aliquot was quenched with sat. NH_4Cl and extracted with DCM (x2). The combined organic layers were dried over $MgSO_4$, filtered and concentrated under reduced pressure – the E:Z ratio was determined to be* ***87:13*** *by ^{1}H NMR analysis of the crude aliquot mixture (see below for the spectrum).* Cyclohexanecarboxaldehyde (92 mg, 0.82 mmol, 3 equiv.) was then added to the remaining 2.5 mL bulk reaction mixture. After 5 mins of stirring, TFA (1 mL) was added at 0 °C and the reaction was then stirred at room temperature for 2 h. The reaction was quenched upon dropwise addition of an aqueous $NaHCO_3$ sat. solution. The two layers of the resulting biphasic solution were separated and the aqueous layer was extracted with DCM (x2). The combined organic layers were dried over $MgSO_4$ and the solvent was

removed under reduced pressure. The residue was dissolved in MeOH (1 mL) and stirred with K_2CO_3 (69 mg, 0.50 mmol, 1.5 equiv.) for 30 mins. MeOH was then removed under reduced pressure and water (10 mL) was added. The mixture was extracted with DCM (x3), the combined organic layers were dried over $MgSO_4$ and the solvent was removed under reduced pressure. The crude reaction mixture was purified by flash column chromatography (petroleum ether: EtOAc, 9:1) to give **8a** in 80% yield (based on the removal of 1/6 of the reaction mixture) and 87:13 dr as a colourless oil (62 mg, 0.22 mmol).

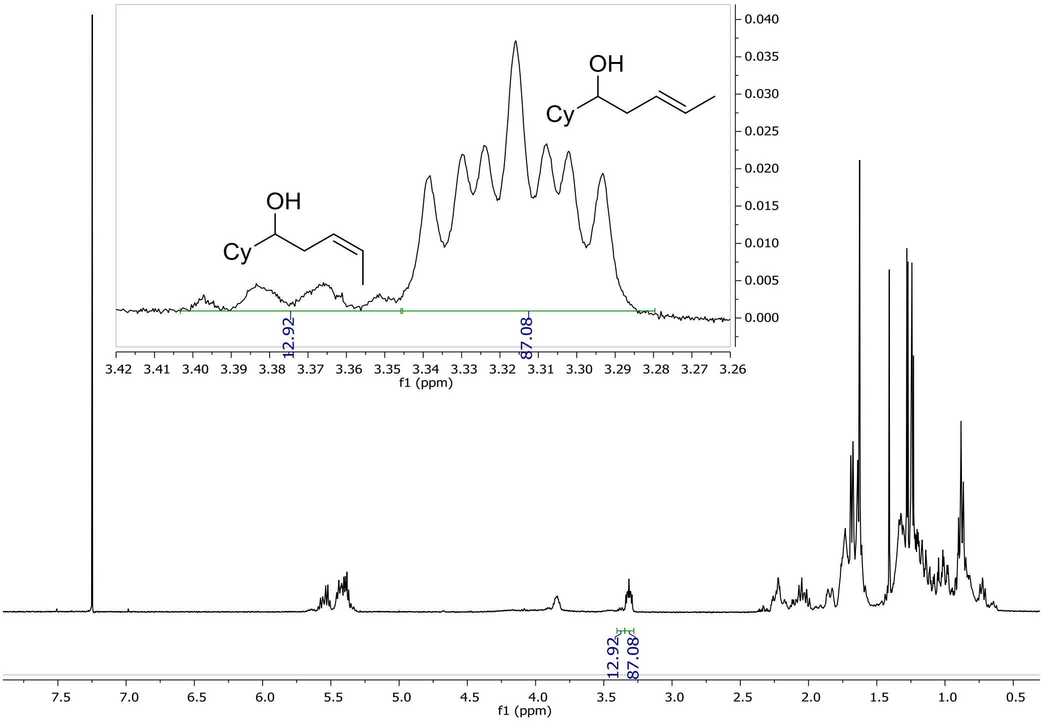

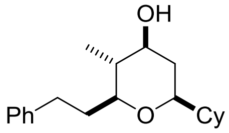

8b

Compound **8b** was obtained in 75% yield and 87:13 *dr* (62 mg, 0.21 mmol) as a colourless oil after purification by flash column chromatography (SiO_2, petroleum: EtOAc, 9:1) using allylic boronic ester **6a** (50 mg, 0.27 mmol), cyclohexanecarboxaldehyde as the first aldehyde (32 mg, 0.29 mmol) and hydrocinnamaldehyde as the second aldehyde (111 mg, 0.82 mmol), as described in the general procedure.

R_f = 0.16 (petroleum: EtOAc, 9:1).

^{1}H NMR (400 MHz, $CDCl_3$) δ (ppm) 7.48 – 7.03 (m, 5H), 3.28 (ddd, *J* = 10.9, 9.8, 4.8 Hz, 1H), 3.01 (ddd, *J* = 11.3, 7.3, 1.9 Hz, 1H), 2.94 – 2.81 (m, 2H), 2.73 – 2.62 (m, 1H), 2.15 – 2.06 (m, 1H), 2.02 (ddd, *J* = 12.3, 4.8, 1.9 Hz, 1H), 1.99 – 1.88 (m, 1H), 1.84 – 1.60 (m, 5H), 1.48 – 1.36 (m, 1H), 1.34 – 1.11 (m, 5H), 1.10 – 0.95 (m, 2H), 0.92 (d, *J* = 6.5 Hz, 3H).

^{13}C NMR (101 MHz, $CDCl_3$) δ (ppm) 142.6 (C), 128.6 (2xCH), 128.3 (2xCH), 125.6 (CH), 79.6 (CH), 79.4 (CH), 74.2 (CH), 44.3 (CH), 43.0 (CH), 38.6 (CH_2), 34.9 (CH_2), 31.8 (CH_2), 29.4 (CH_2), 28.9 (CH_2), 26.6 (CH_2), 26.2 (CH_2), 26.0 (CH_2), 12.8 (CH_3).

HRMS (ESI) calcd. for $C_{20}H_{30}O_2Na$ $[M+Na]^+$: 325.2138; found: 325.2146.

IR ν_{max} (neat)/cm^{-1}: 3362, 3026, 2921, 2851, 1603, 1495, 1367, 1150, 1038, 697.

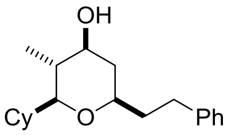

8c

Compound **8c** was obtained in 57% yield and 89:11 *dr* (50 mg, 0.15 mmol) as a colourless oil after purification by flash column chromatography (SiO_2, petroleum: EtOAc, 9:1) using allylic boronic ester **6a** (50 mg, 0.27 mmol), hydrocinnamaldehyde as the first aldehyde (39 mg, 0.29 mmol) and cyclohexanecarboxaldehyde as the second aldehyde (92 mg, 0.82 mmol), as described in the general procedure.

R_f = 0.16 (petroleum: EtOAc, 9:1).

^{1}H NMR (400 MHz, CDCl$_3$) δ (ppm) 7.45 – 7.09 (m, 5H), 3.29 (td, *J* = 10.3, 4.6 Hz, 1H), 3.21 (dddd, *J* = 11.1, 9.0, 3.7, 2.0 Hz, 1H), 2.83 – 2.65 (m, 3H), 1.91 – 1.74 (m, 4H), 1.71 – 1.57 (m, 4H), 1.57 – 1.37 (m, 3H), 1.31 – 1.11 (m, 5H), 0.93 (d, *J* = 6.5 Hz, 3H).

^{13}C NMR (101 MHz, CDCl$_3$) δ (ppm) 142.4 (C), 128.6 (2xCH), 128.2 (2xCH), 125.6 (CH), 84.3 (CH), 74.2 (CH), 73.8 (CH), 41.5 (CH_2), 40.4 (CH), 38.7 (CH), 37.7 (CH_2), 31.7 (CH_2), 31.2 (CH_2), 27.0 (CH_2), 26.6 (2xCH_2), 24.9 (CH_2), 12.3 (CH_3).

HRMS (ESI) calcd. for $C_{20}H_{30}O_2Na$ [M+Na]$^+$: 325.2138; found 325.2152.

IR ν_{max} (neat)/cm^{-1}: 3428, 3029, 2922, 2852, 1604, 1498, 1340, 1149, 1011, 694.

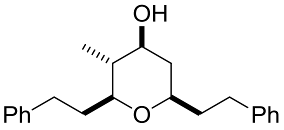

8d

Compound **8d** was obtained in 65% yield and 88:12 *dr* (58 mg, 0.18 mmol) as a colourless oil after purification by flash column chromatography (SiO_2, petroleum: EtOAc, 8:2) using allylic boronic ester **6a** (50 mg, 0.27 mmol), hydrocinnamaldehyde as the first (39 mg, 0.29 mmol) and the second aldehyde (111 mg, 0.82 mmol), as described in the general procedure.

R_f = 0.33 (petroleum: EtOAc, 6:4).

^{1}H NMR (400 MHz, CDCl$_3$) δ (ppm) 7.36 – 7.15 (m, 10H), 3.38 – 3.20 (m, 2H), 3.04 – 2.82 (m, 3H), 2.82 – 2.65 (m, 2H), 2.08 – 1.88 (m, 3H), 1.87 – 1.69 (m, 2H), 1.41 – 1.19 (m, 2H), 0.96 (d, *J* = 6.5 Hz, 3H).

^{13}C NMR (101 MHz, CDCl$_3$) δ (ppm) 142.5 (C), 142.1 (C), 128.53 (2xCH), 128.52 (2xCH), 128.35 (2xCH), 128.33 (2xCH), 125.8 (CH), 125.7 (CH), 79.7 (CH), 74.1 (CH), 73.6 (CH), 44.2 (CH), 41.4 (CH_2), 37.8 (CH_2), 34.9 (CH_2), 31.92 (CH_2), 31.88 (CH_2), 12.8 (CH_3).

HRMS (ESI) calcd. for $C_{22}H_{28}O_2Na$ [M+Na]$^+$: 347.1982; found 347.1992.

IR ν_{max} (neat)/cm^{-1}: 3379, 3061, 2921, 2854, 1603, 1495, 1373, 1097, 1028, 747.

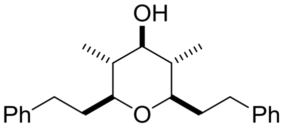

8e

Compound **8e** was obtained in 89% yield and 90:10 *dr* (81 mg, 0.24 mmol) as a pale yellow oil after purification by flash column chromatography (SiO_2, petroleum ether: EtOAc, 9:1) using allylic boronic ester **6b** (53 mg, 0.27 mmol), hydrocinnamaldehyde as the first aldehyde (39 mg, 0.29 mmol) and as the second aldehyde (111 mg, 0.82 mmol), as described in the general procedure.

R_f = 0.37 (petroleum ether: EtOAc, 7:3).

^{1}H NMR (400 MHz, $CDCl_3$) δ (ppm) 7.32 – 7.27 (m, 4H), 7.25 – 7.17 (m, 6H), 3.05 – 2.89 (m, 4H), 2.82 (t, *J* = 9.7 Hz, 1H), 2.72 (ddd, *J* = 13.7, 9.5, 7.0 Hz, 2H), 1.99 (dddd, *J* = 13.9, 9.7, 7.1, 2.5 Hz, 2H), 1.76 (dtd, *J* = 14.3, 9.6, 4.9 Hz, 2H), 1.48 (br. s, 1H), 1.35 (tq, *J* = 9.9, 6.6 Hz, 2H), 0.95 (d, *J* = 6.6 Hz, 6H).

^{13}C NMR (101 MHz, $CDCl_3$) δ (ppm) 142.5 (2xC), 128.5 (4xCH), 128.3 (4xCH), 125.7 (2xCH), 79.5 (2xCH), 79.3 (CH), 44.0 (2xCH), 35.1 (2xCH_2), 32.0 (2xCH_2), 13.3 (2xCH_3).

HRMS (ESI) calcd. for $C_{23}H_{30}O_2Na$ $[M+Na]^+$: 361.2138; found: 361.2142.

IR ν_{max} (neat)/cm^{-1}: 3366, 2957, 2923, 1496, 1454, 1373, 1125, 1089, 731, 697.

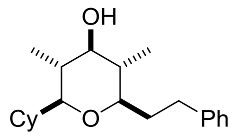

8f

Compound **8f** was obtained in 81% yield and 91:9 *dr* (70 mg, 0.22 mmol) as a colourless oil after purification by flash column chromatography (SiO_2, petroleum: EtOAc, 85:15) using allylic boronic ester **6a** (53 mg, 0.27 mmol), hydrocinnamaldehyde as the first aldehyde (39 mg, 0.29 mmol) and cyclohexanecarboxaldehyde as the second aldehyde (92 mg, 0.82 mmol), as described in the general procedure.

R$_f$ = 0.44 (petroleum: EtOAc, 7:3).

^{1}H NMR (400 MHz, $CDCl_3$) δ (ppm) 7.31 – 7.23 (m, 2H), 7.22 – 7.11 (m, 3H), 2.92 – 2.75 (m, 4H), 2.65 (ddd, *J* = 13.8, 9.0, 7.5 Hz, 1H), 1.90 (dddd, *J* = 13.8, 9.3, 7.5, 2.5 Hz, 1H), 1.81 – 1.56 (m, 7H), 1.56 – 1.41 (m, 4H), 1.35 – 1.13 (m, 4H), 0.92 (d, *J* = 6.7 Hz, 3H), 0.90 (d, *J* = 6.6 Hz, 3H).

^{13}C NMR (101 MHz, $CDCl_3$) δ (ppm) 142.8 (C), 128.5 (2xCH), 128.2 (2xCH), 125.6 (CH), 84.1 (CH), 79.8 (CH), 79.4 (CH), 43.9 (CH), 40.1 (CH), 38.9 (CH), 35.0 (CH_2), 31.6 (CH_2), 31.2 (CH_2), 27.0 (CH_2), 26.7 (CH_2), 26.6 (CH_2), 24.9 (CH_2), 13.3 (CH_3), 12.8 (CH_3).

HRMS (ESI) calcd. for $C_{21}H_{32}O_2Na$ $[M+Na]^+$: 339.2295; found: 339.2289.

IR ν_{max} (neat)/cm^{-1}: 3338, 3026, 2925, 2852, 1451, 1377, 1097, 1028, 734, 698.

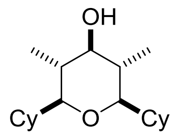

8g

Compound **8g** was obtained in 86% yield and 88:12 *dr* (69 mg, 0.23 mmol) as a colourless oil after purification by flash column chromatography (SiO_2, petroleum ether: EtOAc, 9:1) using allylic boronic ester **6b** (53 mg, 0.27 mmol), cyclohexanecarboxaldehyde as the first aldehyde (32 mg, 0.29 mmol) and as the second aldehyde (92 mg, 0.82 mmol), as described in the general procedure.

R$_f$ = 0.22 (petroleum ether: EtOAc, 9:1).

^{1}H NMR (400 MHz, $CDCl_3$) δ (ppm) 2.83 (t, *J* = 9.6 Hz, 1H), 2.70 (dd, *J* = 10.0, 1.8 Hz, 2H), 1.78 – 1.68 (m, 4H), 1.67 – 1.60 (m, 2H), 1.59 – 1.32 (m, 11H), 1.26 – 1.07 (m, 8H), 0.89 (d, *J* = 6.5 Hz, 6H).

^{13}C NMR (101 MHz, $CDCl_3$) δ (ppm) 84.2 (2xCH), 80.3 (CH), 40.0 (2xCH), 39.0 (2xCH), 31.1 (2xCH_2), 27.0 (2xCH_2), 26.7 (2xCH_2), 26.6 (2xCH_2), 24.9 (2xCH_2), 12.8 (2xCH_3).

HRMS (ESI) calcd. for $C_{19}H_{34}O_2Na$ $[M+Na]^+$: 317.2451; found: 317.2451.

IR ν_{max} (neat)/cm^{-1}: 3314, 2926, 2851, 1449, 1376, 1183, 1101, 1028, 994, 734.

It should be noted that no detrimental effect to the yield and diastereoselectivity was observed when this allylboration-Prins reaction protocol was conducted using a mixture of allylic boronic ester **6a** and vinyl boronic acid pinacol ester.

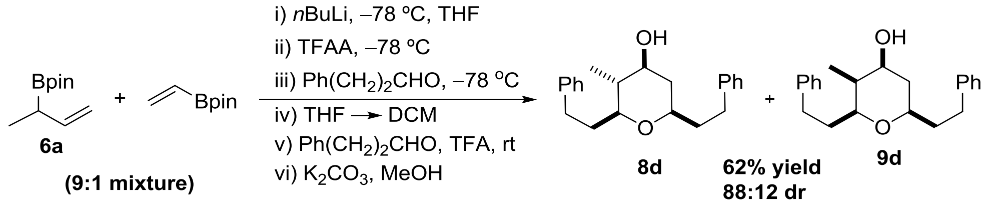

To a stirred solution of allylic boronic ester **6a**:vinyl boronic acid pinacol ester (9:1 mixture, 60 mg, 0.33 mmol {i.e. 0.30 mmol of **6a**}, 1 equiv.) in THF (3.3 mL) at −78 °C under N_2 was added *n*-BuLi (1.6 M in hexanes, 0.23 mL, 0.37 mmol, 1.1 equiv.) dropwise, and the solution was allowed to stir for 15 mins. To this mixture was added TFAA (57 µL, 0.40 mmol, 1.2 equiv.) dropwise and the reaction was stirred for a further 30 min at −78 °C. Hydrocinnamaldehyde (47 mg, 0.35 mmol, 1.05 equiv.) was then added dropwise and the mixture was stirred at −78 °C for 2 h and then allowed to slowly warm up to room temperature overnight. The solvent was evaporated under high vacuum. After refilling the flask with N_2, the reaction mixture was dissolved in DCM (3.0 mL) and hydrocinnamaldehyde (135 mg, 1.00 mmol, 3 equiv.) was added. After 5 mins of stirring, TFA (1 mL) was added at 0 °C and the reaction was then stirred at room temperature for 2 h. The reaction was quenched upon dropwise additi on of an aqueous $NaHCO_3$ sat. solution. The two layers of the resulting biphasic solution were separated and the aqueous layer was extracted with DCM (x2). The combined organic layers were dried over $MgSO_4$ and the solvent was removed under reduced pressure. The residue was dissolved in MeOH (1 mL) and stirred with K_2CO_3 (69 mg, 0.50 mmol, 1.5 equiv.) for 30 mins. MeOH was then removed under reduced pressure and water (10 mL) was added. The mixture was extracted with DCM (x3), the combined organic layers were dried over $MgSO_4$ and the solvent was removed under reduced pressure. The crude reaction mixture was purified by flash column chromatography (petroleum ether: EtOAc, 9:1) to give **8e** in 61% yield (based on the use of 0.30 mmol of **6a**) and 88:12 dr as a colourless oil (60 mg, 0.19 mmol).

4. Total synthesis of (−)-clavosolide A

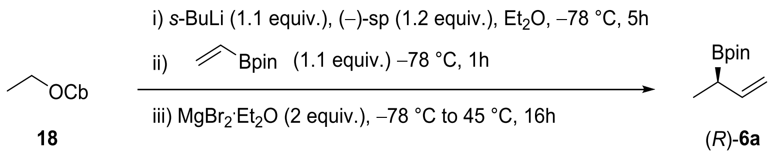

To a solution of primary carbamate **18** (1.00 g, 5.78 mmol, 1 equiv.) and (−)-sparteine (1.6 mL, 6.9 mmol, 1.2 equiv.) in Et_2O (29 mL) at −78 °C was added *s*-BuLi (1.3 M in hexanes, 4.9 mL, 6.4 mmol, 1.1 equiv.) dropwise. The resulting mixture was stirred for 5 h at −78 °C and then vinyl boronic acid pinacol ester (1.08 mL, 6.36 mmol, 1.1 equiv.) was added. The reaction mixture was further stirred at −78 °C for 1 hour. $MgBr_2$•OEt_2 in Et_2O, made as follows, was added to the reaction mixture and stirred for 10 min: *1,2-Dibromoethane (0.96 mL, 12 mmol, 2 equiv.) was added to a suspension of magnesium (277 mg, 11.6 mmol, 2 equiv.) in Et_2O (14 mL) at room temperature. The reaction flask was then placed into a water bath in order to control the exothermic reaction and was stirred for 2 h.* The reaction was then warmed to room temperature and refluxed overnight. The reaction mixture was allowed to cool down to room temperature, filtered through a short silica pad (using pentane as eluent) and evaporated under reduced pressure at 0 °C. The crude reaction mixture was purified by flash column chromatography (SiO_2, pentane: Et_2O, 20:1) to afford compound (*R*)-**6a** (915 mg, 87% yield) as a colourless oil. *e.r.*= 96:4[9]

<u>*Note 1*</u>: In order to obtain the compound in good yield, the chromatography should be completed within 10 min., since the allylboronic ester decomposes readily on silica gel.

<u>*Note 2*</u>: Occasionally up to 10% of vinyl boronic ester can be obtained together with the allyl boronic ester.

<u>*Note 3*</u>: (−)-sp was recovered from the silica pad by washing it with 2M HCl, then the aqueous solution was basified with NaOH (pH=10), extracted with EtOAc and concentrated.

R_f = 0.35 (pentane: Et_2O, 19:1).

^{1}H NMR (301 MHz, $CDCl_3$) δ (ppm) 5.93 (ddd, *J* = 17.4, 10.3, 7.1 Hz, 1H), 5.03 – 4.83 (m, 2H), 1.96 – 1.79 (m, 1H), 1.22 (s, 12H), 1.09 (d, *J* = 7.3 Hz, 3H).

^{13}C NMR (76 MHz, $CDCl_3$) δ (ppm) 141.0 (CH), 112.0 (CH_2), 83.3 (2C), 24.75 (2xCH_3), 24.72 (2xCH_3), 22.4 (CH), 14.2 (CH_3).

^{11}B NMR (96 MHz, $CDCl_3$) δ (ppm) 33.

$[\alpha]_D^{22}$= +11.1 (*c* 0.8, $CHCl_3$).

Spectroscopic data are in accordance with the literature.[7]

[9] See section 7 for details about the e.r. determination.

Synthesis of aldehydes 7a and 7b

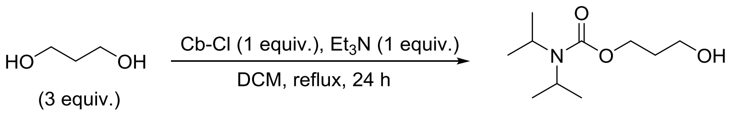

19a

To a solution of *N*,*N*-diisopropylcarbamoyl chloride (4.91 g, 30.0 mmol, 1 equiv.) and Et_3N (4.60 mL, 33.0 mmol, 1.05 equiv.) in CH_2Cl_2 (30 mL) was added 1,3-propanediol (6.85 g, 90.0 mmol, 3 equiv.). This mixture was then heated to reflux and stirred for 24 h. The reaction was cooled to room temperature and H_2O was added. The aqueous layer was extracted with CH_2Cl_2 (x3) and the combined organic layers were dried over $MgSO_4$ and evaporated under reduced pressure. The crude oil was purified by flash column chromatography (SiO_2, petroleum ether: EtOAc, 6:4). The monoprotected alcohol **19a** was obtained in 89% yield (5.44 g, 26.7 mmol) as a colourless oil.

$\boldsymbol{R_f}$ = 0.40 (petroleum ether: EtOAc, 4:6).

^{1}H NMR (400 MHz, $CDCl_3$) δ (ppm) δ 4.24 (t, *J* = 6.0 Hz, 2H), 4.15 – 3.88 (br. s, 2H), 3.62 (q, *J* = 6.0 Hz, 2H), 2.76 (t, *J* = 6.2 Hz, 1H), 1.82 (quint, *J* = 5.9 Hz, 2H), 1.20 (d, *J* = 6.8 Hz, 12H).

^{13}C NMR (76 MHz, $CDCl_3$) δ (ppm) 156.6 (C), 60.7 (CH_2), 58.5 (CH_2), 45.7 (2xCH), 32.5 (CH_2), 20.9 (4xCH_3).

HRMS (ESI) calc´d for $C_{10}H_{21}NNaO_3$ $[M+Na]^+$: 226.1414; found: 226.1414.

IR ν_{max} (neat)/cm^{-1}: 3444, 2967, 1664, 1437, 1134, 1290, 1064, 772.

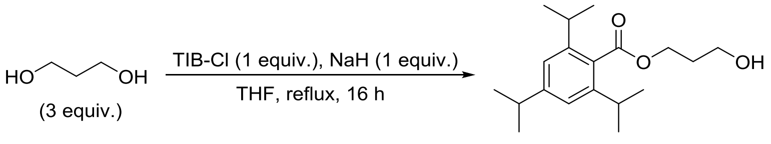

19b

To a flame-dried flask charged with NaH (60% dispersion in mineral oil) (400 mg, 10.0 mmol, 1 equiv.) under N_2 was added THF (30 mL). The solution was cooled to 0 °C and 1,3-propanediol (2.28 g, 30.0 mmol, 3 equiv.) was added dropwise. The mixture was stirred for 1 h at 0 °C and then 1 h at room temperature. TIB-Cl (2.67 g, 10.0 mmol, 1 equiv.) was added portionwise and after 30 min at room temperature the reaction was refluxed for 24 h. The reaction mixture was allowed to warm to room temperature and quenched with water. EtOAc was added, the layers were separated and the aqueous phase was extracted with EtOAc (x3). The combined organic layers were washed with 1M HCl (x2), 1M NaOH (x2), water (x1) and brine (x1), dried and evaporated under reduced pressure and purified by flash column chromatography (SiO_2, petroleum ether: EtOAc, 8:2) to give the corresponding alcohol **19b** in 84% yield (2.57 g, 8.40 mmol) as a colourless oil.

R_f = 0.41 (petroleum ether: EtOAc, 6:4).

^{1}H NMR (400 MHz, $CDCl_3$) δ (ppm) 7.00 (s, 2H), 4.44 (t, *J* = 6.3 Hz, 2H), 3.76 (t, *J* = 6.1 Hz, 2H), 2.94 – 2.77 (m, 3H), 2.02 – 1.93 (m, 2H), 1.23 (d, *J* = 6.9 Hz, 18H).

^{13}C NMR (100 MHz, $CDCl_3$) δ (ppm) 171.3 (C), 150.3 (C), 144.8 (2xC), 130.4 (C), 121.0 (2xCH), 62.1, (CH) 59.5 (CH), 34.5 (CH_2), 31.8 (CH), 31.6 (2xCH), 24.2 (4xCH_3), 24.0 (2xCH_3).

HRMS (ESI) calc'd for $C_{19}H_{30}NaO_3$ $[M+Na]^+$ 329.2087; found: 329.2090.

IR ν_{max} (neat)/cm^{-1}: 3442, 2960, 2871, 1724, 1606, 1461, 1250, 1290, 1069, 876.

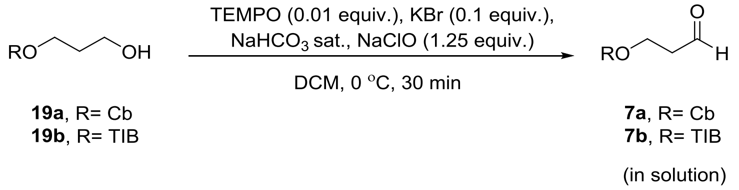

Following the procedure described by Anelli et al,[10] a flask at 0 °C was charged with a 0.8 M solution of alcohol **19a** or **19b** (1 equiv.) in DCM, a 0.008 M solution of TEMPO (0.01 equiv.) in DCM and a 0.5 M aqueous solution of KBr (0.1 equiv.). A 0.35 M aqueous NaOCl (1.25 equiv.) at pH 8.6 (adjusted with $NaHCO_3$) was added and the mixture was stirred vigorously for 30 min. The organic phase was separated, dried over $MgSO_4$ and filtered over a short silica pad after dilution with Et_2O. THF* was added and the solution was evaporated under reduced pressure at room temperature (crudely removing the Et_2O and DCM) until the volume of the solution approximately matched the volume of THF initially added. The aldehyde **7a** or **7b** was directly submitted to the next reaction without further purification as a solution in THF (0.5 M).

*<u>*Note 1*</u>*: The amount of THF added is the amount required to obtain a 0.5 M solution of the aldehyde **7a** or **7b**.

<u>Note 2</u>: Aldehydes **7a** or **7b** were prepared prior to use due to it instability and should be kept in solution. Evaporation until dryness causes decomposition of the aldehydes.

<u>Note 3</u>: An 80% yield was considered to conduct the next reaction.

Lewis-base mediated allylboration

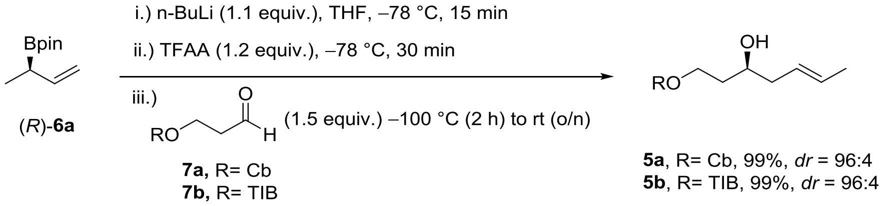

[10] P. L. Anelli, C. Biffi, F. Montanari, S. Quici, *J. Org. Chem.* **1987**, *52*, 2559.

To a solution of allylic boronic ester (*R*)-**6a** (1 equiv.) in THF (0.1 M) at −78 °C under N_2 was added *n*-BuLi (1.52 M in hexane, 1.1 equiv.) dropwise and the solution allowed to stir for 15 min. To this mixture was added TFAA (1.2 equiv.) dropwise and the reaction was stirred for a further 30 min at −78 °C. The mixture was cooled down to −100 °C. A 0.5M solution of freshly prepared aldehyde **7a**/**7b** (1.5 equiv.) in THF was pre-cooled to −78 °C and added to the reaction dropwise. The mixture was kept at −100 °C for 2 h and allowed to slowly warm up to rt overnight. The reaction was quenched with aq. sat. NH_4Cl and extracted with EtOAc (x3). The combined organic layers were washed with brine (x2), dried over $MgSO_4$, filtered, evaporated under reduced pressure and purified by flash column chromatography.

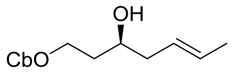

5a

Compound **5a** was obtained as a colourless oil in 99% yield (96:4 *dr*)(254 mg, 0.99 mmol) by flash column chromatography (SiO_2, petroleum ether: EtOAc, 8:2) using allylic boronic ester (*R*)-**6a** (182 mg, 1.00 mmol), *n*-BuLi (0.72 mL, 1.1 mmol), TFAA (0.17 mL, 1.2 mmol) and **7a** (301 mg, 1.50 mmol).

$\boldsymbol{R_f}$ = 0.40 (petroleum ether: EtOAc, 3:1).

^{1}H NMR (500 MHz, $CDCl_3$) δ (ppm) 5.62 – 5.51 (m, 1H), 5.52 – 5.38 (m, 1H), 4.47 – 4.35 (m, 1H), 4.19 – 4.08 (m, 1H), 4.08 – 3.77 (br. s, 2H), 3.72 – 3.63 (m, 1H), 2.54 (br. s, 1H), 2.27 – 2.12 (m, 2H), 1.90 – 1.79 (m, 1H), 1.71 (d, *J*= 6.2 Hz, 3H), 1.68 – 1.63 (m, 1H), 1.22 (d, *J*= 6.8 Hz, 12H).

^{13}C NMR (125 MHz, $CDCl_3$) δ (ppm) 156.2 (C), 128.6 (CH), 127.0 (CH), 67.7 (CH), 61.5 (CH_2), 46.1 (2xCH), 40.5 (CH_2), 36.6 (CH_2), 21.2 (4xCH_3), 18.1 (CH_3).

HRMS (ESI) calc´d for $C_{14}H_{27}NNaO_3$ $[M+Na]^+$: 280.1883; found: 280.1884.

IR ν_{max} (neat)/cm^{-1}: 3418, 2966, 2932, 1669, 1299, 1062, 966, 772.

$[\alpha]_D^{22}$= +5.8 (*c* 1.3, $CHCl_3$).

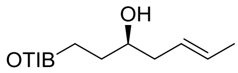

5b

Compound **5b** was obtained as a colourless oil in 99% yield (96:4 *dr*) (356 mg, 0.99 mmol) by flash column chromatography (SiO_2, petroleum ether: EtOAc, 8:2) using allylic boronic ester (*R*)-**6a** (182 mg, 1.00 mmol), *n*-BuLi (0.72 mL, 1.1 mmol), TFAA (0.17 mL, 1.2 mmol) and **7b** (456 mg, 1.50 mmol).

$\boldsymbol{R_f}$ = 0.33 (petroleum ether: EtOAc, 8:2).

^{1}H NMR (500 MHz, $CDCl_3$) δ (ppm) δ 7.04 (s, 2H), 5.65 – 5.54 (m, 1H), 5.51 – 5.39 (m, 1H), 4.59 – 4.50 (m, 1H), 4.49 – 4.42 (m, 1H), 3.80 (br. s, 1H), 2.98 – 2.82 (m, 3H), 2.33 – 2.22 (m, 1H), 2.22 – 2.12 (m, 1H), 2.05 – 1.90 (m, 2H), 1.89 – 1.76 (m, 1H), 1.72 (d, *J* = 6.3 Hz, 3H), 1.28 (d, *J* = 6.9 Hz, 18H).

^{13}C NMR (125 MHz, $CDCl_3$) δ (ppm) 171.6 (C), 150.8 (C), 145.4 (2xC), 131.1 (C), 130.1 (CH), 127.2 (CH), 121.5 (2xCH), 68.9 (CH), 62.9 (CH_2), 41.7 (CH_2), 36.5 (CH_2), 35.3 (CH), 32.4 (2xCH), 25.02 (2xCH_3), 24.99 (2xCH_3), 24.8 (2xCH_3), 18.93 (CH_3).

HRMS (ESI) calc´d for $C_{23}H_{36}NaO_3$ $[M+Na]^+$: 383.2557; found: 383.2566.

IR ν_{max} (neat)/cm^{-1}: 3444, 2960, 2928, 2870, 1724, 1250, 1073, 968, 876.

$[\alpha]_D^{22}$= +4.0 (*c* 1, $CHCl_3$).

Three-component Lewis base mediated allylboration- Prins cyclisation

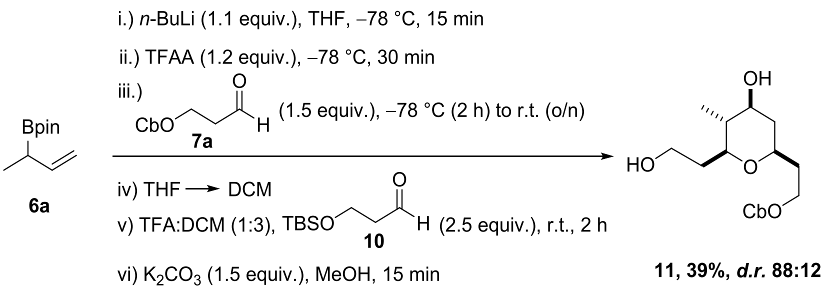

To a stirred solution of allylic boronic ester **6a** (199 mg, 1.09 mmol, 1 equiv.) in THF (1.1 mL) at −78 °C under N_2 was added *n*-BuLi (1.6 M in hexanes, 0.75 mL, 1.1 equiv.) dropwise and the solution was allowed to stir for 15 min. To this mixture was added TFAA (0.18 mL, 1.3 mmol, 1.2 equiv.) dropwise and the reaction was stirred for further 30 min at −78 °C. A 0.5 M solution of freshly prepared aldehyde **7a** (329 mg, 1.63 mmol, 1.5 equiv.) in THF was added to the reaction dropwise. The mixture was kept at −78 °C for 2 h and allowed to slowly warm up to room temperature overnight. The solvent was then evaporated under high vacuum and dry DCM was added and subsequently evaporated (x2). After refilling the flask with N_2, the reaction mixture was dissolved in DCM (7.2 mL) and **10** (4 equiv.) was added. After 5 mins of stirring, TFA (1.8 mL) was added at 0 °C and the reaction was then stirred at room temperature for 2 h. The reaction was quenched upon dropwise addition of an aqueous $NaHCO_3$ solution. The two layers of the resulting biphasic solution were separated and the aqueous layer was extracted with DCM (x2). The combined organic layers were dried over $MgSO_4$ and the solvent was removed under reduced pressure. The residue was dissolved in MeOH (3.6 mL) and stirred with K_2CO_3 (226 mg, 1.63 mmol, 1.5 equiv.) for 30 mins. MeOH was then removed under reduced pressure and water (10 mL) was added. The mixture was extracted with DCM (x3), the combined organic layers were dried over $MgSO_4$ and the solvent was removed under reduced pressure. The residue was purified by flash column chromatography (SiO_2, petroleum ether: EtOAc, 1:9) to give compound **11** as a colourless oil in 39% yield and 88:12 dr (141 mg, 0.425 mmol).

R_f = 0.29 (EtOAc).

^{1}H NMR (500 MHz, $CDCl_3$) δ (ppm) 4.35 – 4.26 (m, 1H), 3.95 (dt, *J* = 11.0, 5.6 Hz, 1H), 3.80 – 3.73 (m, 1H), 3.65 – 3.58 (m, 1H), 3.36 – 3.28 (m, 1H), 3.27 – 3.19 (m, 1H), 3.08 (td, *J* = 9.8, 2.8 Hz, 1H), 1.96 (br. s, 1H), 1.86 (m, 2H), 1.71 (dt, *J* = 7.1, 5.9 Hz, 2H), 1.54 – 1.42 (m, 1H), 1.26 – 1.13 (m, 2H), 1.09 (d, *J* = 6.8 Hz, 12H), 0.87 (d, *J* = 6.0 Hz, 3H). Signals from the N(**CH**CH_3)$_2$ are not observed.

^{13}C NMR (125 MHz, $CDCl_3$) δ (ppm) 155.6 (C), 79.7 (CH), 73.2 (CH), 72.1 (CH), 60.7 (CH_2), 59.9 (CH_2), 46.0 (2xCH), 43.9 (CH), 41.0 (CH_2), 35.8 (CH_2), 34.9 (CH_2), 20.8 (4xCH_3), 12.6 (CH_3).

HRMS (ESI) calc´d for $C_{17}H_{33}NNaO_5$ $[M+Na]^+$: 354.2251; found: 354.2260.

IR ν_{max} (neat)/cm^{-1}: 3406, 2967, 1667, 1439, 1300, 1134, 1048, 772, 605.

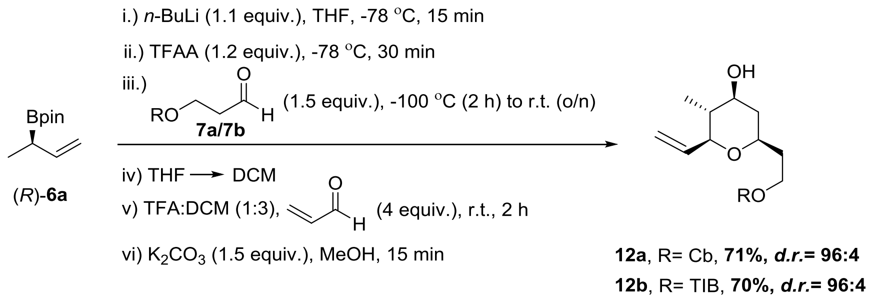

To a stirred solution of allylic boronic ester (*R*)-**6a** (1 equiv.) in THF (0.1 M) at −78 °C under N_2 was added *n*-BuLi (1.6 M in hexanes, 1.1 equiv.) dropwise and the solution was allowed to stir for 15 min. To this mixture was added TFAA (1.2 equiv.) dropwise and the reaction was stirred for further 30 min at −78 °C. The mixture was cooled down to −100 °C and a 0.5M solution of freshly prepared aldehyde **7a**/**7b** (1.5 equiv.) in THF was pre-cooled to −78 °C and added to the reaction dropwise. The mixture was kept at −100 °C for 2 h and allowed to slowly warm up to room temperature overnight. The solvent was then evaporated under high vacuum and dry DCM was added and subsequently evaporated (x2). After refilling the flask with N_2, the reaction mixture was dissolved in DCM (0.15 M) and acrolein (4 equiv.) was added. After 5 mins of stirring, TFA (1/3 of DCM volume) was added at 0 °C and the reaction was then stirred at room temperature for 2 h. The reaction was quenched upon dropwise addition of an aqueous $NaHCO_3$ solution. The two layers of the resulting biphasic solution were separated and the aqueous layer was extracted with DCM (x2). The combined organic layers were dried over $MgSO_4$ and the solvent was removed under reduced pressure. The residue was dissolved in MeOH (0.3 M) and stirred with K_2CO_3 (1.5 equiv.) for 30 mins. MeOH was then removed under reduced pressure and water (10 mL) was added. The mixture was extracted with DCM (x3), the combined organic layers were dried over $MgSO_4$ and the solvent was removed under reduced pressure. The residue was purified by flash column chromatography.

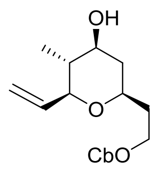

12a

Compound **12a** was obtained as a colourless oil in 75% yield (96:4 *dr*) (854 mg, 2.73 mmol) by flash column chromatography (SiO_2, petroleum ether: EtOAc, 6:4) from allyl boronic ester (*R*)-**6** (700 mg, 3.85 mmol), *n*-BuLi (2.64 mL, 4.23 mmol), TFAA (0.63 mL, 4.62 mmol), **7a** (1.16 g, 5.77 mmol), acrolein (1.03 mL, 15.40 mmol) and K_2CO_3 (796 mg, 5.77 mmol).

$\boldsymbol{R_f}$ = 0.27 (petroleum ether: EtOAc, 6:4).

^{1}H NMR (500 MHz, $CDCl_3$) δ (ppm) 5.79 (ddd, *J* = 17.4, 10.4, 7.2 Hz, 1H), 5.24 (dd, *J* = 17.2, 10.4 Hz, 2H), 4.26 – 4.13 (m, 2H), 4.12 – 3.68 (br. s, 2H), 3.55 (dddd, *J* = 11.5, 7.3, 4.8, 1.8 Hz, 1H), 3.46 – 3.33 (m, 2H), 2.01 (ddd, *J* = 12.4, 4.7, 1.9 Hz, 1H), 1.98 – 1.90 (m, 2H), 1.84 (dtd, *J* = 12.4, 7.0, 4.8 Hz, 1H), 1.36 (app q, 1H), 1.31 – 1.25 (m, 1H), 1.20 (d, *J* = 6.9 Hz, 12H), 0.97 (d, *J* = 6.6 Hz, 3H).

^{13}C NMR (125 MHz, $CDCl_3$) δ (ppm) 155.8 (C), 136.8 (CH), 117.7 (CH_2), 82.9 (CH), 73.5 (CH), 72.5 (CH), 61.5 (CH_2), 45.7 (2xCH), 43.5 (CH), 40.9 (CH_2), 35.5 (CH_2), 21.0 (4xCH_3), 13.0 (CH_3).
IR ν_{max} (neat)/cm^{-1}: 3437, 2967, 2932, 1669, 1292, 1048, 922, 772.
HRMS (ESI) calc'd for $C_{17}H_{31}NNaO_4$ [M+Na]$^+$: 336.2145; found: 336.2155.
[α]$_D$^{22}= +22.5 (*c* 1.1, $CHCl_3$).

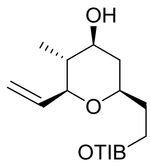

12b

Compound **12b** was obtained as a colourless oil in 71% yield (96:4 *dr*) (611 mg, 1.47 mmol) by flash column chromatography (SiO_2, petroleum ether: EtOAc, 8:2) from allyl boronic ester (*R*)-**6** (422 mg, 2.32 mmol), *n*-BuLi (1.59 mL, 2.55 mmol), TFAA (0.39 mL, 2.8 mmol), **7b** (1.06 g, 3.48 mmol), acrolein (0.62 mL, 9.3 mmol) and K_2CO_3 (480 mg, 3.48 mmol).
R$_f$ = 0.28 (petroleum ether: EtOAc, 8:2).
^{1}H NMR (400 MHz, $CDCl_3$) δ (ppm) 6.99 (s, 2H), 5.78 (ddd, *J* = 17.4, 10.4, 7.2 Hz, 1H), 5.24 (dd, *J* = 17.2, 10.4 Hz, 2H), 4.45 – 4.38 (m, 2H), 3.61 – 3.52 (m, 1H), 3.40 – 3.29 (m, 2H), 2.93 – 2.74 (m, 3H), 2.04 – 1.81 (m, 4H), 1.39 (app q, 1H), 1.23 (d, *J* = 6.9 Hz, 18H), 0.94 (d, *J* = 6.5 Hz, 3H).
^{13}C NMR (100 MHz, $CDCl_3$) δ (ppm) 171.1 (C), 150.2 (C), 144.8 (2xC), 136.8 (CH), 130.6 (C), 120.9 (2xCH), 117.8 (CH_2), 83.0 (CH), 73.6 (CH), 72.0 (CH), 61.7 (CH_2), 43.6 (CH), 41.0 (CH_2), 35.0 (CH_2), 34.5 (CH), 31.6 (2xCH), 24.2 (4xCH_3), 24.0 (2xCH_3), 13.1 (CH_3).
IR ν_{max} (neat)/cm^{-1}: 3437, 2961, 1724, 1606, 1462, 1363, 1251, 1074, 922, 877, 732.
HRMS (ESI) calc'd for $C_{26}H_{40}NaO_4$ [M+Na]$^+$ 439.2819; found: 439.2825.
[α]$_D$^{22}= +21.1 (*c* 1.6, $CHCl_3$).

Glycosylation reaction

- Synthesis of trichloroacetimidate derivative **13**

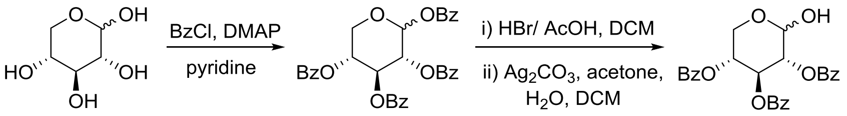

99% **20** **90%** **21**

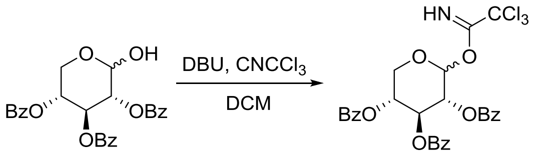

21 **87%** **13**

Benzoyl chloride (5.62 g, 40 mmol, 6 equiv.) was slowly added to a solution of xylose (1.00 g, 6.67 mmol, 1 equiv.) and DMAP (81 mg, 0.66 mmol, 0.1 equiv.) in pyridine (13 mL) at 0 °C. The reaction was stirred for 16 h at room temperature and was quenched with MeOH (15 mL). The mixture was diluted with DCM and washed with 2N HCl (x2), saturated aq. $NaHCO_3$ (x2) and

brine (x1). The organic layer was dried with $MgSO_4$, filtered and evaporated under reduced pressure. The residue was purified by flash column chromatography (SiO_2, petroleum ether: EtOAc, 6:4) to afford compound **20** as a white solid in 99% yield (3.74 g, 6.60 mmol).

R_f = 0.37 (petroleum ether: EtOAc, 6:4).

Data of the major isomer are reported.

^{1}H NMR (500 MHz, $CDCl_3$) δ (ppm) 8.15 (dd, *J* = 8.2, 1.1 Hz, 2H), 7.99 (dd, *J* = 8.3, 1.2 Hz, 2H), 7.95 (dd, *J* = 8.2, 1.1 Hz, 2H), 7.89 (dd, *J* = 8.3, 1.2 Hz, 2H), 7.67 – 7.60 (m, 1H), 7.56 – 7.49 (m, 3H), 7.49 – 7.42 (m, 2H), 7.42 – 7.36 (m, 2H), 7.36 – 7.26 (m, 4H), 6.77 (d, *J* = 3.6 Hz, 1H), 6.28 (t, *J* = 9.8 Hz, 1H), 5.64 (dd, *J* = 10.1, 3.7 Hz, 1H), 5.55 (td, *J* = 10.3, 5.7 Hz, 1H), 4.30 (dd, *J* = 11.2, 5.7 Hz, 1H), 4.05 (t, *J* = 10.9 Hz, 1H).

^{13}C NMR (125 MHz, $CDCl_3$) δ (ppm) 165.9 (C), 165.5 (C), 165.4 (C), 164.6 (C), 133.94 (CH), 133.6 (CH), 133.5 (CH), 133.4 (CH), 130.0 (2xCH), 129.9 (2xCH), 129.84 (2xCH), 129.76 (2xCH), 129.1 (C), 129.0 (C), 128.9 (C), 128.8 (2xCH), 128.6 (C), 128.5 (2xCH), 128.4 (4xCH), 90.3 (CH), 70.3 (CH), 70.0 (CH), 69.5 (CH), 61.3 (CH_2).

$[\alpha]_D^{22}$= +133 (*c* 1, $CHCl_3$).

The spectroscopic data are in accordance with the literature.[11]

Hydrogen bromide in acetic acid (33%, 3.3 mL) was added under N_2 to a solution of compound **20** (2.64 g, 4.65 mmol, 1 equiv.) in dry DCM (14 mL). After 2.5 h of stirring at room temperature, the reaction was quenched with sat. aq. $NaHCO_3$. The organic phase was washed with sat. aq. $NaHCO_3$ (x2) and brine (x1), dried over $MgSO_4$ and evaporated under reduced pressure. The crude reaction mixture was re-dissolved in acetone (24 mL), water (1 mL) and DCM (2.2 mL) and Ag_2CO_3 (2.23 g, 8.08 mmol, 1.75 equiv.) was added portionwise. The reaction was stirred for 1 h at room temperature and then filtered through celite and anhydrous $MgSO_4$. The filtrate was evaporated under reduced pressure and purified by flash column chromatography (SiO_2, petroleum ether: EtOAc, 7:3). The corresponding compound **21** was obtained in 90% yield (1.93 g, 4.18 mmol) as a white solid.

R_f: 0.37 (petroleum ether: EtOAc, 7:3).

Data of the major isomer are reported:

^{1}H NMR (500 MHz, $CDCl_3$) δ (ppm) 8.03 (dd, *J* = 8.4, 1.4 Hz, 2H), 8.00 (dd, *J* = 8.3, 1.3 Hz, 2H), 7.96 (dd, *J* = 8.4, 1.3 Hz, 2H), 7.58 – 7.51 (m, 2H), 7.51 – 7.46 (m, 1H), 7.45 – 7.37 (m, 4H), 7.38 – 7.33 (m, 2H), 6.22 (t, *J* = 9.5 Hz, 1H), 5.70 (d, *J* = 3.4 Hz, 1H), 5.43 (td, *J* = 9.0, 6.8 Hz, 1H), 5.31 (dd, *J* = 9.8, 3.5 Hz, 1H), 4.16 (d, *J* = 2.3 Hz, 1H), 4.14 (t, *J* = 7.3 Hz, 1H), 3.12 (br. s, 1H).

^{13}C NMR (125 MHz, $CDCl_3$) δ (ppm) 165.9 (C), 165.8 (C), 165.6 (C), 133.5 (CH), 133.4 (CH), 133.2 (CH), 129.9 (2xCH), 129.8 (2xCH), 129.7 (2xCH), 129.3 (C), 129.1 (C), 129.0(C), 128.48 (2xCH), 128.45 (2xCH), 128.4 (2xCH), 90.7 (CH), 72.0 (CH), 69.9 (CH), 69.5 (CH), 59.0 (CH_2).

$[\alpha]_D^{22}$= +27 (*c* 1, $CHCl_3$).

[11] F. W. Lichtenthaler, H. J. Lindner, *Carbohydr. Res.* **1990**, *200*, 91.

The spectroscopic data are in accordance with the literature.[12]

Hemiacetal **21** (1.60 g, 3.46 mmol, 1 equiv.) was dissolved in dry DCM (12.8 mL) under N_2. Trichloroacetonitrile (2.8 mL, 28 mmol, 8 equiv.) was added and the mixture was cooled to 0 °C. 1,8-Diazabicyclo[5.4.0]undec-7-ene (DBU) (0.52 mL, 3.5 mmol, 1 equiv.) was then added and the reaction was stirred for 1 h at 0 °C and for a further 3 h at room temperature. The solvent was evaporated under reduced pressure and the residue was purified by flash column chromatography (SiO_2, petroleum ether: AcOEt = 2:1 containing 0.1% of Et_3N). The corresponding trichloroacetimidate **13** was obtained in 87% yield (1.82 g, 3.01 mmol) as a yellow foamy solid.

Data of the major isomer are reported:

R*f* = 0.56 (petroleum ether: EtOAc, 2:1).

^{1}H NMR (500 MHz, $CDCl_3$) δ (ppm) 8.66 (s, 1H), 8.05 – 7.93 (m, 6H), 7.64 – 7.32 (m, 9H), 6.76 (d, *J* = 3.6 Hz, 1H), 6.27 (t, *J* = 9.9 Hz, 1H), 5.59 (dd, *J* = 10.1, 3.7 Hz, 1H), 5.57 – 5.48 (m, 2H), 4.33 (dd, *J* = 11.2, 5.8 Hz, 1H), 4.08 (t, *J* = 11.0 Hz, 1H).

^{13}C NMR (125 MHz, $CDCl_3$) δ (ppm) 165.7 (C), 165.6 (C), 165.4 (C), 160.8 (C), 133.6 (2xCH), 133.3 (CH), 129.92 (2xCH), 129.90 (2xCH), 129.7 (2xCH), 129.0 (C), 128.8 (C), 128.6 (C), 128.5 (2xCH), 128.44 (2xCH), 128.39 (2xCH), 93.5 (CH), 70.6 (CH), 69.7 (CH), 69.4 (CH), 61.3 (CH_2).

$[\alpha]_D^{22}$= +40 (*c* 1, $CHCl_3$).

The spectroscopic data are in accordance with the literature.[13]

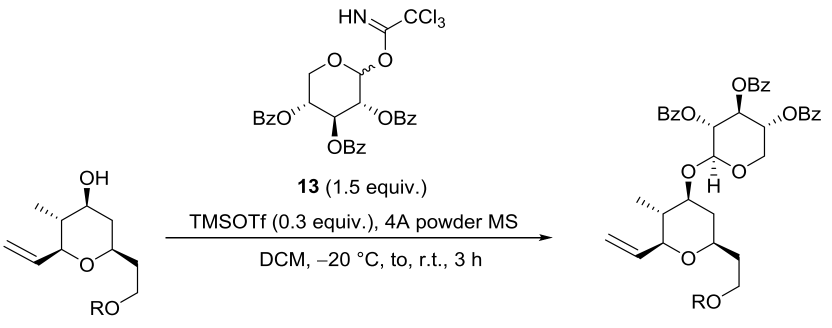

12a, R= Cb
12b, R= TIB

22a, R= Cb, **95%, *d.r.*>99:1**
22b, R= TIB, **92%, *d.r.*>99:1**

A mixture of **12a/12b** (1 equiv.), **13** (1.5 equiv.) and 4 Å powder molecular sieves (0.5n equiv. g) were dissolved in DCM (0.05 M) and stirred under N_2 for 30 min at room temperature. TMSOTf (0.3 equiv.) was added at −20 °C. The reaction was stirred at this temperature for 30 min and allowed to slowly reach room temperature over 2 h. The reaction was quenched upon addition of Et_3N (3.3 equiv.), the solution was filtered through celite and the solvent was removed under reduced pressure. The crude reaction mixture was purified by flash column chromatography.

[12] J. F. Batey, C. Bullock, E. O'Brien, J. M. Williams, *Carbohydr. Res.* **1975**, *43*, 43.
[13] L. Chen, F. Kong, *Carbohydr. Res.* **2002**, *337*, 2335.

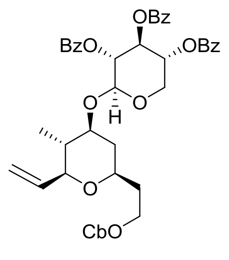

22a

Compound **22a** was obtained as a white foam in 95% yield (1.84 g, 2.43 mmol) by flash column chromatography (SiO_2, petrol ether: Et_2O, 6:4) from **12a** (802 mg, 2.56 mmol), **13** (2.32 g, 3.83 mmol), 4 Å powder molecular sieves (400 mg) and TMSOTf (0.14 mL, 0.77 mmol).

R$_f$ = 0.37 (petroleum ether: Et_2O, 4:6).

^{1}H NMR (500 MHz, CDCl$_3$) δ (ppm) 7.92 (d, *J* = 8.3 Hz, 2H), 7.88 (d, *J* = 8.3 Hz, 4H), 7.51 – 7.37 (m, 3H), 7.33 – 7.23 (m, 6H), 5.71 (t, *J* = 7.8 Hz, 1H), 5.62 (ddd, *J* = 17.4, 10.4, 7.2 Hz, 1H), 5.35 (dd, *J* = 7.9, 6.0 Hz, 1H), 5.24 (td, *J* = 7.6, 4.5 Hz, 1H), 5.17 – 5.03 (m, 2H), 4.84 (d, *J* = 5.9 Hz, 1H), 4.37 (dd, *J* = 12.1, 4.5 Hz, 1H), 4.18 – 4.05 (m, 2H), 3.60 (dd, *J* = 12.1, 7.7 Hz, 1H), 3.48 – 3.36 (m, 1H), 3.31-3.24 (m, 2H), 2.11 (ddd, *J* = 12.7, 4.7, 1.8 Hz, 1H), 1.90 – 1.73 (m, 2H), 1.47 (app q, 1H), 1.36 – 1.27 (m, 1H), 1.12 (d, *J* = 6.8 Hz, 12H), 0.66 (d, *J* = 6.5 Hz, 3H). Signals corresponding to N(C**H**CH$_3$)$_2$ are not observed.

^{13}C NMR (125 MHz, CDCl$_3$) δ (ppm) 165.5 (2xC), 165.1 (C), 155.8 (C), 136.7 (CH), 133.40 (CH), 133.35 (CH), 133.3 (CH), 129.9 (2xCH), 129.83 (2xCH), 129.78 (2xCH), 129.3 (C), 129.2 (C), 129.1 (C), 128.5 (2xCH), 128.4 (2xCH), 128.3 (2xCH), 117.8 (CH_2), 102.0 (CH), 83.9 (CH), 82.9 (CH), 72.4 (CH), 70.9 (CH), 70.8 (CH), 69.4 (CH), 61.7 (CH_2), 61.5 (CH_2), 45.4 (2xCH), 41.5 (CH), 39.9 (CH_2), 35.5 (CH_2), 21.0 (4xCH_3), 13.0 (CH_3).

HRMS (ESI) calc´d for $C_{43}H_{51}NNaO_{11}$ $[M+Na]^+$: 780.3354; found: 780.3368.

IR ν_{max} (neat)/cm^{-1}: 2976, 1724, 1681, 1314, 1279, 1257, 1090, 1067, 1044, 1026, 910, 707.

$[\alpha]_D^{22}$ = +1.38 (*c* 1.4, $CHCl_3$).

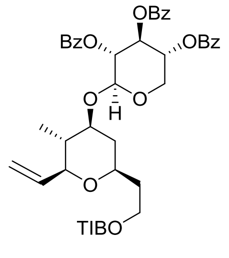

22b

Compound **22b** was obtained as a white foam in 93% yield (1.06 g, 1.23 mmol) by flash column chromatography (SiO_2, petroleum ether: EtOAc, 9:1) from **12b** (550 mg, 1.32 mmol), **13** (1.19 g ,1.98 mmol), 4 Å powder molecular sieves (275 mg) and TMSOTf (72 µL, 0.40 mmol).

R$_f$ = 0.55 (petroleum ether: EtOAc, 8:2).

^{1}H NMR (500 MHz, CDCl$_3$) δ (ppm) 8.04 – 7.94 (m, 6H), 7.60 – 7.47 (m, 3H), 7.45 – 7.33 (m, 6H), 7.03 (s, 2H), 5.82 (t, *J* = 8.0 Hz, 1H), 5.73 (ddd, *J* = 17.4, 10.4, 7.2 Hz, 1H), 5.45 (dd, *J* = 8.1, 6.2 Hz, 1H), 5.24 (td, *J* = 7.9, 4.7 Hz, 1H), 5.21 (dd, *J* = 17.4, 10.4 Hz, 2H), 4.91 (d, *J* = 6.2 Hz, 1H), 4.48 – 4.48 (m, 3H), 3.67 (dd, *J* = 12.0, 7.9 Hz, 1H), 3.63 – 3.54 (m, 1H), 3.41 – 3.29 (m, 2H), 2.94 – 2.82 (m, 3H), 2.19 (dd, *J* = 12.7, 4.8 Hz, 1H), 2.07 – 1.89 (m, 2H), 1.64 – 1.53 (m, 1H), 1.49 – 1.34 (m, 1H), 1.27 (d, *J* = 7.0 Hz, 18H), 0.75 (d, *J* = 6.5 Hz, 3H).

^{13}C NMR (125 MHz, CDCl$_3$) δ (ppm) 171.4 (C) , 166.1 (2xC), 165.70 (C), 150.76 (C) , 145.31 (2xC), 137.26 (CH), 134.1 (CH), 134.0 (CH), 133.9 (CH), 131.2 (C), 130.54 (2xCH), 130.49 (2xCH), 130.45 (2xCH), 129.9 (C), 129.8 (C), 129.75 (C), 129.1 (2xCH), 129.04 (2xCH), 129.01 (2xCH), 121.5 (2xCH), 118.6 (CH_2), 103.0 (CH), 84.6 (CH), 83.7 (CH), 72.5 (CH), 71.7 (2xCH), 70.2 (CH), 62.6 (CH_2), 62.4 (CH_2), 42.3 (CH), 40.7 (CH_2), 35.8 (CH_2), 35.3 (CH), 32.3 (2xCH), 25.1 (4xCH_3), 24.8 (2xCH_3), 13.8 (CH_3).

HRMS (ESI) calc´d for $C_{52}H_{60}NaO_{11}$ $[M+Na]^+$: 883.4028; found: 883.4027.

IR ν_{max} (neat)/cm^{-1}: 2976, 1724, 1262, 1095, 712.
[α]$_D$^{22}= +3.27 (*c* 0.9, $CHCl_3$).

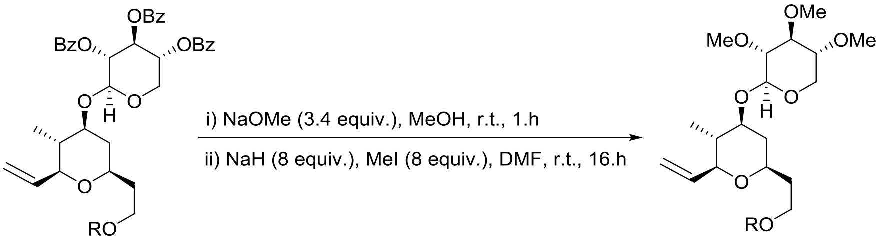

22a, R= Cb
22b, R= TIB

3a, R= Cb, **93%**,
3b, R= TIB, **93%**

To a stirred solution of **22a** or **22b** (1 equiv.) in dry MeOH (0.09M) was added NaOMe (3.4 equiv.) and the reaction was stirred for 1 h at room temperature. The reaction mixture was acidified (pH=5) with Amberlite (H^+) IR-120 resin. After filtering the resin, the solvent was evaporated and the crude reaction mixture was dissolved in dry DMF (0.07 M) under N_2. NaH (60% dispersion in mineral oil, 8 equiv.) was added at 0 °C and the reaction was stirred 30 min at this temperature and for a further 30 min at room temperature. MeI (8 equiv.) was added and the reaction was stirred overnight. H_2O was added, the aqueous phase was extracted with EtOAc (x3) and the combined organic layers were washed with brine (x2), dried over $MgSO_4$, filtered and evaporated under reduced pressure. The crude reaction mixture was purified by flash column chromatography.

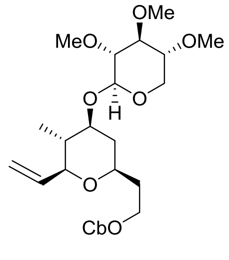

3a

Compound **3a** was obtained as a viscous oil in 93% yield (1.08 g, 2.21 mmol) by flash column chromatography (SiO_2, petroleum ether: EtOAc, 7:3) from **22a** (1.80 g, 2.38 mmol), NaOMe (437 mg, 8.09 mmol), NaH (761 mg, 19.0 mmol) and MeI (1.18 mL, 19.0 mmol).

R$_f$ = 0.49 (petroleum ether: EtOAc, 1:1).

^{1}H NMR (500 MHz, $CDCl_3$) δ (ppm) 5.79 (ddd, *J* = 17.4, 10.4, 7.1 Hz, 1H), 5.23 (dd, *J* = 17.4, 10.4 Hz, 2H), 4.31 (d, *J* = 7.6 Hz, 1H), 4.19 (td, *J* = 7.0, 7.0, 1.9 Hz, 2H), 3.97 (dd, *J* = 11.5, 5.2 Hz, 1H), 3.63 (s, 3H), 3.60 (s, 3H), 3.54 – 3.50 (m, 1H), 3.48 (s, 3H), 3.44 – 3.39 (m, 1H), 3.34 – 3.21 (m, 2H), 3.11 (td, *J* = 10.1, 9.5, 5.0 Hz, 2H), 2.99 (dd, *J* = 9.1, 7.6 Hz, 1H), 2.12 (dd, *J* = 12.7, 4.8, 1.8 Hz, 1H), 1.98 – 1.88 (m, 1H), 1.87 – 1.80 (m, 1H), 1.54 – 1.39 (m, 2H), 1.21 (d, *J* = 6.9 Hz, 12H), 0.94 (d, *J* = 6.5 Hz, 3H). Signals corresponding to N(C**H**CH_3)$_2$ are not observed.

^{13}C NMR (125 MHz, $CDCl_3$) δ (ppm) 155.8 (C), 136.8 (CH), 117.7 (CH_2), 105.4 (CH), 85.5 (CH) 83.8 (CH), 83.2 (CH), 83.0 (CH), 79.4 (CH), 72.5 (CH), 63.2 (CH_2), 61.5 (CH_2), 60.79 (CH_3), 60.76 (CH_3), 58.8 (CH_3), 45.6 (2xCH), 42.0 (CH), 40.2 (CH_2), 35.4 (CH_2), 21.0 (4xCH_3), 13.0 (CH_3).

HRMS (ESI) calc´d for $C_{25}H_{45}NNaO_8$ $[M+Na]^+$: 510.3037; found: 510.3026.

IR ν_{max} (neat)/cm^{-1}: 2973, 2930, 1688, 1089, 1075, 771.

[α]$_D^{22}$= +1.31 (*c* 0.8, $CHCl_3$).

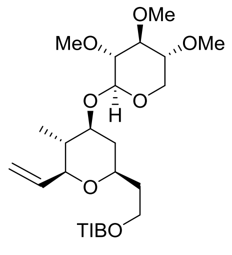

3b

Compound **3b** was obtained as a viscous oil in 93% yield (638 mg, 1.08 mmol) by flash column chromatography (SiO_2, petroleum ether: EtOAc, 8:2) from **22b** (1.00 g, 1.16 mmol), NaOMe (213 mg, 3.95 mmol), NaH (372 mg, 9.30 mmol) and MeI (0.58 mL, 9.3 mmol).

R_f = 0.59 (petroleum ether: EtOAc, 7:3).

^{1}H NMR (500 MHz, $CDCl_3$) δ (ppm) 7.02 (s, 2H), 5.81 (ddd, *J* = 17.4, 10.4, 7.1 Hz, 1H), 5.24 (dd, *J* = 17.4, 10.4 Hz, 2H), 4.49 – 4.39 (m, 2H), 4.29 (d, *J* = 7.6 Hz, 1H), 3.96 (dd, *J* = 11.5, 5.3 Hz, 1H), 3.63 (s, 3H), 3.60 (s, 3H), 3.59 – 3.54 (m, 1H), 3.48 (s, 3H), 3.41 (app t, 1H), 3.31 – 3.22 (m, 2H), 3.14 – 3.05 (m, 2H), 2.98 (app t, 1H), 2.93 – 2.81 (m, 3H), 2.11 (dd, *J* = 12.7, 4.8 Hz, 1H), 2.03 – 1.95 (m, 1H), 1.94 – 1.86 (m, 1H), 1.56 – 1.42 (m, 2H), 1.26 (d, *J* = 6.9 Hz, 18H), 0.99 (d, *J* = 6.5 Hz, 3H).

^{13}C NMR (125 MHz, $CDCl_3$) δ (ppm) δ 171.4 (C), 150.7 (C), 145.3 (2xC), 137.4 (CH), 131.3 (C), 121.5 (2xCH), 118.5 (CH_2), 106.3 (CH), 86.3 (CH), 84.6 (CH), 84.0 (CH), 83.8 (CH), 80.1 (CH), 72.6 (CH), 64.0 (CH_2), 62.4 (CH_2), 61.6 (2xCH_3), 59.6 (CH_3), 42.8 (CH), 41.0 (CH_2), 35.7 (CH_2), 35.3 (CH), 32.3 (2xCH_2), 25.0 (4xCH_3), 24.8 (2xCH_3), 13.9 (CH_3).

HRMS (ESI) calc'd for $C_{34}H_{54}NaO_8$ [M+Na]$^+$: 613.3711; found: 613.3713.

IR ν_{max} (neat)/cm^{-1}: 2962, 1725, 1252, 1165, 1075, 1075, 988, 876.

[α]$_D^{22}$= +1.48 (*c* 0.7, $CHCl_3$).

Hydroboration-oxidation-protection

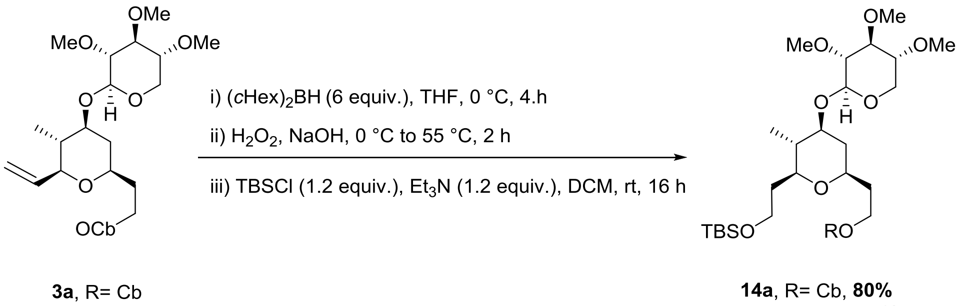

A solution (*c*Hex)$_2$BH was freshly prepared upon addition of cyclohexene (12 equiv.) to BH_3•SMe_2 (6 equiv.) in dry THF (1 M) at room temperature under N_2. To the prepared solution was added dropwise **14a** or **14b** (1 equiv.) as a solution in THF (0.07 M) at 0 °C under N_2. The mixture was stirred for 4 h at 0 °C. At this temperature, dry MeOH (12 equiv.), 3N NaOH and H_2O_2 (30%) were added sequentially. The reaction was then slowly warmed to 55 °C and stirred for 1 h. The reaction was cooled to room temperature and extracted with EtOAc (x3). The combined organic layers were washed with water (x1), brine (x1), dried over $MgSO_4$, filtered and evaporated under reduced pressure. The crude reaction mixture was dissolved in DCM (0.1 M) and Et_3N (1.2 equiv.)

and TBSCl (1.2 equiv.) were added. The mixture was stirred overnight at room temperature. The reaction was washed with 10% sat aq. $NaHCO_3$ (x2), water (x1) and brine (x1), dried over $MgSO_4$ and evaporated under reduced pressure. The crude reaction mixture was purified by flash column chromatography.

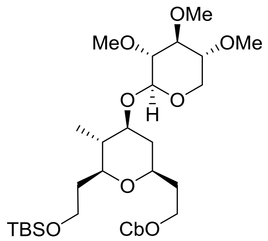

14a

Compound **14a** was obtained as a viscous oil in 80% yield (711 mg, 1.15 mmol) by flash column chromatography (SiO_2, petroleum ether: EtOAc, 8:2) from **3a** (700 mg, 1.44 mmol), (*c*Hex)$_2$BH (8.63 mmol), 3N NaOH (4.3 mL), H_2O_2 (30%) (3.4 mL), Et_3N (0.24 mL, 1.7 mmol) and TBSCl (260 mg, 1.72 mmol).

R$_f$ = 0.45 (petroleum ether: EtOAc, 6:4).

^{1}H NMR (500 MHz, CDCl$_3$) δ (ppm) 4.30 (d, *J* = 7.6 Hz, 1H), 4.24 – 4.11 (m, 2H), 3.96 (dd, *J* = 11.6, 5.2 Hz, 1H), 3.78 – 3.71 (m, 2H), 3.63 (s, 3H), 3.60 (s, 3H), 3.48 (s, 3H), 3.45 – 3.38 (m, 1H), 3.29 – 3.22 (m, 2H), 3.14 – 3.06 (m, 3H), 2.98 (dd, *J* = 9.1, 7.6 Hz, 1H), 2.07 (ddd, *J* = 12.6, 4.9, 1.8 Hz, 1H), 1.96 – 1.87 (m, 1H), 1.83 (ddd, *J* = 11.6, 9.7, 5.7 Hz, 2H) 1.56 (ddt, *J* = 14.3, 9.9, 5.1 Hz, 1H), 1.41 (dt, *J* = 13.6, 11.4 Hz, 2H), 1.21 (d, *J* = 6.8 Hz, 12H), 1.01 (d, *J* = 6.5 Hz, 3H), 0.90 (s, 9H), 0.05 (s, 6H). Signals corresponding to N(C**H**CH$_3$)$_2$ are not observed.

^{13}C NMR (125 MHz, CDCl$_3$) δ (ppm) 155.7 (C), 105.5 (CH), 85.6 (CH), 83.9 (CH), 83.5 (CH), 79.4 (CH), 77.8 (CH), 72.1 (CH), 63.2 (CH$_2$), 61.4 (CH$_2$), 60.79 (CH$_3$), 60.75 (CH$_3$), 59.6 (CH$_2$), 58.8 (CH$_3$), 45.5 (2xC), 42.3 (CH), 40.4 (CH$_2$), 36.3 (CH$_2$), 35.6 (CH$_2$), 26.0 (3xCH$_3$), 21.1 (4xCH$_3$), 18.3 (C), 12.8 (CH$_3$), -5.29 (CH$_3$), -5.33 (CH$_3$).

HRMS (ESI) calc'd for $C_{31}H_{61}NNaO_9Si$ $[M+Na]^+$ 642.4008; found: 642.4006.

IR ν_{max} (neat)/cm^{-1}: 2928, 1692, 1078, 834, 773.

[α]$_D$^{22}= -16.0 (*c* 1, CHCl$_3$).

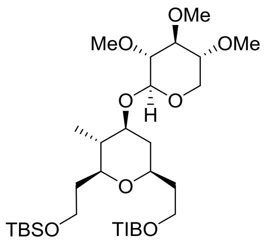

14b

Compound **14b** was obtained as a viscous oil in 80% yield (497 mg, 0.687 mmol) by flash column chromatography (SiO_2, petroleum ether: EtOAc, 9:1) from from **3b** (507 mg, 0.859 mmol), (*c*Hex)$_2$BH (5.16 mmol), 3N NaOH (2.6 mL), H_2O_2 (30%) (2.05 mL), Et_3N (0.14 mL, 1.0 mmol) and TBSCl (155 mg, 1.03 mmol).

R$_f$ = 0.37 (petroleum ether:EtOAc, 8:2).

^{1}H NMR (500 MHz, CDCl$_3$) δ (ppm) 7.02 (s, 2H), 4.41 (t, *J* = 6.6 Hz, 2H), 4.28 (d, *J* = 7.6 Hz, 1H), 3.97 (dd, *J* = 11.6, 5.2 Hz, 1H), 3.84 – 3.78 (m, 1H), 3.77 – 3.69 (m, 1H), 3.64 (s, 3H), 3.61 (s, 3H), 3.49 (s, 3H), 3.48 – 3.44 (m, 1H), 3.29 – 3.18 (m, 2H), 3.14 – 3.04 (m, 2H), 2.99 (app t, 1H), 2.94 – 2.88 (m, 1H), 2.88 – 2.81 (m, 2H), 2.10 (dd, *J* = 12.7, 4.8 Hz, 1H), 2.01 – 1.81 (m, 3H), 1.68 – 1.56 (m, 2H), 1.53 – 1.37 (m, 2H), 1.27 (d, *J* = 6.9 Hz, 18H), 1.02 (d, *J* = 6.5 Hz, 3H), 0.91 (s, 9H), 0.07 (s, 6H).

^{13}C NMR (125 MHz, CDCl$_3$) δ (ppm) δ 171.4 (C), 150.7 (C), 145.3 (2xC), 131.2 (C), 121.5 (2xCH), 106.4 (CH), 86.3 (CH), 84.6 (CH), 84.3 (CH), 80.1 (CH), 78.8 (CH), 72.4 (CH), 64.0 (CH$_2$), 62.5 (CH$_2$), 61.6 (2xCH$_3$), 60.6 (CH$_2$), 59.6 (CH$_3$), 43.1 (CH), 41.2 (CH$_2$), 37.1 (CH$_2$), 35.9 (CH$_2$), 35.3 (CH), 32.3

(2xCH), 26.8 (3xCH$_3$), 25.04 (2xCH$_3$), 24.99 (2xCH$_3$), 24.8 (2xCH$_3$), 19.2 (C), 13.7 (CH$_3$), -4.37 (CH$_3$), -4.44 (CH$_3$).

HRMS (ESI) calc´d for $C_{40}H_{70}NaO_9Si$ [M+Na]$^+$: 745.4681; found: 745.4686.

IR ν_{max} (neat)/cm^{-1}: 2958, 2928, 1726, 1462, 1251, 1090, 835, 775.

[α]$_D$^{22}= -10.3 (c 0.9, CHCl$_3$).

Lithiation-borylation reaction

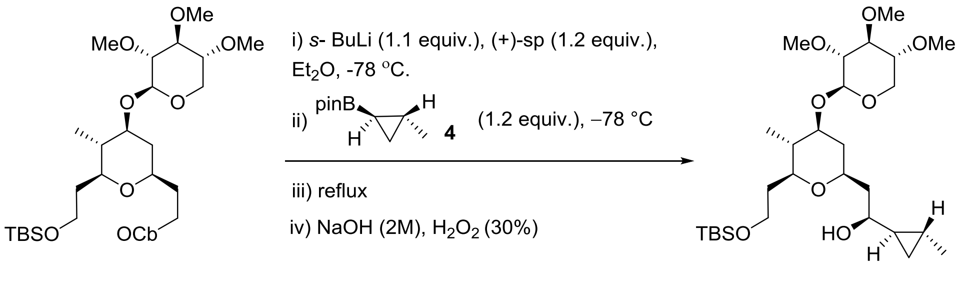

14a, R= Cb
14b, R= TIB

15a

From compound **14a**: To a solution of primary carbamate **14a** (80 mg, 0.13 mmol, 1 equiv.) and (+)-sparteine (36 mg, 0.16 mmol, 1.2 equiv.) in Et$_2$O (0.7 mL) at –78 °C under N$_2$ was added *s*-BuLi (1.3 M in 92:8 cyclohexane/hexane, 0.14 mmol, 1.1 equiv.) dropwise. The resulting yellow mixture was stirred for 5 h at –78 °C before boronic ester **4** (28 mg, 0.16 mmol, 1.2 equiv.) was added as a 1 M Et$_2$O solution. The reaction mixture was further stirred at –78 °C for 1 h, before being allowed to warm to room temperature. The reaction mixture was refluxed for 16 h. The reaction mixture was cooled to 0 °C and a solution of NaOH (2 M)/H$_2$O$_2$ (30%) (2:1 v/v, 0.54 mL) was added dropwise and allowed to stir at room temperature for 2 h. After this time the layers were separated and the aqueous phase was extracted with Et$_2$O (x3). The combined organic layers were dried over MgSO$_4$, filtered and evaporated under reduced pressure. The crude reaction mixture was purified by flash column chromatography (petroleum ether: EtOAc, 6:4) to give **15a** in 48% yield (34 mg, 0.06 mmol) as a pale yellow oil.

Note: Some problems on reproducibility were found when the reaction was repeated and/or the scale increased (23-48%).

From compound **14b**: To a solution of triisopropylbenzoate **14b** (218 mg, 0.301 mmol, 1 equiv.) and (+)-sparteine (84 mg, 0.36 mmol, 1.2 equiv.) in Et$_2$O (1.6 mL) at –78 °C under N$_2$ was added *s*-BuLi (1.3 M in 92:8 cyclohexane/hexane, 0.25 mL, 0.33 mmol, 1.1 equiv.) dropwise. The resulting yellow mixture was stirred for 1 h at –78 °C before boronic ester **4** (65 mg, 0.36 mmol, 1.2 equiv.) was added as a 1 M Et$_2$O solution. The reaction mixture was further stirred at –78 °C for 1 h, before being allowed to warm to room temperature and was refluxed for 2 h. The reaction mixture was cooled to 0 °C and a solution of NaOH (2 M)/H$_2$O$_2$ (30%) (2:1 v/v, 1.23 mL) was added dropwise and allowed to stir at room temperature for 2 h. After this time the layers

were separated and the aqueous phase was extracted with Et_2O (x3). The combined organic layers were dried over $MgSO_4$, filtered and evaporated under reduced pressure. The crude reaction mixture was purified by flash column chromatography (SiO_2, petroleum ether: EtOAc, 6:4) to give **15a** in 73% yield (120 mg, 0.22 mmol) as a pale yellow oil.

R_f = 0.37 (petroleum ether: EtOAc, 6:4).

^{1}H NMR (500 MHz, $CDCl_3$) δ (ppm) 4.26 (d, *J* = 7.6 Hz, 1H), 3.93 (dd, *J* = 11.5, 5.2 Hz, 1H), 3.72 – 3.62 (m, 2H), 3.60 (s, 3H), 3.58 (s, 3H), 3.58 – 3.48 (m, 1H), 3.46 (s, 3H), 3.27 – 3.14 (m, 3H), 3.12 – 3.04 (m, 3H), 2.96 (dd, *J* = 9.1, 7.6 Hz, 1H), 2.06 (ddd, *J* = 12.7, 4.7, 1.6 Hz, 1H), 1.87 (dddd, *J* = 14.2, 8.9, 6.7, 2.4 Hz, 1H), 1.80 – 1.72 (m, 1H), 1.73 – 1.66 (m, 2H), 1.53 (*J* = 14.3, 9.7, 4.9 Hz, 1H), 1.47 – 1.36 (m, 1H), 1.04 (d, *J* = 6.0 Hz, 3H), 0.99 (d, *J* = 6.5 Hz, 3H), 0.87 (s, *J* = 3.2 Hz, 9H), 0.78 – 0.67 (m, 1H), 0.57 (dt, *J* = 8.6, 4.6 Hz, 1H), 0.29 (dt, *J* = 8.4, 4.7 Hz, 1H), 0.16 (dt, *J* = 8.8, 4.8 Hz, 1H), 0.03 (s, 6H).

^{13}C NMR (125 MHz, $CDCl_3$) δ (ppm) 105.6 (CH), 85.5 (CH), 83.8 (CH), 83.2 (CH), 79.4 (CH), 78.1 (CH), 75.9 (CH), 75.8 (CH), 63.2 (CH_2), 60.77 (CH_3), 60.75 (CH_3), 59.3 (CH_2), 58.8 (CH_3), 42.6 (CH_2), 42.0 (CH), 40.7 (CH_2), 35.9 (CH_2), 26.1 (CH), 25.9 (3xCH_3), 18.7 (CH_3), 18.2 (C), 12.8 (CH_3), 11.1 (CH), 10.2 (CH_2), -5.3 (CH_3), -5.4 (CH_3).

HRMS (ESI) calc´d for $C_{28}H_{54}NNaO_8Si$ $[M+Na]^+$: 569.3480; found: 569.3487.

IR ν_{max} (neat)/cm^{-1}: 2929, 1089, 1078, 729.

$[\alpha]_D^{22}$= -18.0 (*c* 1, $CHCl_3$).

*General procedure for the synthesis of diasteroisomers of **15a***

To a solution of triisopropylbenzoate **14a** (50 mg, 0.069 mmol, 1 equiv.) and chiral diamine (+) or (–)-sp (20 µL, 0.083 mmol, 1.2 equiv.) in Et_2O (0.5 mL) at –78 °C under N_2 was added *s*-BuLi (1.3 M in 92:8 cyclohexane/hexane, 61 µL, 0.076 mmol, 1.1 equiv.) dropwise. The resulting yellow mixture was stirred for 1 h at –78 °C and boronic ester **4** or *ent*-**4** (15 mg, 0.083 mmol, 1.2 equiv) was added as a 1 M Et_2O solution. The reaction mixture was stirred at –78 °C for a further 1 h, before being allowed to warm to room temperature and refluxed for 2 h. The reaction mixture was cooled to 0 °C and a solution of NaOH (2 M)/H_2O_2 (30%) (2:1 v/v, 0.34 mL) was added dropwise and the mixture was stirred at room temperature for 2 h. After this time the layers were separated and the aqueous phase was extracted with Et_2O (x3). The combined organic layers were dried over $MgSO_4$, filtered and evaporated under reduced pressure. The crude reaction mixture was purified by flash chromatography (SiO_2, petroleum ether: EtOAc, 6:4).

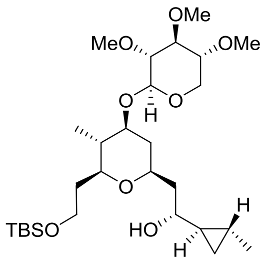

15b

For the synthesis of compound **15b**, (–)-sp and boronic ester **4** were used. Compound **15b** was obtained in 79% yield (30 mg, 0.054 mmol) as a colourless oil.

R$_f$ = 0.37 (petroleum ether: EtOAc, 6:4).

^{1}H NMR (500 MHz, CDCl$_3$) δ (ppm) 4.30 (d, *J* = 7.5 Hz, 1H), 3.97 (dd, *J* = 11.6, 5.4 Hz, 1H), 3.79 – 3.69 (m, 2H), 3.63 (s, 3H), 3.61 (s, 3H), 3.48 (s, 3H), 3.33 – 3.06 (m, 7H), 2.99 (dd, *J* = 9.2, 7.5 Hz, 1H), 2.69 (br. s, 1H), 2.04 (ddd, *J* = 12.7, 5.0, 1.8 Hz, 1H), 1.93 (dtd, *J* = 15.6, 7.8, 2.2 Hz, 1H), 1.78 (app t, 2H), 1.61 – 1.50 (m, 2H), 1.48 – 1.39 (m, 1H), 1.04 (d, *J* = 5.9 Hz, 3H), 1.02 (d, *J* = 6.6 Hz, 3H), 0.90 (s, 9H), 0.69 – 0.55 (m, 2H), 0.52 (dt, *J* = 8.6, 4.6 Hz, 1H), 0.29 (dt, *J* = 9.3, 4.7 Hz, 1H), 0.06 (s, 6H).

^{13}C NMR (125 MHz, CDCl$_3$) δ (ppm) 106.4 (CH), 86.3 (CH), 84.6 (CH), 84.3 (CH), 80.1 (CH), 78.7 (CH), 73.8 (CH), 73.4 (CH), 64.0 (CH_2), 61.6 (2xCH_3), 60.2 (CH_2), 59.6 (CH_3), 43.1 (CH), 42.9 (CH_2), 40.8 (CH_2), 36.9 (CH2), 27.0 (CH), 26.8 (3xCH_3), 19.3 (CH_3), 19.1 (C), 13.6 (CH_3), 12.4 (CH_2), 11.1 (CH), -4.4 (CH_3), -4.5 (CH_3).

HRMS (ESI) calc´d for $C_{28}H_{54}NNaO_8Si$ [M+Na]$^+$: 569.3480; found: 569.3468.

IR ν_{max} (neat)/cm^{-1}: 3501, 2928, 1081, 835, 775.

[α]$_D$^{22}= -21.1 (*c* 0.9, CHCl$_3$).

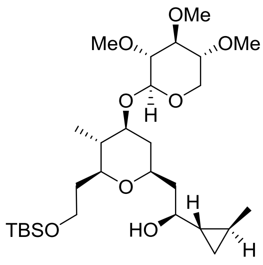

15c

For the synthesis of compound **15c**, (+)-sp and boronic ester ***ent*-4** were used. Compound **15c** was obtained in 76% yield (29 mg, 0.053 mmol) as a colourless oil.

R$_f$ = 0.37 (petroleum ether: EtOAc, 6:4).

^{1}H NMR (500 MHz, CDCl$_3$) δ (ppm) 4.29 (d, *J* = 7.6 Hz, 1H), 3.97 (dd, *J* = 11.6, 5.2 Hz, 1H), 3.76 – 3.65 (m, 2H), 3.63 (s, 3H), 3.61 (s, 3H), 3.59 – 3.52 (m, 1H), 3.48 (s, 3H), 3.29 – 3.22 (m, 2H), 3.19 (ddd, *J* = 11.0, 9.3, 2.3 Hz, 1H), 3.15 – 3.07 (m, 3H), 2.99 (dd, *J* = 9.1, 7.6 Hz, 1H), 2.10 (ddd, *J* = 12.8, 4.9, 1.8 Hz, 1H), 1.91 (dddd, *J* = 14.2, 8.8, 6.9, 2.3 Hz, 1H), 1.86 – 1.78 (m, 1H), 1.70 (dt, *J* = 14.4, 3.3 Hz, 1H), 1.59 (ddq, *J* = 14.1, 9.6, 5.4, 4.9 Hz, 1H), 1.52 – 1.41 (m, 2H), 1.03 (app t, *J* = 7.2 Hz, 6H), 0.90 (s, 9H), 0.63 – 0.56 (m, 2H), 0.55 – 0.47 (m, 1H), 0.34 – 0.25 (m, 1H), 0.06 (s, 6H).

^{13}C NMR (125 MHz, CDCl$_3$) δ (ppm) 105.6 (CH), 85.6 (CH), 83.8 (CH), 83.2 (CH), 79.4 (CH), 78.2 (CH), 75.6 (CH), 75.4 (CH), 63.2 (CH_2), 60.80 (CH_3), 60.78 (CH_3), 59.4 (CH_2), 58.8 (CH_3), 43.0 (CH_2), 42.0 (CH), 40.7 (CH_2), 36.0 (CH_2), 26.3 (CH), 25.9 (3xCH_3), 18.5 (CH_3), 18.2 (C) , 12.8 (CH_3), 11.3 (CH_2), 10.1 (CH), -5.3 (CH_3), -5.4 (CH_3).

HRMS (ESI) calc´d for $C_{28}H_{54}NNaO_8Si$ [M+Na]$^+$: 569.3480; found: 569.3474.

IR ν_{max} (neat)/cm^{-1}: 3499, 2952, 1078, 762.

[α]$_D$^{22}= -4.9 (*c* 1, CHCl$_3$).

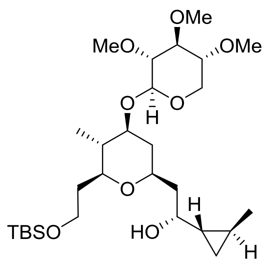

15d

For the synthesis of compound **15d**, (–)-sp and boronic ester ***ent*-4** were used. Compound **15d** was obtained in 75% yield (29 mg, 0.053 mmol) as a colourless oil.

R_f = 0.37 (petroleum ether: EtOAc, 6:4).

^{1}H NMR (500 MHz, CDCl$_3$) δ (ppm) 4.30 (d, *J* = 7.6 Hz, 1H), 3.97 (dd, *J* = 11.6, 5.2 Hz, 1H), 3.77 – 3.70 (m, 2H), 3.63 (s, 3H), 3.61 (s, 3H), 3.49 (s, 3H), 3.32 – 3.07 (m, 7H), 2.99 (dd, *J* = 9.1, 7.6 Hz, 1H), 2.85 (br. s, 1H), 2.03 (dd, *J* = 12.7, 4.9 Hz, 1H), 1.97 – 1.89 (m, 1H), 1.78 – 1.74 (m, 2H), 1.59 – 1.51 (m, 2H), 1.47 – 1.39 (m, 1H), 1.08 (d, *J* = 6.0 Hz, 3H), 1.02 (d, *J* = 6.5 Hz, 3H), 0.90 (s, 9H), 0.82 – 0.71 (m, 1H), 0.61 (tt, *J* = 8.7, 4.8 Hz, 1H), 0.32 (dt, *J* = 8.9, 4.7 Hz, 1H), 0.20 (dt, *J* = 9.1, 4.8 Hz, 1H), 0.06 (s, 6H).

^{13}C NMR (125 MHz, CDCl$_3$) δ (ppm) 106.4 (CH), 86.3 (CH), 84.6 (CH), 84.3 (CH), 80.1 (CH), 78.8 (CH), 73.9 (CH), 73.8 (CH), 64.0 (CH$_2$), 61.58 (CH$_3$), 61.57 (CH$_3$), 60.2 (CH$_2$), 59.6 (CH$_3$), 43.0 (CH), 42.3 (CH$_2$), 40.8 (CH$_2$), 36.9 (CH$_2$), 26.8 (3xCH$_3$), 26.7 (CH), 19.6 (CH$_3$), 19.1 (C), 13.6 (CH$_3$), 12.1 (CH), 11.1 (CH$_2$), -4.4 (CH$_3$), -4.5 (CH$_3$).

HRMS (ESI) calc´d for $C_{28}H_{54}NNaO_8Si$ [M+Na]$^+$: 569.3480; found: 569.3474.

IR ν_{max} (neat)/cm^{-1}: 3487, 2960, 1079, 841.

[α]$_D^{22}$= -4.7 (*c* 0.8, CHCl$_3$).

End game: deprotection, oxidation and dimerization

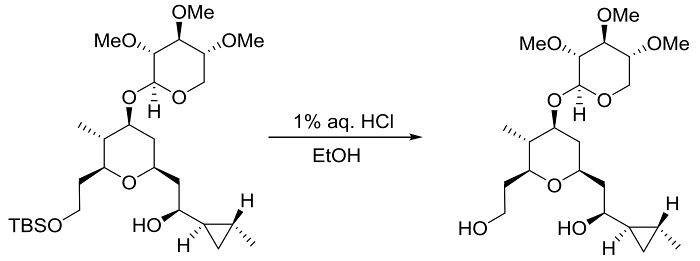

15a 23

To a solution of silyl ether **15a** (120 mg, 0.220 mmol, 1 equiv.) in EtOH (7.7 mL) was added 1% aq. HCl solution (2.3 mL). The reaction was stirred at room temperature for 20 min and quenched with sat. NaHCO$_3$ and extracted with EtOAc (x4). The combined organic layers were dried over MgSO$_4$, evaporated under reduced pressure and purified by flash chromatography (SiO$_2$, petroleum ether: EtOAc, 6:4 and then EtOAc) to give compound **23** as a colourless oil in 84% yield (80 mg, 0.18 mmol).

R_f= 0.26 (EtOAc).

^{1}H NMR (500 MHz, CDCl$_3$) δ (ppm) 4.24 (d, *J* = 7.1 Hz, 1H), 3.92 (dd, *J* = 11.4, 5.4 Hz, 1H), 3.77 – 3.66 (m, 2H), 3.59 (s, 3H), 3.56 (s, 3H), 3.53 – 3.50 (m, 1H), 3.44 (s, 3H), 3.27 – 3.14 (m, 3H), 3.12 – 2.97 (m, 3H), 2.99 – 2.88 (m, 1H), 2.78 (br. s, 1H), 2.04 (dd, *J* = 12.7, 4.7 Hz, 1H), 1.90 (d, *J* = 14.9 Hz, 1H), 1.85 – 1.71 (m, 1H), 1.69 – 1.56 (m, 2H), 1.43 (q, *J* = 10.0, 8.2 Hz, 2H), 1.01 (d, 3H),

0.97 (d, 3H), 0.71 (dt, J = 11.7, 5.5 Hz, 1H), 0.57 (tt, J = 8.6, 4.6 Hz, 1H), 0.29 (dt, J = 8.9, 4.7 Hz 1H), 0.20 (dt, J = 9.6, 4.8 Hz, 1H).

^{13}C NMR (125 MHz, CDCl$_3$) 105.6 (CH), 85.5 (CH), 83.8 (CH), 82.9 (CH), 80.7 (CH), 79.4 (CH), 75.8 (CH), 75.5 (CH), 63.2 (CH$_2$), 60.80 (CH$_3$), 60.76 (CH$_3$), 60.5 (CH$_2$), 58.8 (CH$_3$), 42.6 (CH$_2$), 42.2 (CH), 40.5 (CH$_2$), 34.9 (CH$_2$), 26.5 (CH), 18.7 (CH$_3$), 12.7 (CH$_3$), 11.1 (CH), 10.6 (CH$_2$).

HRMS (ESI) calc´d for $C_{22}H_{40}NaO_8$ [M+Na]$^+$: 455.2615; found: 455.2597.

IR ν_{max} (neat)/cm^{-1}: 3432, 2929, 1159, 1076, 985.

[α]$_D$^{22}= -18.0 (*c* 1, CHCl$_3$).

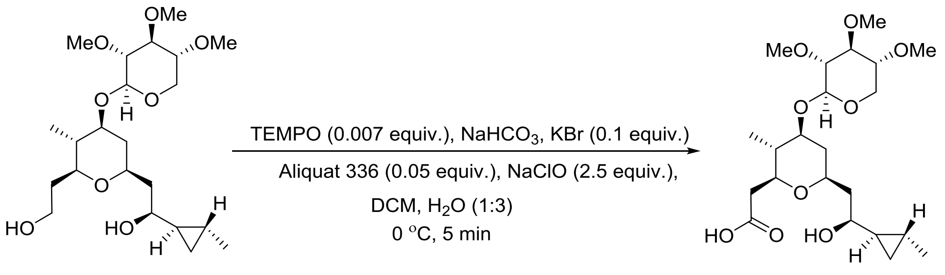

A flask at 0 °C was charged with diol **23** (60 mg, 0.14 mmol, 1 equiv.) in 0.17 mL of DCM, 87 μL of a 0.016M DCM solution of TEMPO, 0.087 μL of a 0.08M DCM solution of Aliquat 336 and 28 μL of a 0.5M aqueous solution of KBr. 0.99 mL of NaClO (0.35M) adjusted to pH 8.6 with $NaHCO_3$ was added and the mixture stirred at 1300 rpm for 5 min. The mixture was then diluted with H_2O and EtOAc, the phases were separated and the aqueous phase was acidified with 10% citric acid to pH 3-4. The aqueous layer was extracted with EtOAc (x4). The combined organic layers were dried over $MgSO_4$ and evaporated under reduced pressure. Compound **2** was obtained in 87% yield (54 mg, 0.12 mmol) as a colourless oil and was determined pure by ^{1}H and ^{13}C NMR and HRMS and was used without further purification before the next step.

^{1}H NMR (500 MHz, CDCl$_3$) δ (ppm) 4.29 (d, J = 7.6 Hz, 1H), 3.96 (dd, J = 11.5, 5.2 Hz, 1H), 3.63 (s, 3H), 3.60 (s, 3H), 3.56 – 3.50 (m, 1H), 3.49 (s, 3H), 3.32 – 3.23 (m, 2H), 3.20 – 3.14 (m, 1H), 3.14 – 3.07 (m, 3H), 2.98 (dd, J = 9.1, 7.6 Hz, 1H), 2.69 (dd, J = 15.0, 2.3 Hz, 1H), 2.46 –2.38 (m, 1H), 2.10 (dd, J = 12.7, 4.7 Hz, 1H), 1.85 – 1.76 (m, 1H), 1.74 – 1.66 (m, 1H), 1.58 – 1.41 (m, 2H), 1.03 (app t, J = 6.4 Hz, 6H), 0.80 – 0.72 (m, 1H), 0.60 (tt, J = 8.5, 4.3 Hz, 1H), 0.30 – 0.21 (dt, J = 8.5, 4.6 Hz, 1H), 0.19 (dt, J = 9.3, 4.8 Hz, 1H).

^{13}C NMR (126 MHz, CDCl$_3$) δ (ppm) 173.3 (C), 105.6 (CH), 85.5 (CH), 83.8 (CH), 82.4 (CH), 79.3 (CH), 78.3 (CH), 77.4 (CH), 76.7 (CH), 63.2 (CH$_2$), 60.84 (CH$_3$), 60.80 (CH$_3$), 58.8 (CH$_3$), 42.0 (CH), 41.6 (CH$_2$), 40.5 (CH$_2$), 38.5 (CH$_2$), 25.7 (CH), 18.5 (CH$_3$), 12.7 (CH$_3$), 11.8 (CH), 10.2 (CH$_2$).

HRMS (ESI) calc´d for $C_{22}H_{38}NaO_9$ [M+Na]$^+$: 469.2408; found: 469.2393.

IR ν_{max} (neat)/cm^{-1}: 3450, 1730, 1460, 1375, 1325, 1230, 1170, 1081.

[α]$_D$^{22}= -18.0 (*c* 1, CHCl$_3$).

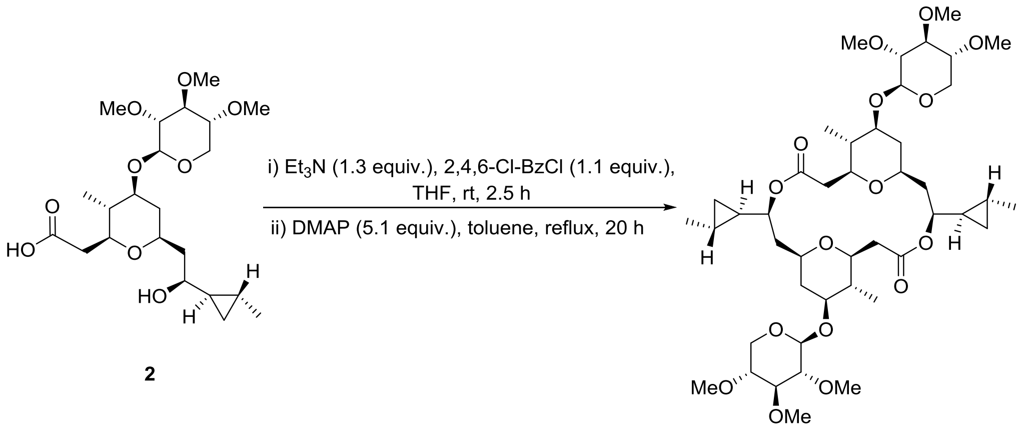

2

clavosolide A

2,4,6-Trichlorobenzoyl chloride (8 μL, 0.05 mmol, 1.1 equiv.) was added to a stirring solution of seco-acid **2** (20 mg, 0.045 mmol, 1 equiv.) and Et_3N (8 μL, 0.06 mmol, 1.3 equiv.) in dry THF (0.16 mL) at rt under N_2. The mixture was stirred for 2.5 h. The $Et_3N{\cdot}HCl$ salt was filtered off and the filtrate was diluted with dry toluene (1.83 mL). This solution was then added dropwise to a solution of DMAP (30 mg, 0.25 mmol, 5.1 equiv.) in dry toluene (8.5 mL) under reflux over 4 h. The mixture was stirred for a further 16 h at reflux and diluted with water. The layers were separated and the aqueous layer was extracted with Et_2O (x3). The combined organic layers were washed with brine (x1), dried over $MgSO_4$ evaporated under reduced pressure and purified by flash chromatography (SiO_2, petroleum ether: EtOAc, 1:1) to give the natural product as a white solid in 68% yield (13 mg, 0.015 mmol).

R_f= 0.55 (petroleum: EtOAc, 3:7).

^{1}H NMR (500 MHz, $CDCl_3$) δ (ppm) 4.41 (td, *J* = 8.9, 1.9 Hz, 2H), 4.26 (d, *J* = 7.6 Hz, 2H), 3.95 (dd, *J* = 11.6, 5.2 Hz, 2H), 3.61 (s, 6H), 3.58 (s, 6H), 3.46 (s, 6H), 3.46 – 3.41 (m, 4H), 3.30 – 3.20 (m, 4H), 3.11 (td, *J* = 10.1, 9.5, 4.5 Hz, 4H), 2.96 (dd, *J* = 9.1, 7.6 Hz, 2H), 2.54 (dd, *J* = 17.4, 3.6 Hz, 2H), 2.41 (dd, *J* = 17.3, 6.6 Hz, 2H), 2.04 (ddd, *J* = 12.7, 5.0, 1.8 Hz, 2H), 1.88 (dt, *J* = 15.1, 8.9 Hz, 2H), 1.68 (dd, *J* = 15.1, 8.9 Hz, 2H), 1.42 – 1.33 (m, 4H), 0.96 (d, *J* = 6.2 Hz, 12H), 0.90 – 0.77 (m, 2H), 0.71 (tt, *J* = 8.8, 4.6 Hz, 2H), 0.34 (dt, *J* = 8.5, 4.9 Hz, 2H), 0.22 (dt, *J* = 8.5, 4.9 Hz, 2H).

^{13}C NMR (125 MHz, $CDCl_3$) δ (ppm) 171.0 (2xC), 105.5 (2xCH), 85.6 (2xCH), 83.8 (2xCH), 83.2 (2xCH), 79.4 (2xCH), 77.1 (2xCH), 77.0 (2xCH), 74.8 (2xCH), 63.2 (2xCH_2), 60.8 (4xCH_3), 58.8 (2xCH_3) , 42.5 (2xCH), 41.3 (2xCH_2), 40.7 (2xCH_2), 39.2 (2xCH_2), 24.7 (2xCH), 18.5 (2xCH_3), 12.6 (2xCH_3), 11.9 (2xCH), 10.9 (2xCH_2).

HRMS (ESI) calc´d for $C_{44}H_{72}NaO_{16}$ $[M+Na]^+$: 879.4713; found: 879.4716.

IR ν_{max} (neat)/cm^{-1}: 2933, 1720, 1207, 1080.

m.p.= 239-242°C.

$[\alpha]_D^{22}$= -43.7 (*c* 0.016, $CHCl_3$).

5. ^{13}C and ^{1}H NMR data of natural and synthetic (–)-clavosolide A

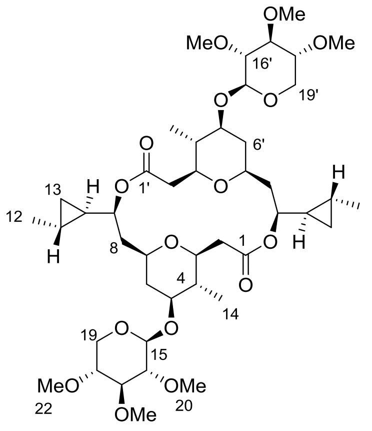

clavosolide A

C#	δC natural	δC synthetic	ΔδC	δH natural	δH synthetic	ΔδH
1, 1′	170.7	171.01	+0.69			
2, 2′	39.3	39.22	-0.08	2.55<br>2.41	2.54<br>2.41	-0.01<br>0.00
3, 3′	77.0	77.00	0.00	3.42	3.43	+0.01
4, 4′	42.6	42.52	-0.08	1.38	1.38	0.00
5, 5′	83.1	83.18	+0.08	3.25	3.25	0.00
6, 6′	40.8	40.69	-0.11	2.05<br>1.37	2.04<br>1.38	-0.01
7, 7′	74.8	74.8	0.00	3.42	3.43	+0.01
8, 8′	41.1	41.27	+0.17	1.87<br>1.66	1.88<br>1.68	+0.01<br>+0.02
9, 9′	77.1	77.10	0.00	4.41	4.41	0.00
10, 10′	24.8	24.73	-0.07	0.72	0.71	-0.01
11, 11′	12.0	11.93	-0.07	0.83	0.83	0.00
12, 12′	18.6	18.50	-0.10	0.96	0.96	0.00
13, 13′	11.0	10.91	-0.09	0.22<br>0.33	0.22<br>0.34	0.00<br>+0.01
14, 14′	12.7	12.61	-0.09	0.96	0.96	0.00
15, 15′	105.4	105.50	+0.10	4.27	4.26	-0.01
16, 16′	83.8	83.83	+0.03	2.96	2.96	0.00
17, 17′	85.6	85.56	-0.04	3.12	3.11	-0.03
18, 18′	79.4	79.38	-0.02	3.25	3.25	0.00
19, 19′	63.2	63.23	+0.03	3.96<br>3.10	3.95<br>3.11	-0.01<br>+0.01
20, 20′	60.7	60.80	+0.10	3.57	3.58	+0.01
21, 21′	60.8	60.80	0.00	3.62	3.61	-0.01
22, 22′	58.5	58.80	+0.30	3.47	3.46	-0.01

Synthetic compound: **$[\alpha]_D^{22}$**= -43.7 (*c* 1, $CHCl_3$)

Natural compound: **$[\alpha]_D^{22}$**= -48.5 (*c* 0.02, $CHCl_3$)

6. ^{13}C and ^{1}H NMR spectra

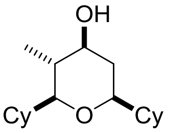

8a

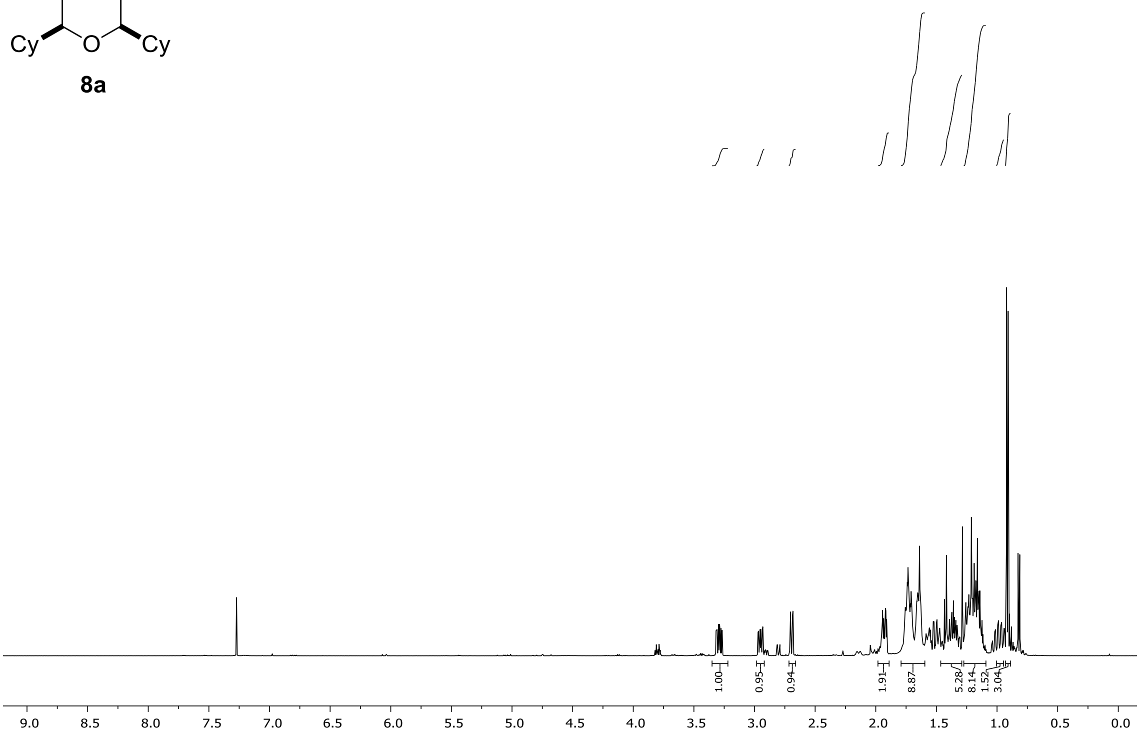

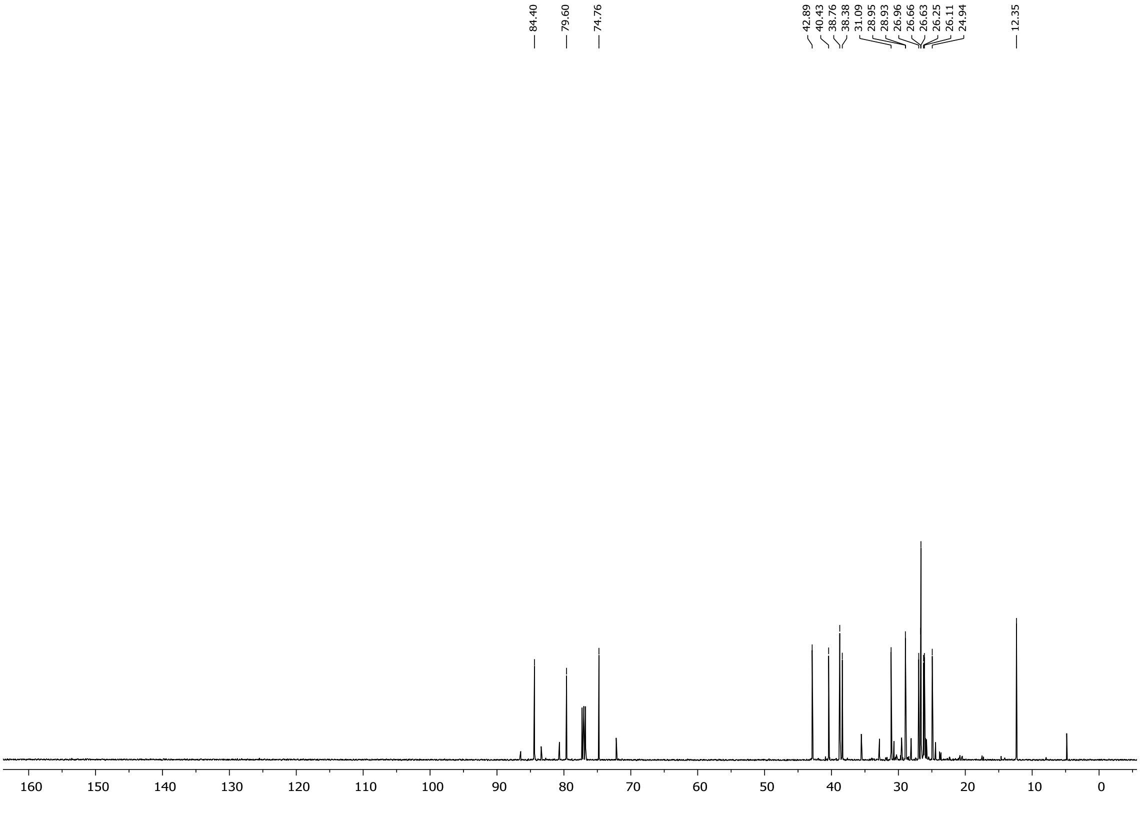

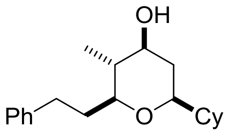
OH
Ph
O
Cy

8b

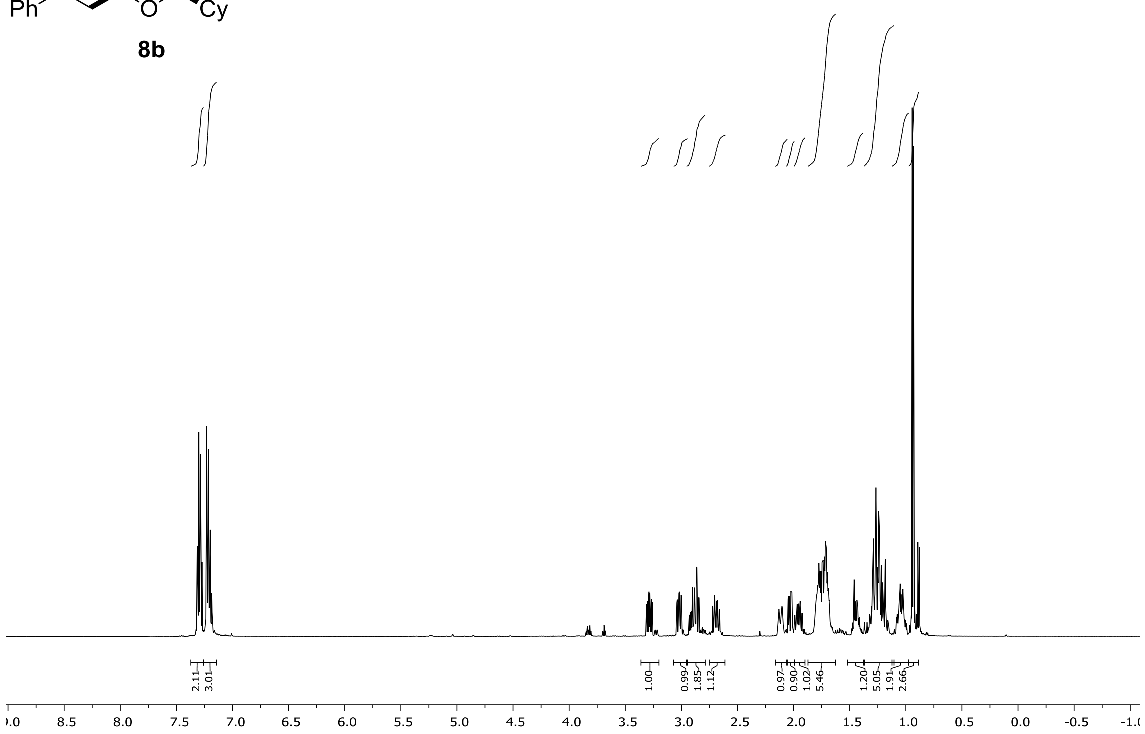
2.11
3.01
1.00
0.99
1.85
1.12
0.97
0.90
1.02
5.46
1.20
5.05
1.91
2.66

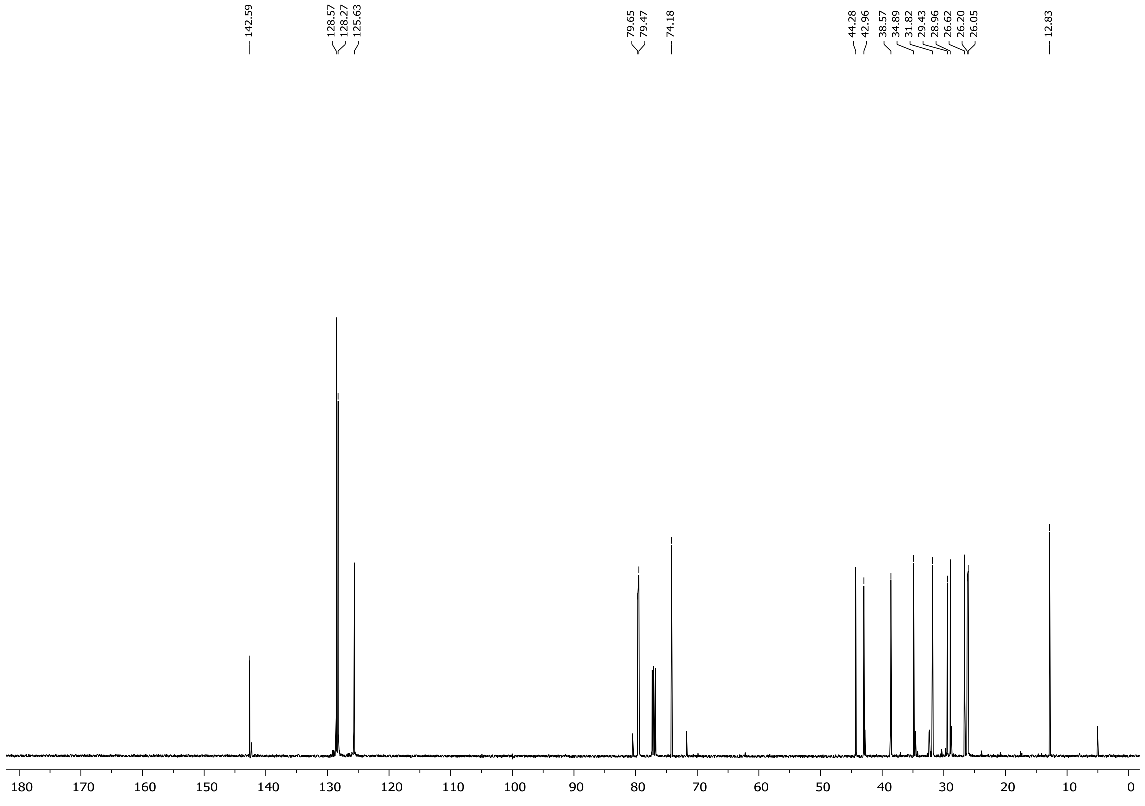
142.59
128.57
128.27
125.63
79.65
79.47
74.18
44.28
42.96
38.57
34.89
31.82
29.43
28.96
26.62
26.20
26.05
12.83

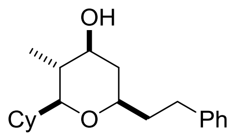
OH
Cy
O
Ph

8c

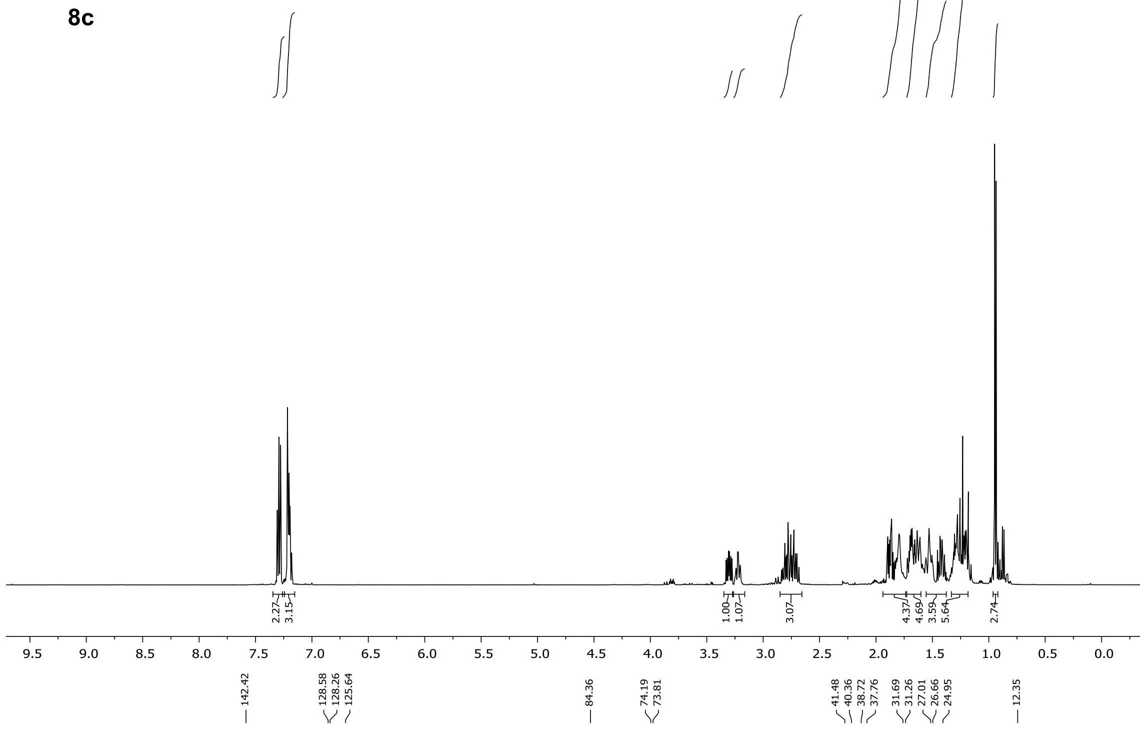
2.27
3.15
1.00
1.07
3.07
4.37
4.69
3.59
5.64
2.74
9.5 9.0 8.5 8.0 7.5 7.0 6.5 6.0 5.5 5.0 4.5 4.0 3.5 3.0 2.5 2.0 1.5 1.0 0.5 0.0
142.42
128.58
128.26
125.64
84.36
74.19
73.81
41.48
40.36
38.72
37.76
31.69
31.26
27.01
26.66
24.95
12.35

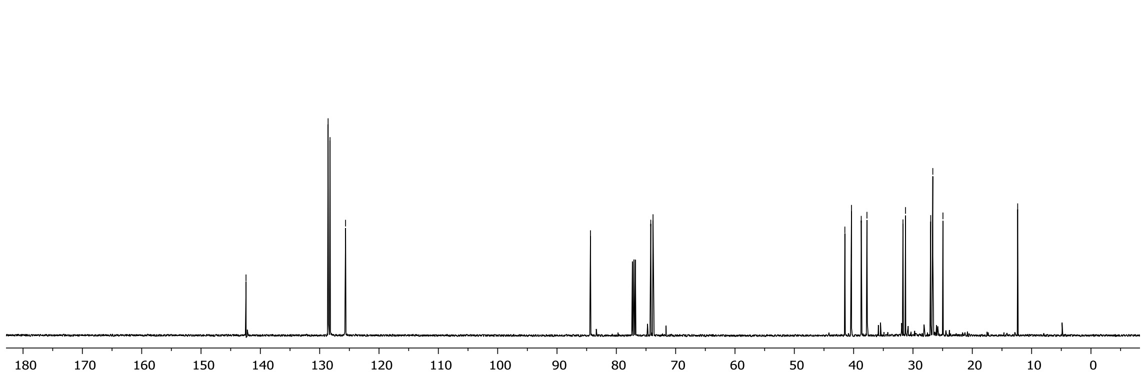
180 170 160 150 140 130 120 110 100 90 80 70 60 50 40 30 20 10 0

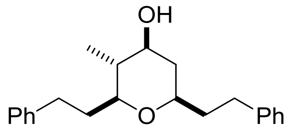
OH
Ph
O
Ph

8d

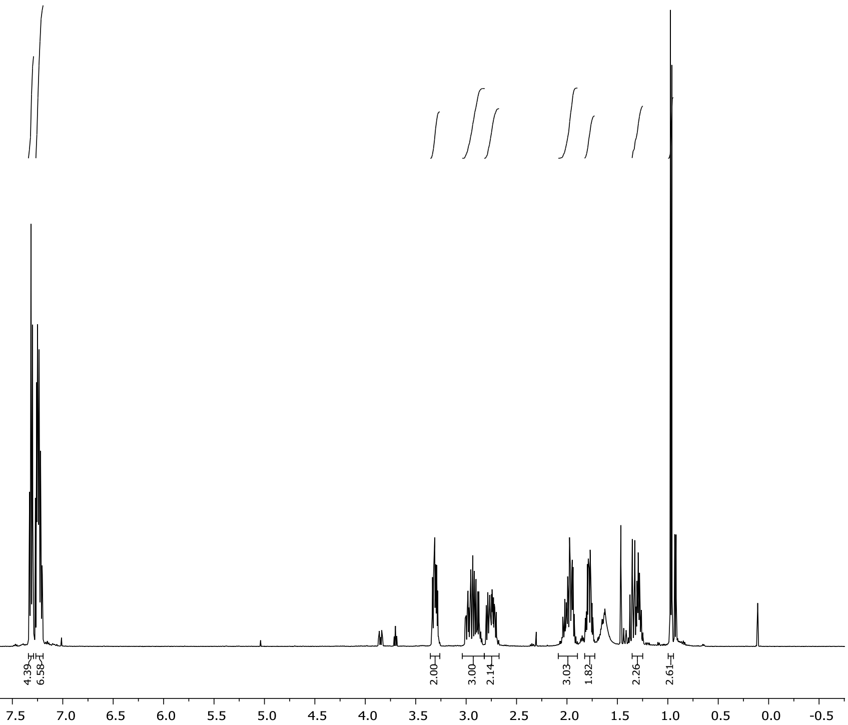
4.39
6.58
2.00
3.00
2.14
3.03
1.82
2.26
2.61
10.0 9.5 9.0 8.5 8.0 7.5 7.0 6.5 6.0 5.5 5.0 4.5 4.0 3.5 3.0 2.5 2.0 1.5 1.0 0.5 0.0 -0.5

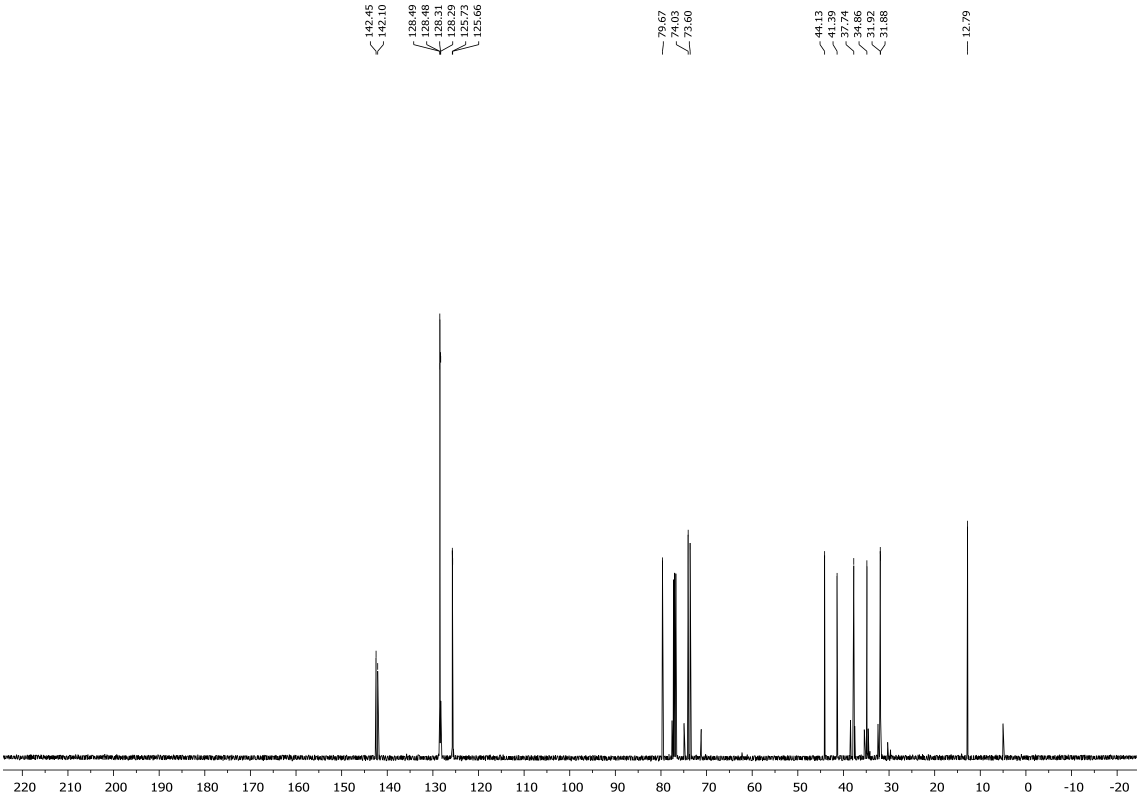
142.45
142.10
128.49
128.48
128.31
128.29
125.73
125.66
79.67
74.03
73.60
44.13
41.39
37.74
34.86
31.92
31.88
12.79
220 210 200 190 180 170 160 150 140 130 120 110 100 90 80 70 60 50 40 30 20 10 0 -10 -20

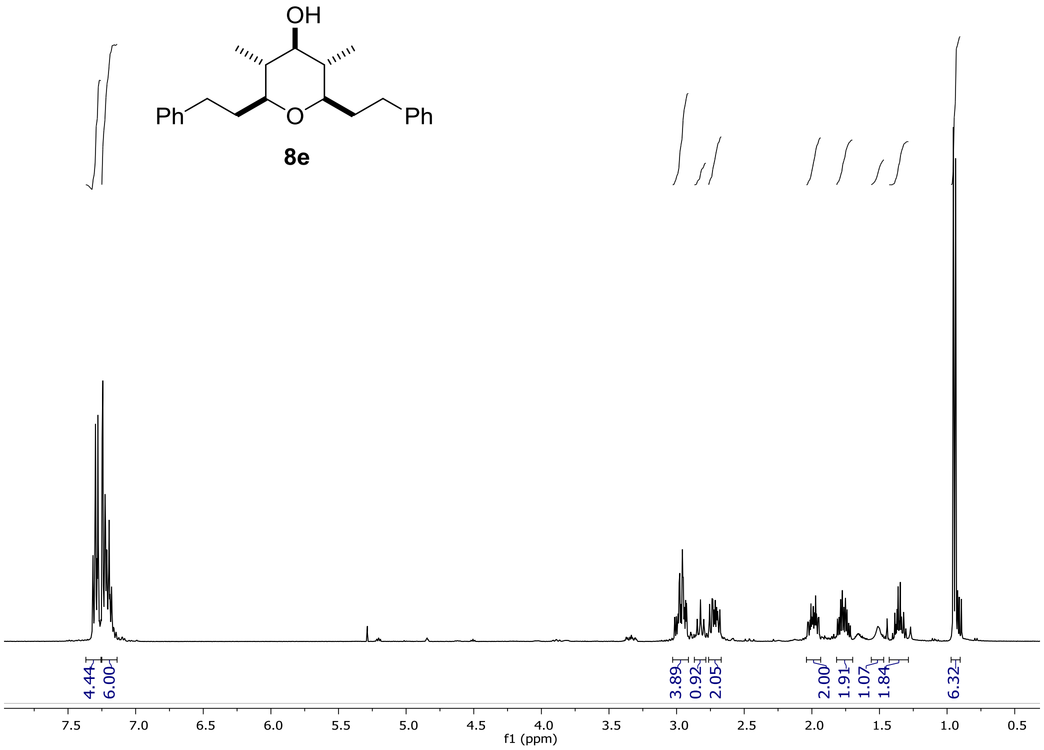
OH
Ph
O
Ph
8e
4.44
6.00
3.89
0.92
2.05
2.00
1.91
1.07
1.84
6.32
7.5 7.0 6.5 6.0 5.5 5.0 4.5 4.0 3.5 3.0 2.5 2.0 1.5 1.0 0.5
f1 (ppm)

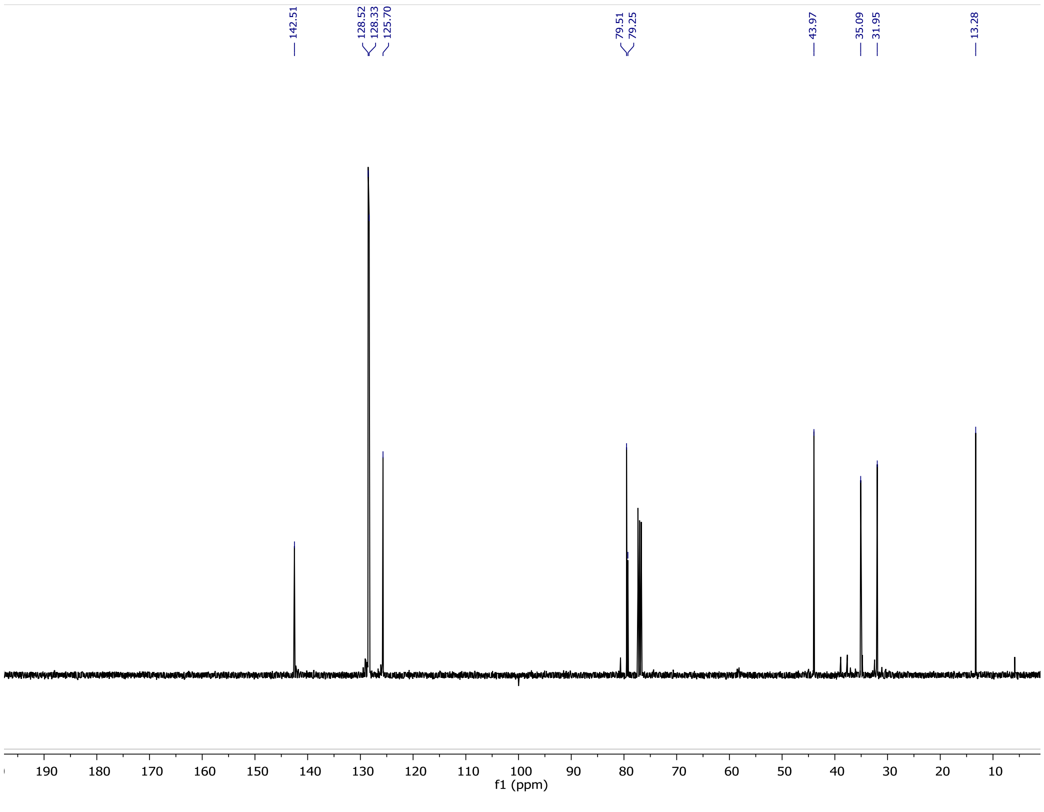
142.51
128.52
128.33
125.70
79.51
79.25
43.97
35.09
31.95
13.28
190 180 170 160 150 140 130 120 110 100 90 80 70 60 50 40 30 20 10
f1 (ppm)

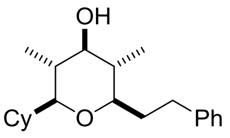

8f

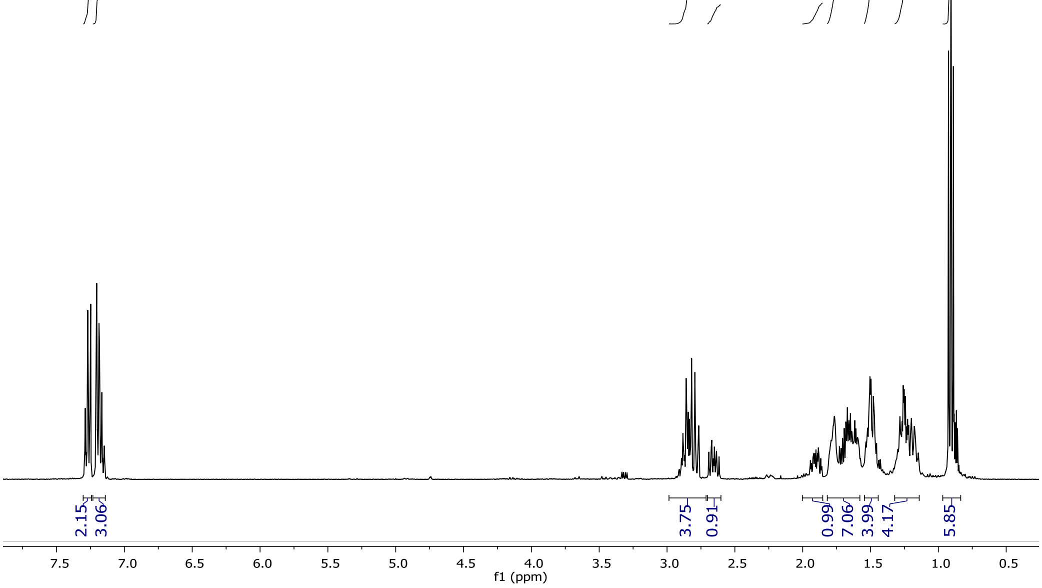
2.15
3.06
3.75
0.91
0.99
7.06
3.99
4.17
5.85
f1 (ppm)

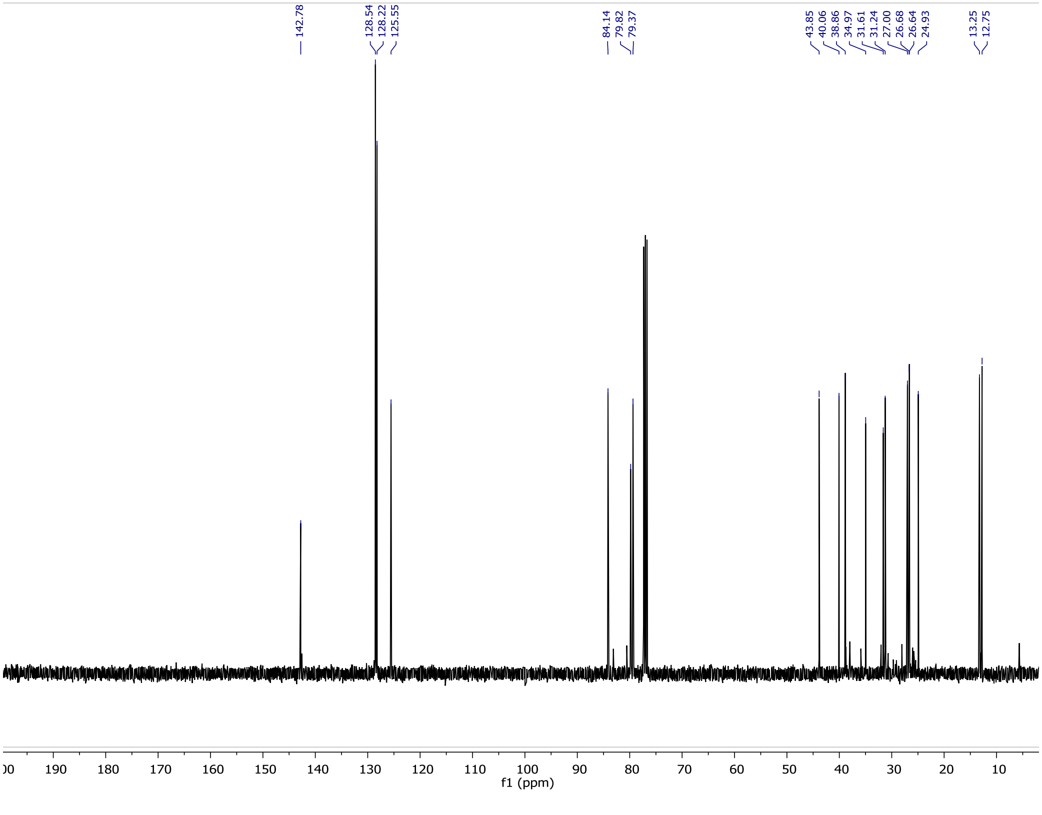
142.78
128.54
128.22
125.55
84.14
79.82
79.37
43.85
40.06
38.86
34.97
31.61
31.24
27.00
26.68
26.64
24.93
13.25
12.75
f1 (ppm)

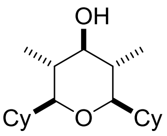
OH
Cy
O
Cy

8g

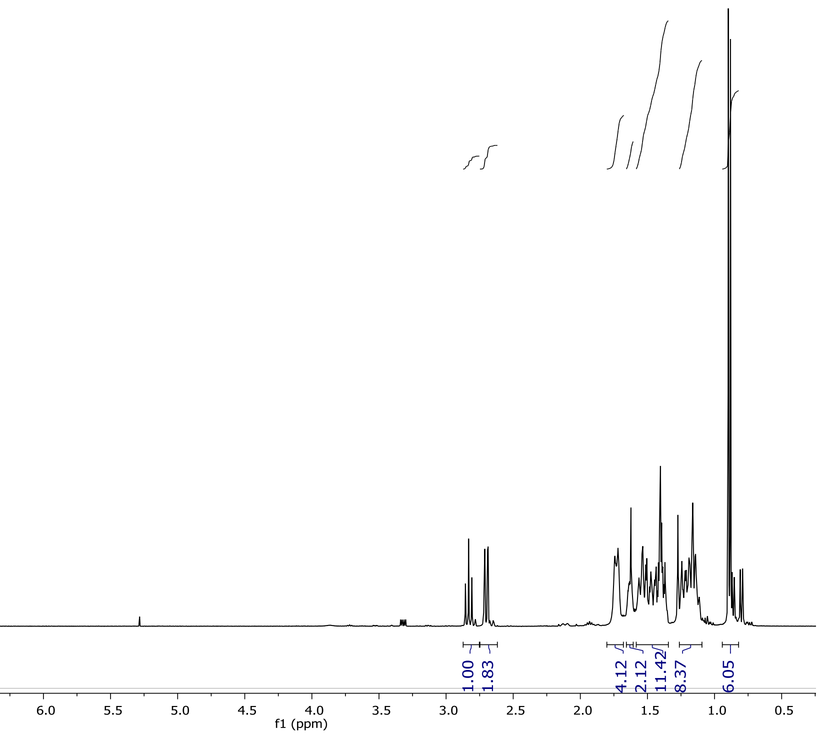
1.00
1.83
4.12
2.12
11.42
8.37
6.05
7.5
7.0
6.5
6.0
5.5
5.0
4.5
4.0
3.5
3.0
2.5
2.0
1.5
1.0
0.5
f1 (ppm)

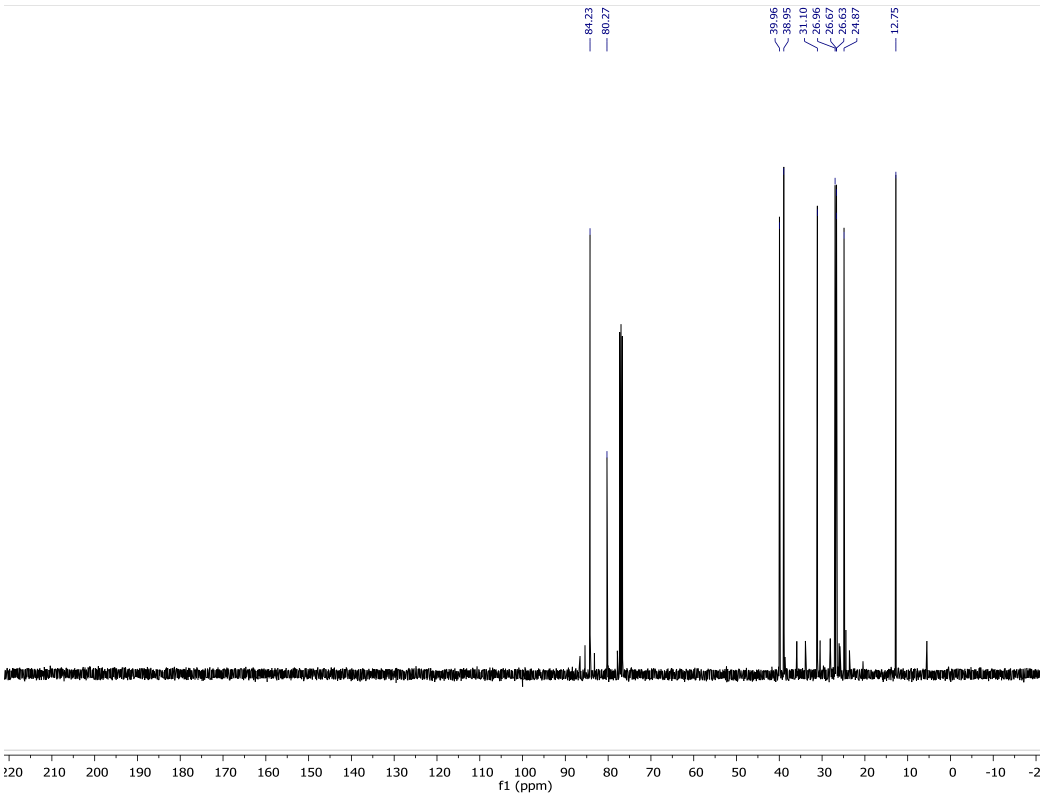
84.23
80.27
39.96
38.95
31.10
26.96
26.67
26.63
24.87
12.75
210
200
190
180
170
160
150
140
130
120
110
100
90
80
70
60
50
40
30
20
10
0
-10
f1 (ppm)

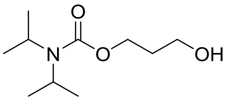

O
N
O
OH

19a

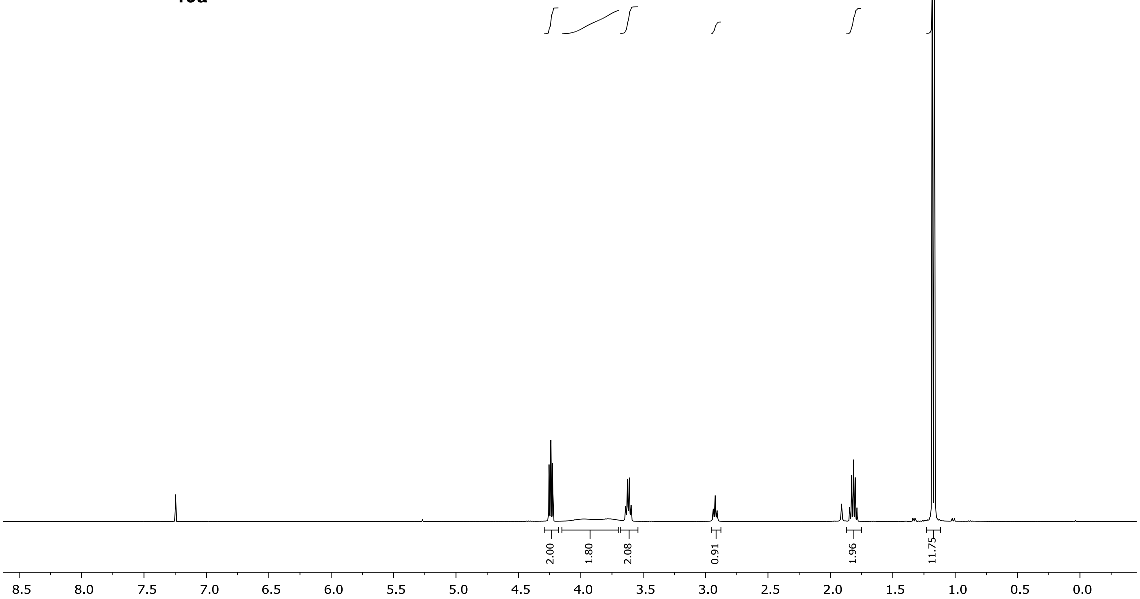

2.00
1.80
2.08
0.91
1.96
11.75
8.5 8.0 7.5 7.0 6.5 6.0 5.5 5.0 4.5 4.0 3.5 3.0 2.5 2.0 1.5 1.0 0.5 0.0

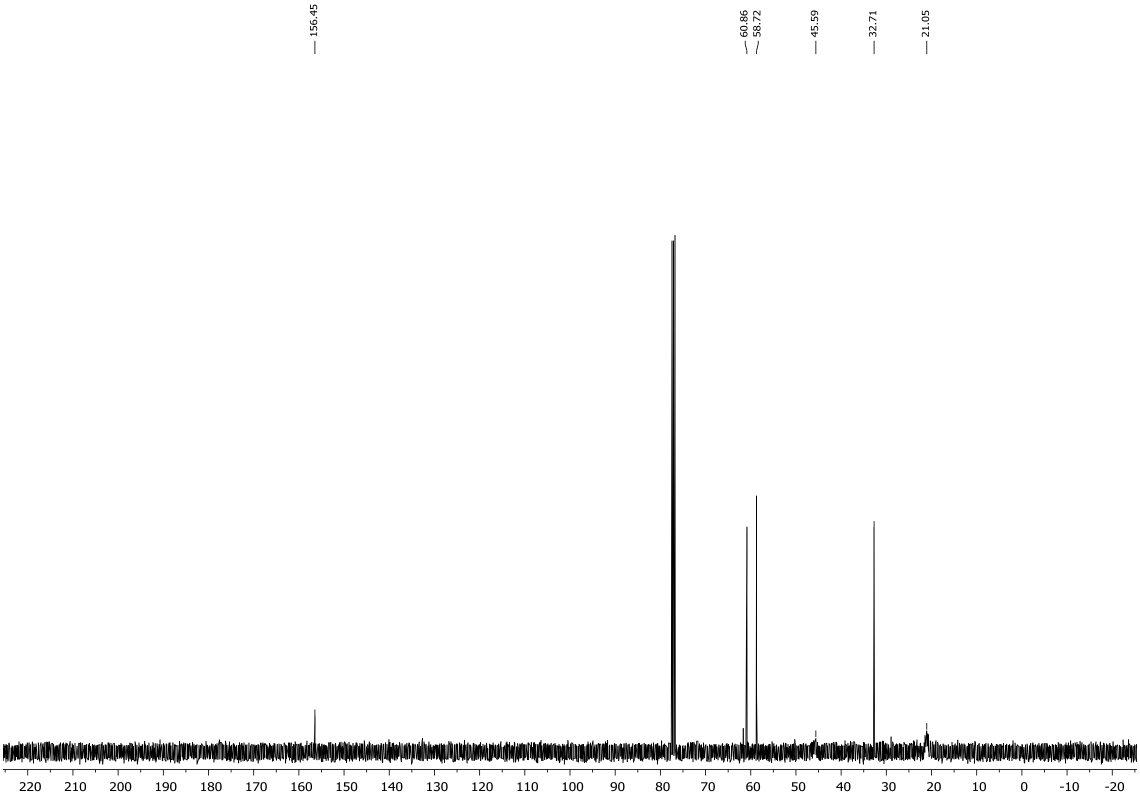

156.45
60.86
58.72
45.59
32.71
21.05
220 210 200 190 180 170 160 150 140 130 120 110 100 90 80 70 60 50 40 30 20 10 0 -10 -20

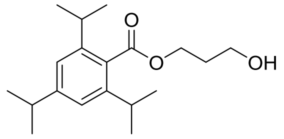
O
O
OH

19b

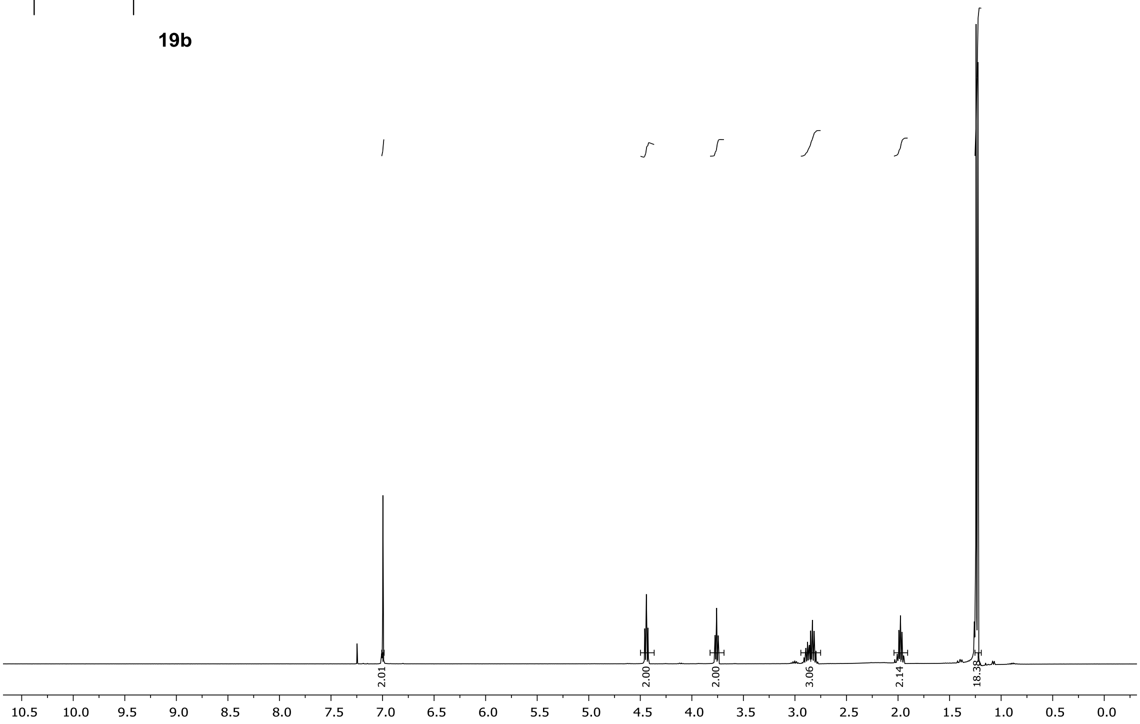
2.01
2.00
2.00
3.06
2.14
18.38
10.5 10.0 9.5 9.0 8.5 8.0 7.5 7.0 6.5 6.0 5.5 5.0 4.5 4.0 3.5 3.0 2.5 2.0 1.5 1.0 0.5 0.0

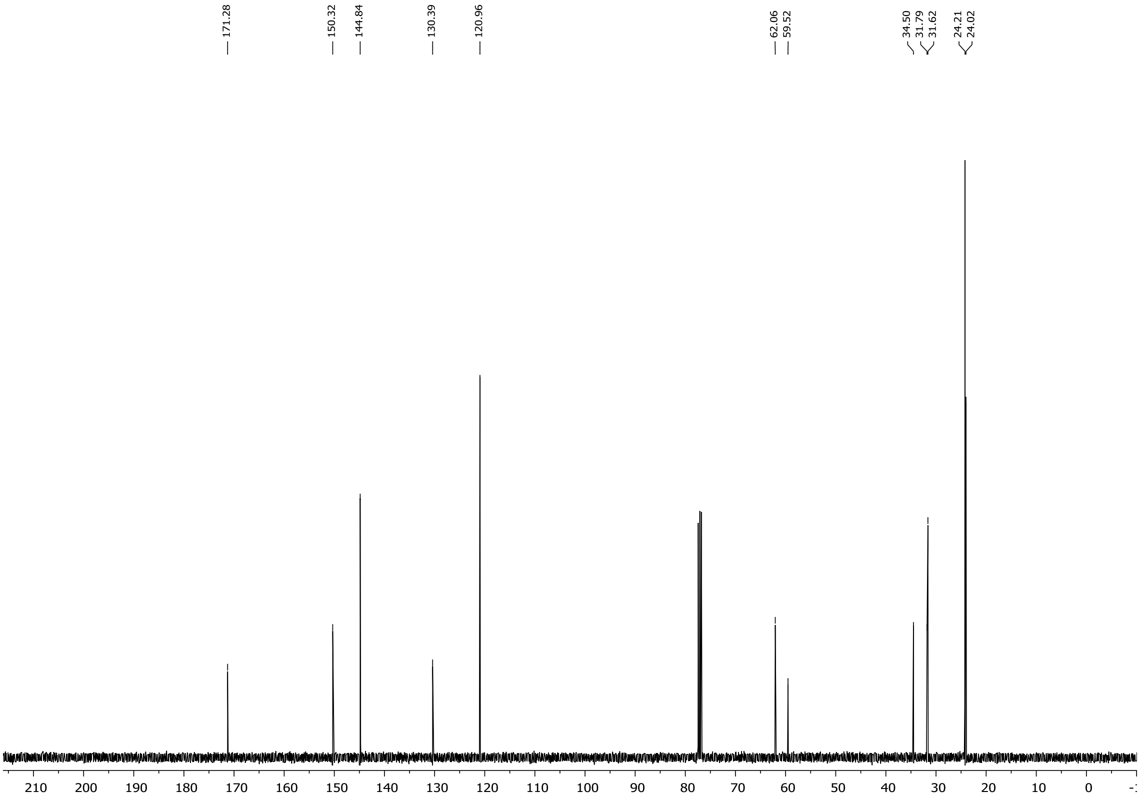
171.28
150.32
144.84
130.39
120.96
62.06
59.52
34.50
31.79
31.62
24.21
24.02
210 200 190 180 170 160 150 140 130 120 110 100 90 80 70 60 50 40 30 20 10 0

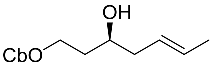
OH
CbO

5a

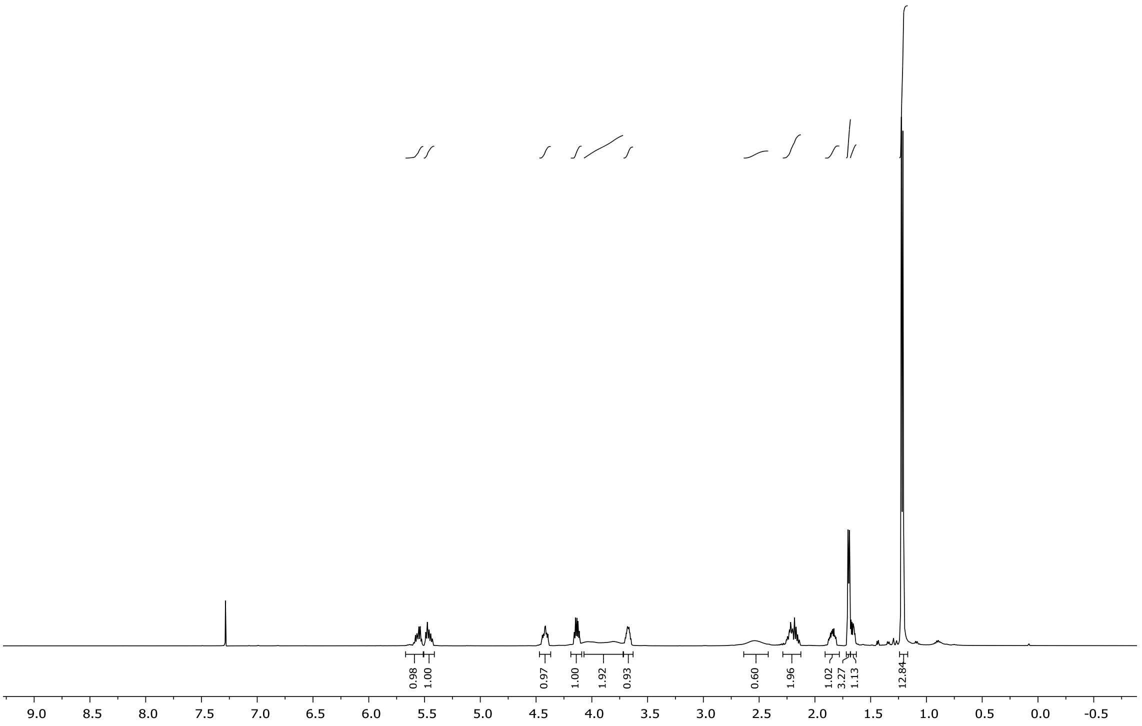
0.98
1.00
0.97
1.00
1.92
0.93
0.60
1.96
1.02
3.27
1.13
12.84
9.0 8.5 8.0 7.5 7.0 6.5 6.0 5.5 5.0 4.5 4.0 3.5 3.0 2.5 2.0 1.5 1.0 0.5 0.0 -0.5

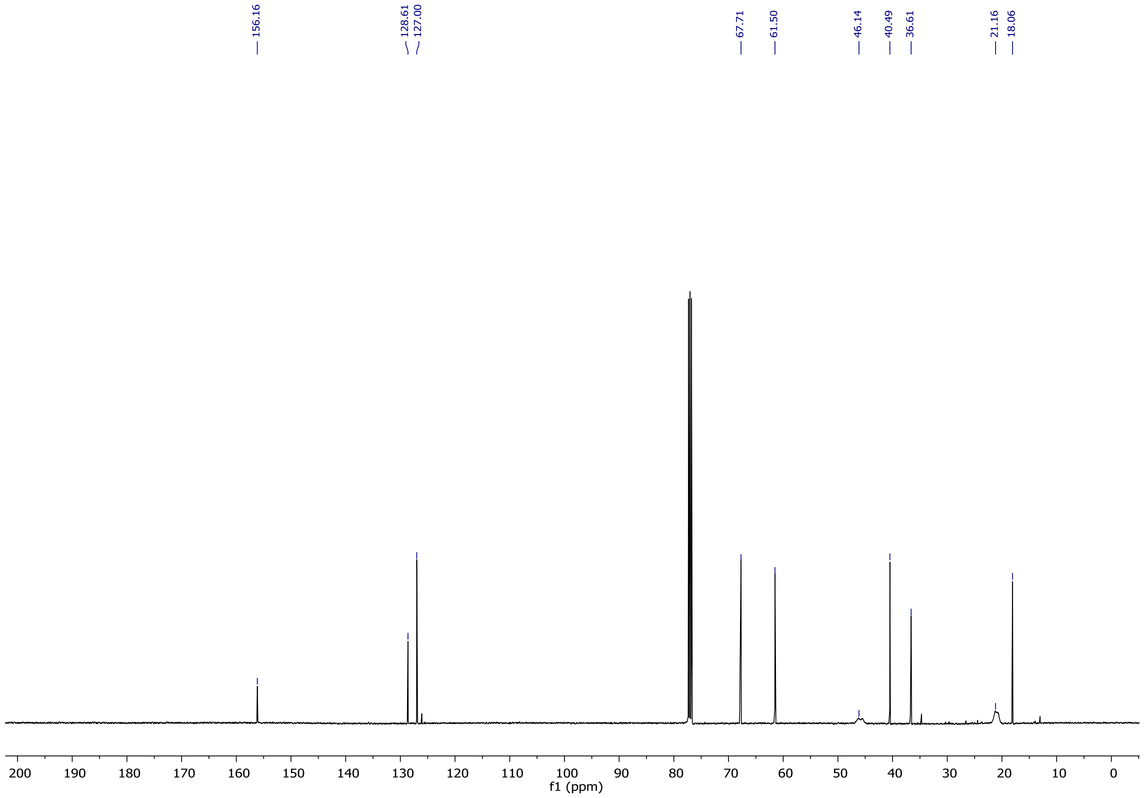
156.16
128.61
127.00
67.71
61.50
46.14
40.49
36.61
21.16
18.06
200 190 180 170 160 150 140 130 120 110 100 90 80 70 60 50 40 30 20 10 0
f1 (ppm)

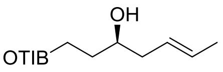
OH
OTIB

5b

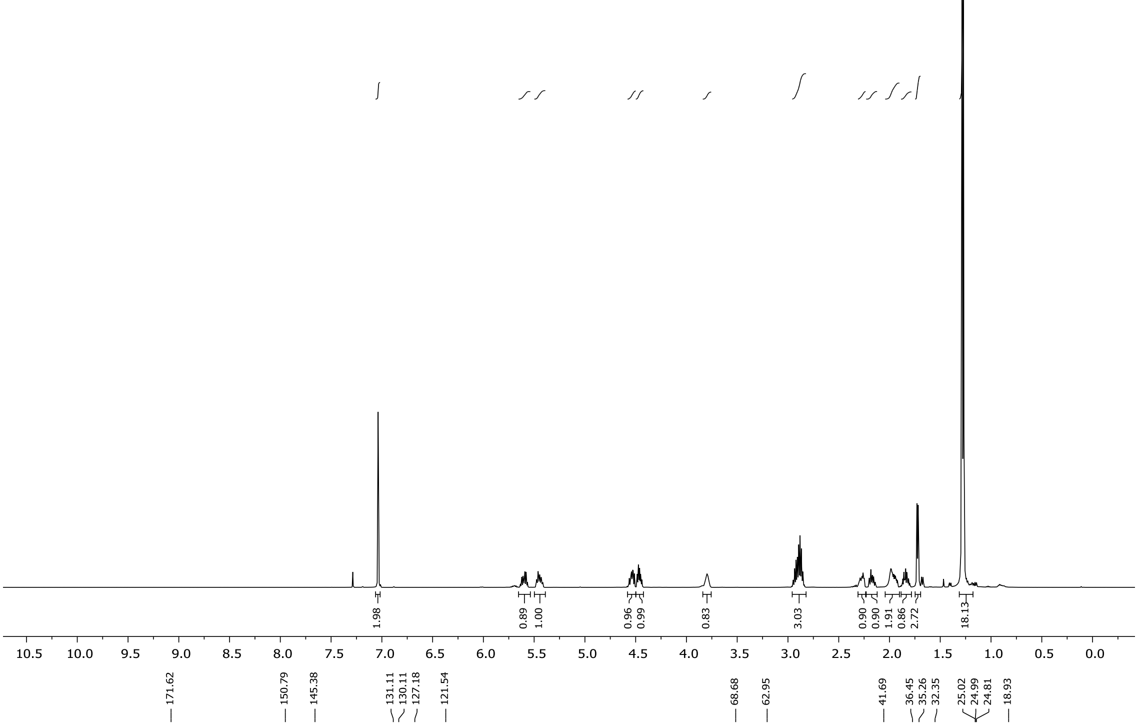
1.98
0.89
1.00
0.96
0.99
0.83
3.03
0.90
0.90
1.91
0.86
2.72
18.13
10.5 10.0 9.5 9.0 8.5 8.0 7.5 7.0 6.5 6.0 5.5 5.0 4.5 4.0 3.5 3.0 2.5 2.0 1.5 1.0 0.5 0.0

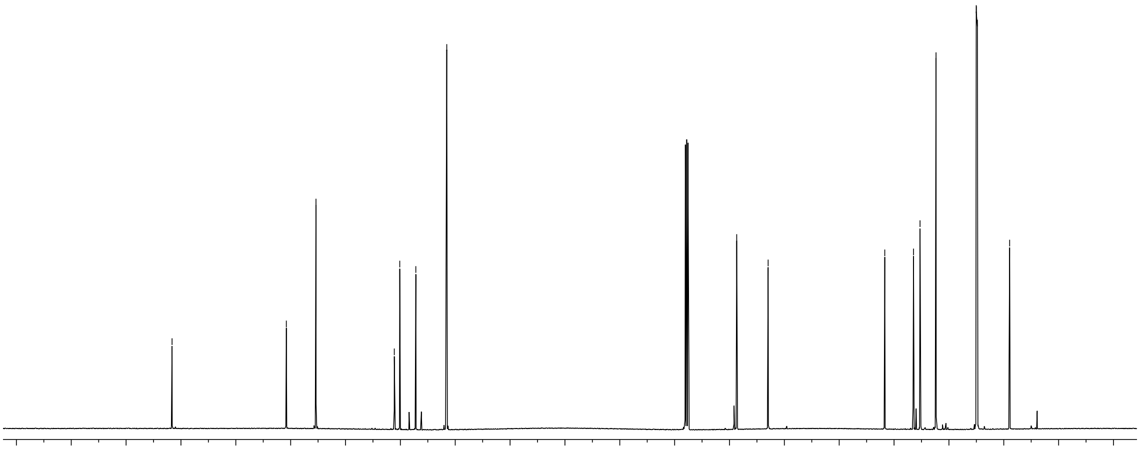
171.62
150.79
145.38
131.11
130.11
127.18
121.54
68.68
62.95
41.69
36.45
35.26
32.35
25.02
24.99
24.81
18.93
200 190 180 170 160 150 140 130 120 110 100 90 80 70 60 50 40 30 20 10 0

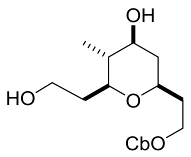
OH
HO
O
CbO

11

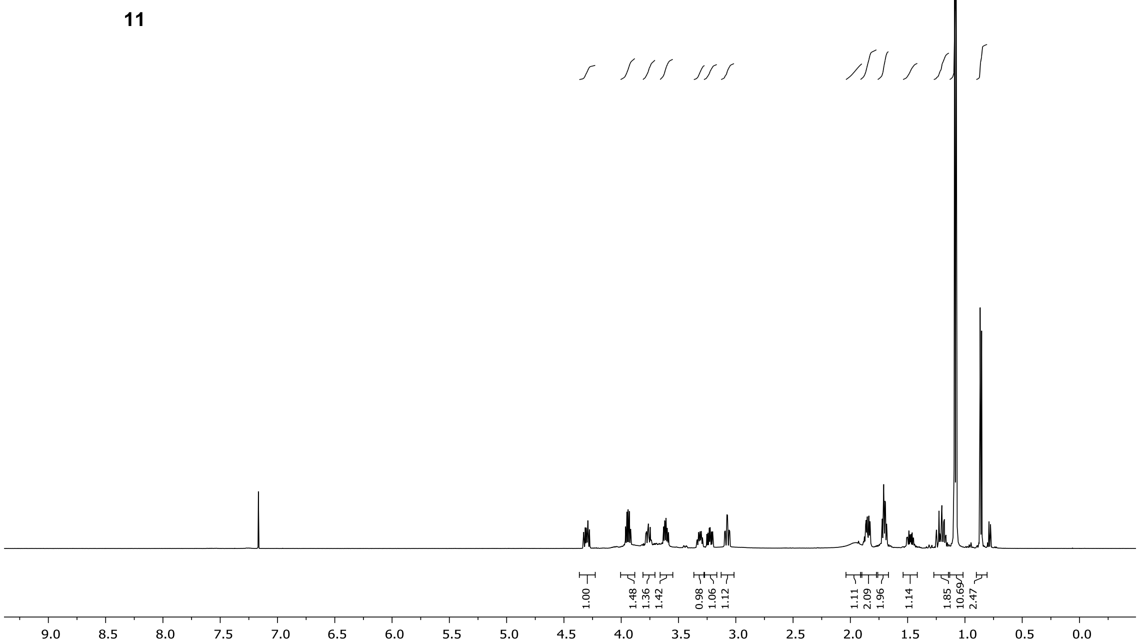
1.00
1.48
1.36
1.42
0.98
1.06
1.12
1.11
2.09
1.96
1.14
1.85
10.69
2.47
9.0 8.5 8.0 7.5 7.0 6.5 6.0 5.5 5.0 4.5 4.0 3.5 3.0 2.5 2.0 1.5 1.0 0.5 0.0

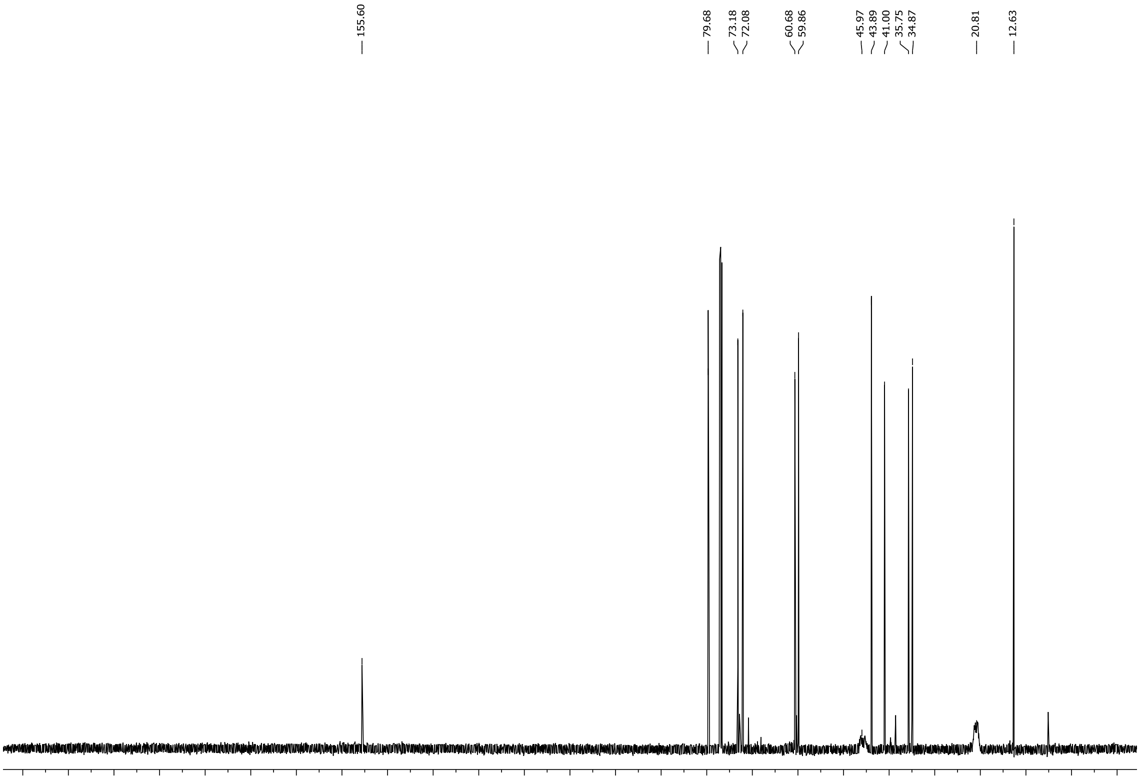
155.60
79.68
73.18
72.08
60.68
59.86
45.97
43.89
41.00
35.75
34.87
20.81
12.63
230 220 210 200 190 180 170 160 150 140 130 120 110 100 90 80 70 60 50 40 30 20 10 0 -10

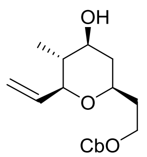
OH
O
CbO

12a

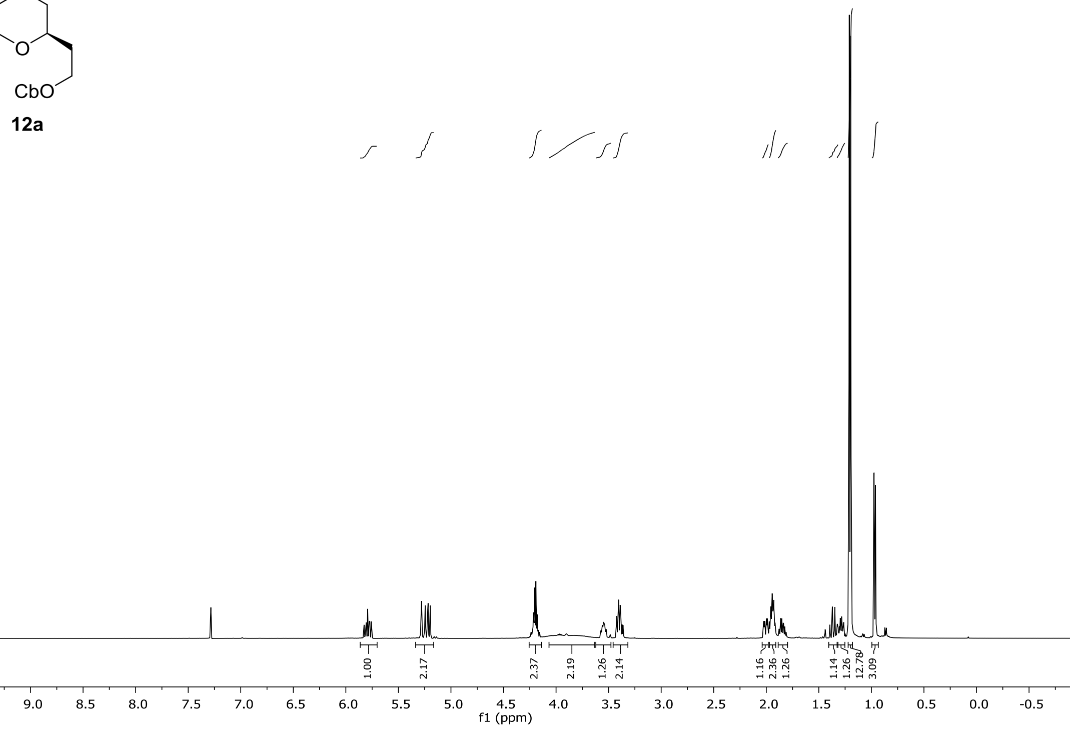
1.00
2.17
2.37
2.19
1.26
2.14
1.16
2.36
1.26
1.14
1.26
12.78
3.09
9.5 9.0 8.5 8.0 7.5 7.0 6.5 6.0 5.5 5.0 4.5 4.0 3.5 3.0 2.5 2.0 1.5 1.0 0.5 0.0 -0.5
f1 (ppm)

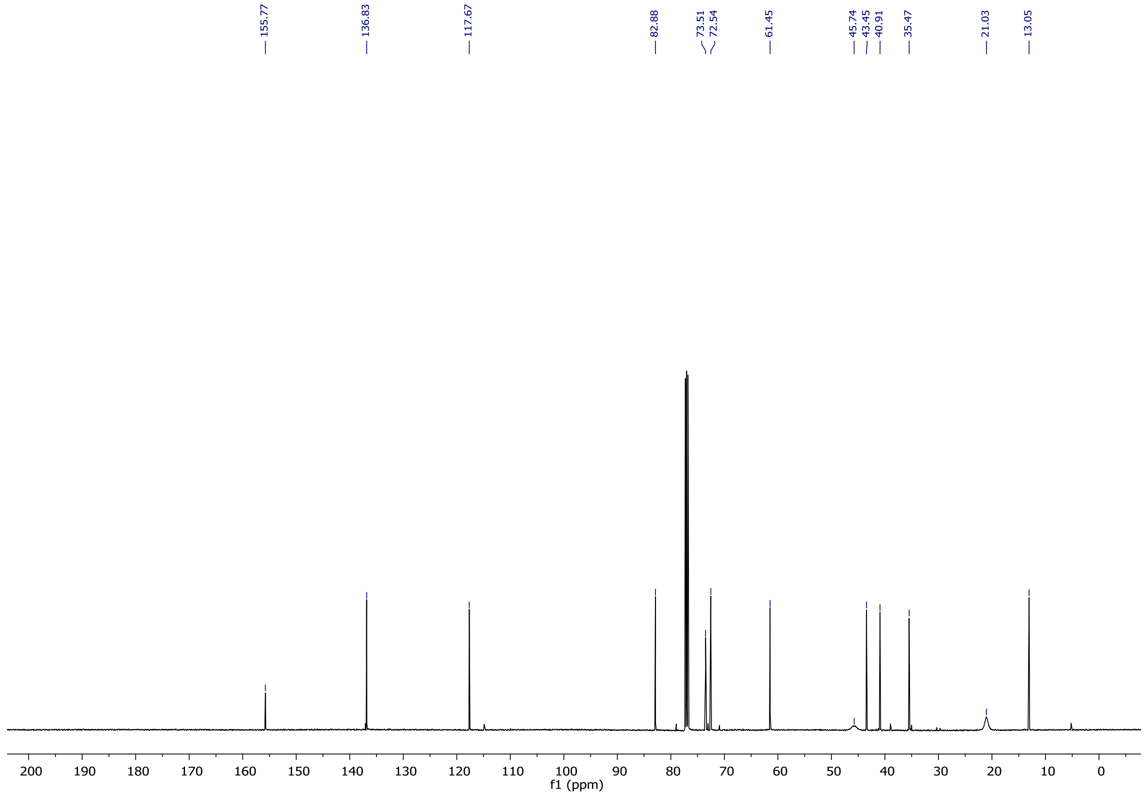
155.77
136.83
117.67
82.88
73.51
72.54
61.45
45.74
43.45
40.91
35.47
21.03
13.05
200 190 180 170 160 150 140 130 120 110 100 90 80 70 60 50 40 30 20 10 0
f1 (ppm)

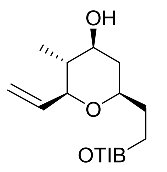
OH
O
OTIB

12b

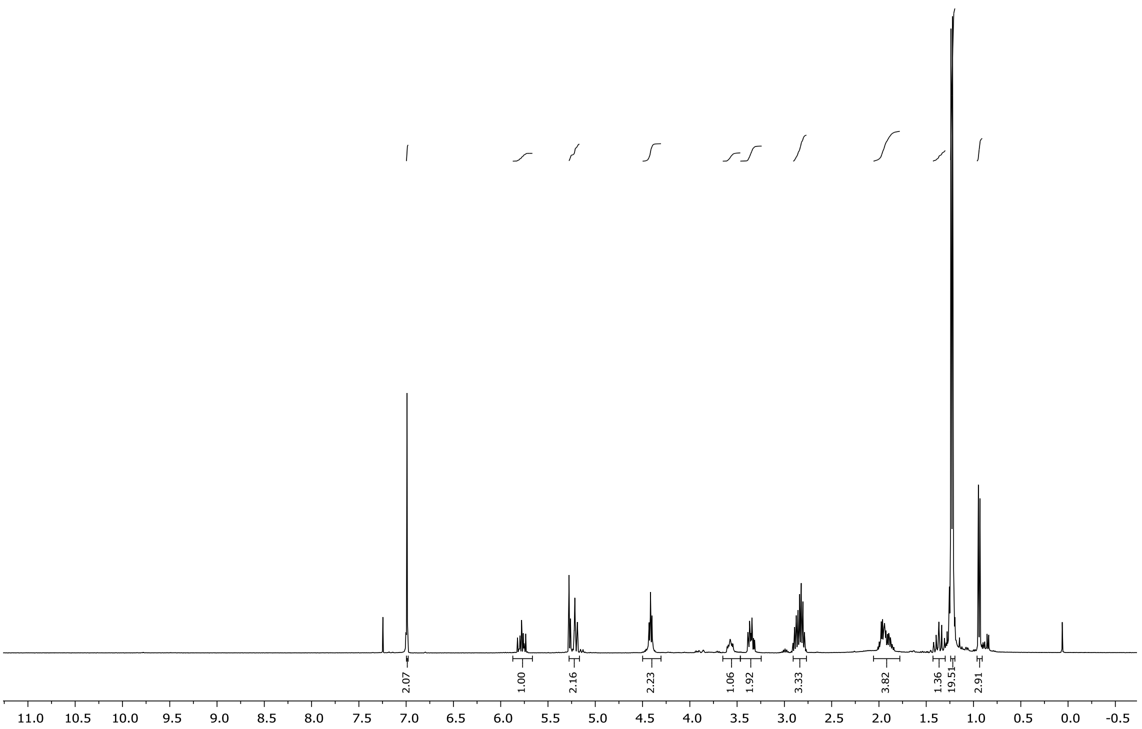
2.07
1.00
2.16
2.23
1.06
1.92
3.33
3.82
1.36
19.51
2.91
11.0 10.5 10.0 9.5 9.0 8.5 8.0 7.5 7.0 6.5 6.0 5.5 5.0 4.5 4.0 3.5 3.0 2.5 2.0 1.5 1.0 0.5 0.0 -0.5

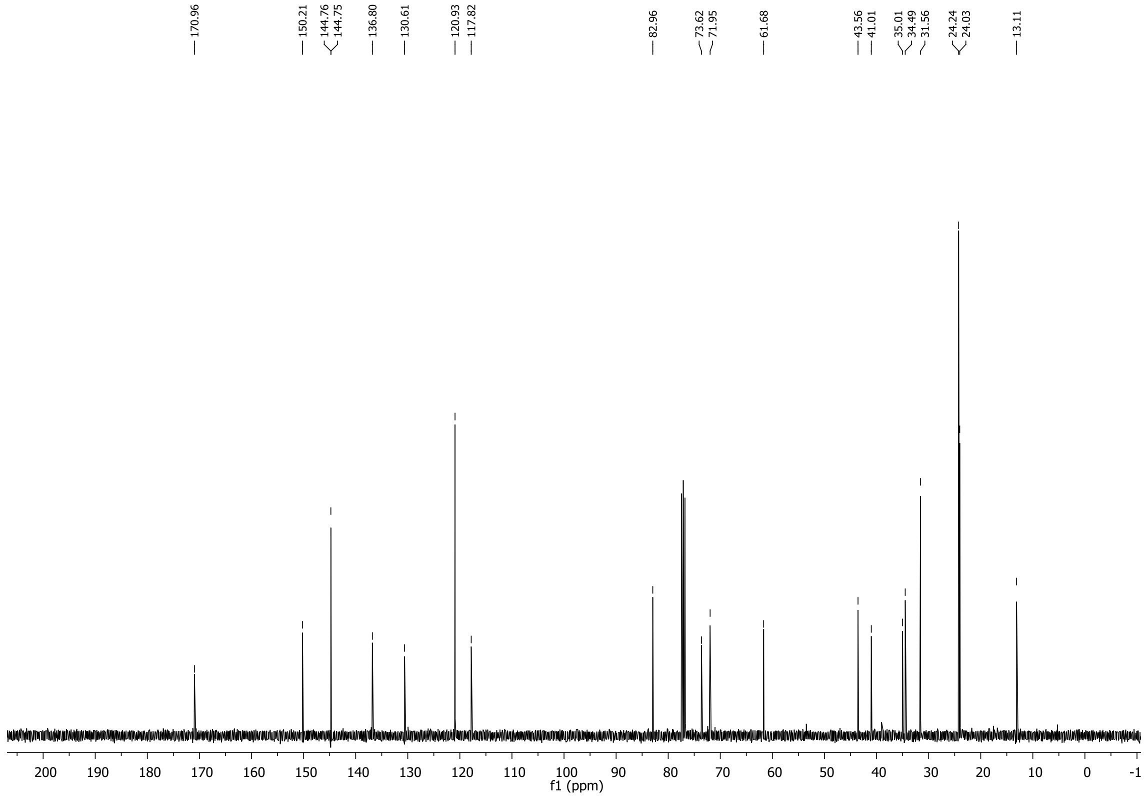
170.96
150.21
144.76
144.75
136.80
130.61
120.93
117.82
82.96
73.62
71.95
61.68
43.56
41.01
35.01
34.49
31.56
24.24
24.03
13.11
200 190 180 170 160 150 140 130 120 110 100 90 80 70 60 50 40 30 20 10 0 -1
f1 (ppm)

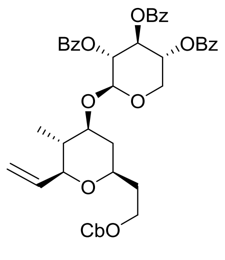
OBz
BzO
OBz
O
O
O
CbO

22a

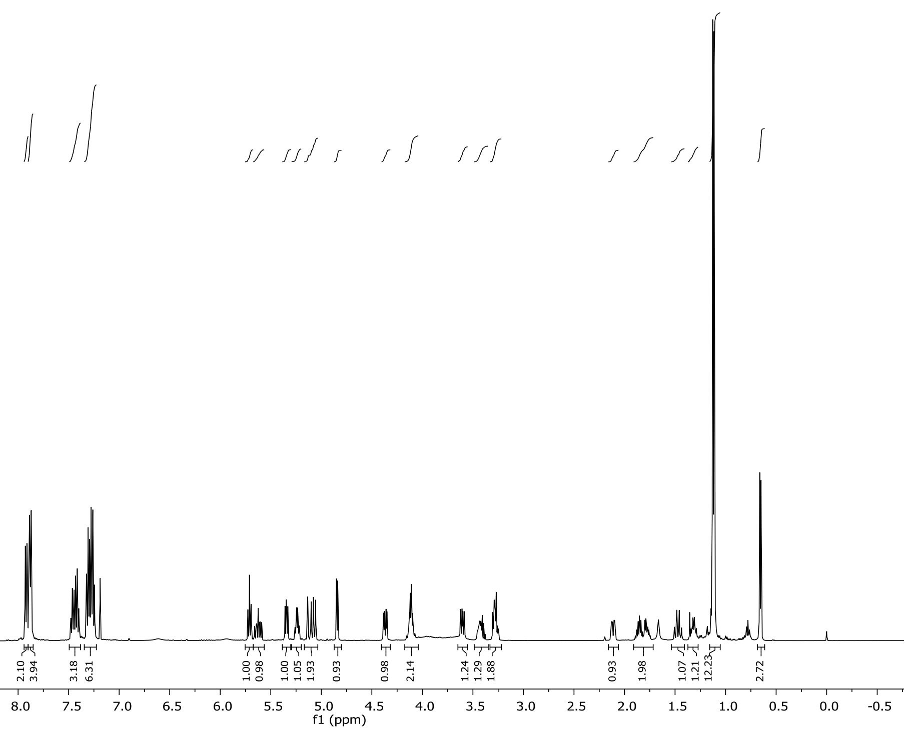
2.10
3.94
3.18
6.31
1.00
0.98
1.00
1.05
1.93
0.93
0.98
2.14
1.24
1.29
1.88
0.93
1.98
1.07
1.21
12.23
2.72
10.0 9.5 9.0 8.5 8.0 7.5 7.0 6.5 6.0 5.5 5.0 4.5 4.0 3.5 3.0 2.5 2.0 1.5 1.0 0.5 0.0 -0.5
f1 (ppm)

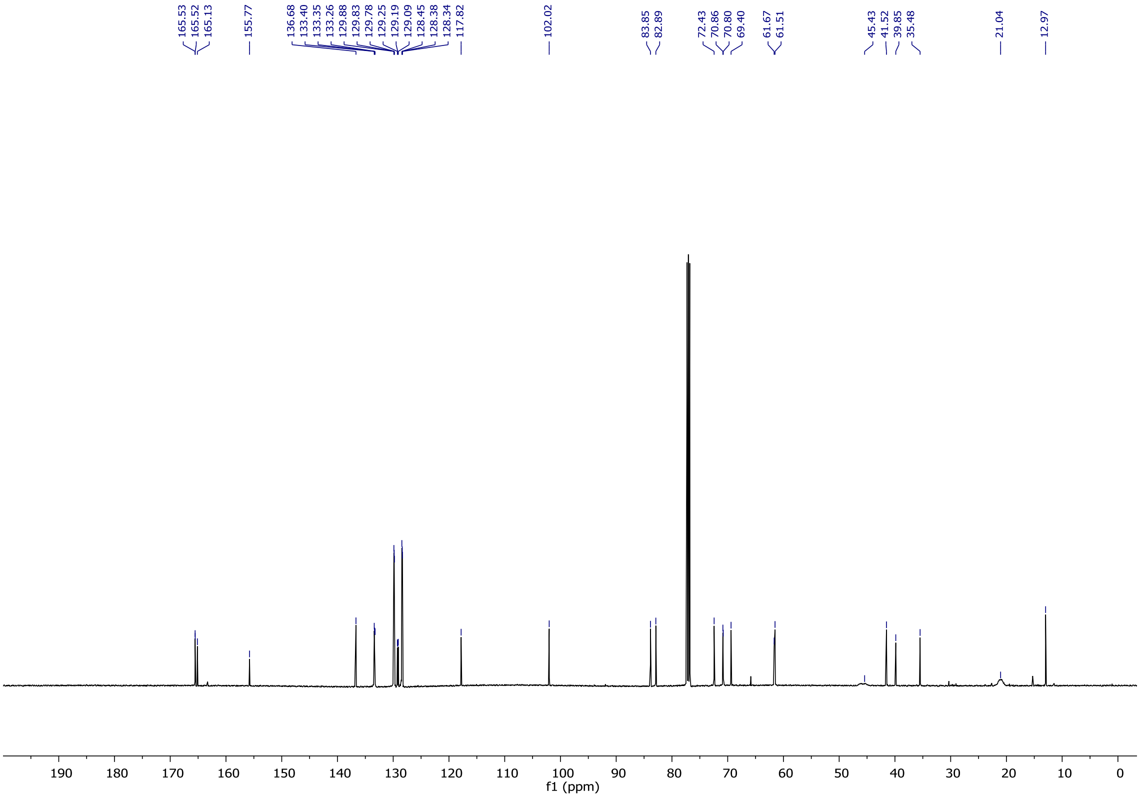
165.53
165.52
165.13
155.77
136.68
133.40
133.35
133.26
129.88
129.83
129.78
129.25
129.19
129.09
128.45
128.38
128.34
117.82
102.02
83.85
82.89
72.43
70.86
70.80
69.40
61.67
61.51
45.43
41.52
39.85
35.48
21.04
12.97
190 180 170 160 150 140 130 120 110 100 90 80 70 60 50 40 30 20 10 0
f1 (ppm)

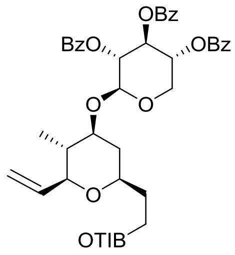
OBz
BzO
OBz
O
O
O
OTIB

22b

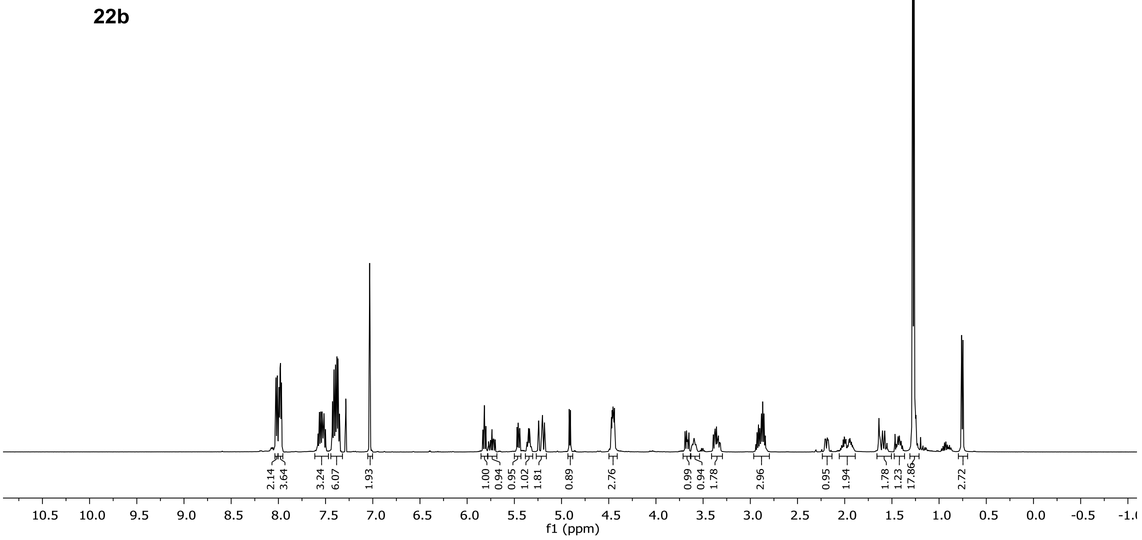
f1 (ppm)

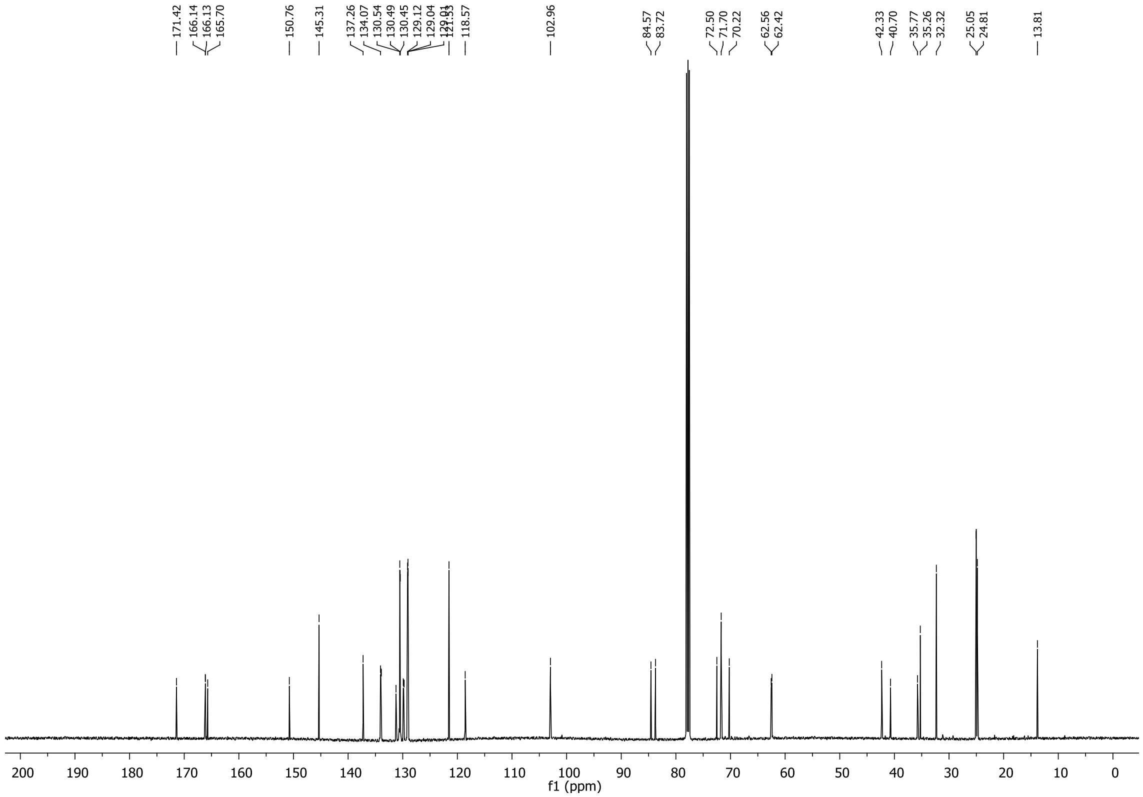
171.42
166.14
166.13
165.70
150.76
145.31
137.26
134.07
130.54
130.49
130.45
129.12
129.04
118.57
102.96
84.57
83.72
72.50
71.70
70.22
62.56
62.42
42.33
40.70
35.77
35.26
32.32
25.05
24.81
13.81
f1 (ppm)

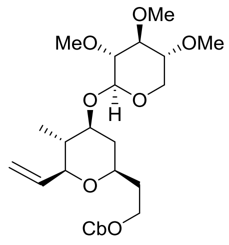

OMe
MeO
OMe
O
H
O
O
CbO

3a

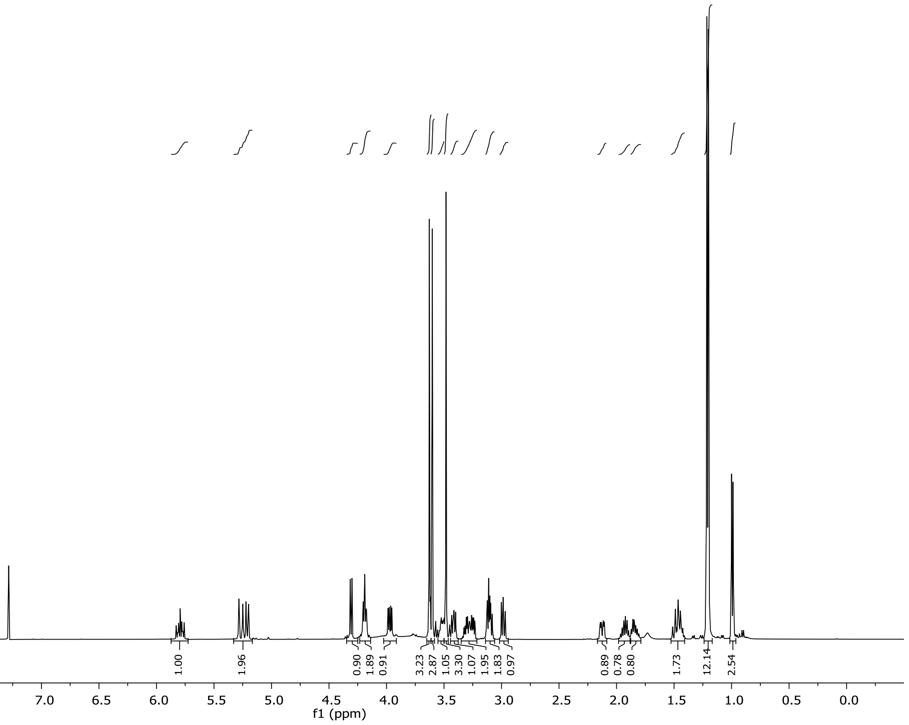

1.00
1.96
0.90
1.89
0.91
3.23
2.87
1.05
3.30
1.07
1.95
1.83
0.97
0.89
0.78
0.80
1.73
12.14
2.54
9.0 8.5 8.0 7.5 7.0 6.5 6.0 5.5 5.0 4.5 4.0 3.5 3.0 2.5 2.0 1.5 1.0 0.5 0.0
f1 (ppm)

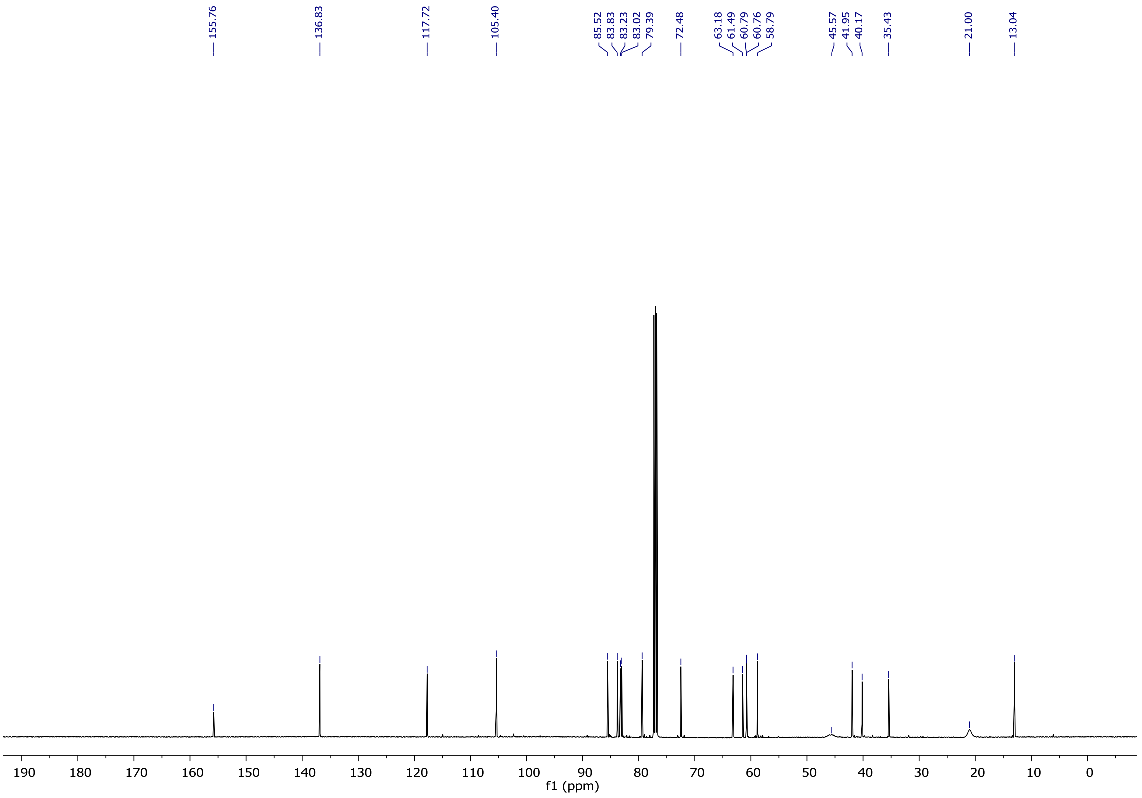

155.76
136.83
117.72
105.40
85.52
83.83
83.23
83.02
79.39
72.48
63.18
61.49
60.79
60.76
58.79
45.57
41.95
40.17
35.43
21.00
13.04
190 180 170 160 150 140 130 120 110 100 90 80 70 60 50 40 30 20 10 0
f1 (ppm)

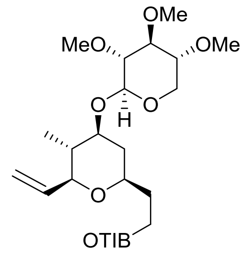
OMe
MeO
OMe
O
H
O
O
OTIB

3b

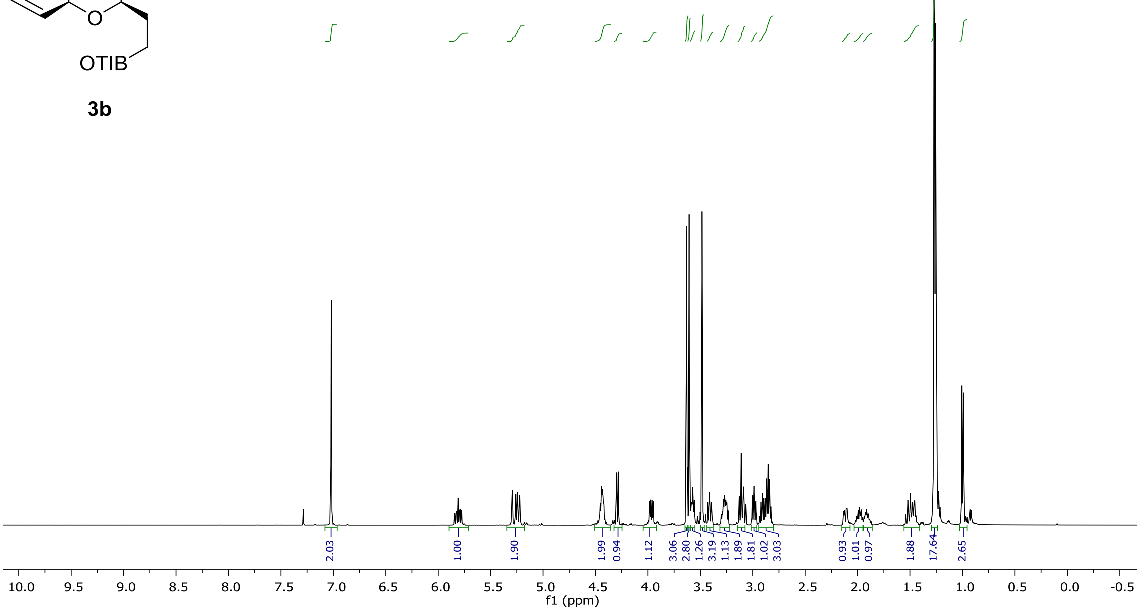
2.03
1.00
1.90
1.99
0.94
1.12
3.06
2.80
1.26
3.19
1.13
1.89
1.81
1.02
3.03
0.93
1.01
0.97
1.88
17.64
2.65
10.0 9.5 9.0 8.5 8.0 7.5 7.0 6.5 6.0 5.5 5.0 4.5 4.0 3.5 3.0 2.5 2.0 1.5 1.0 0.5 0.0 -0.5
f1 (ppm)

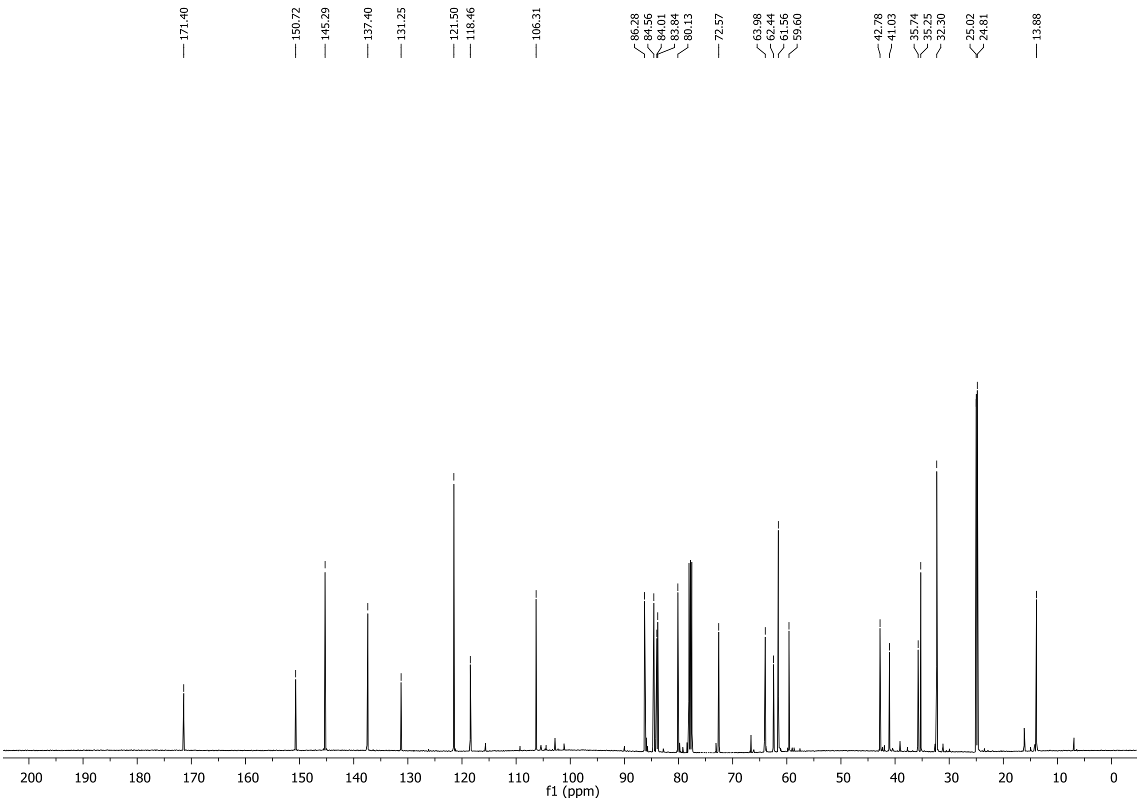
171.40
150.72
145.29
137.40
131.25
121.50
118.46
106.31
86.28
84.56
84.01
83.84
80.13
72.57
63.98
62.44
61.56
59.60
42.78
41.03
35.74
35.25
32.30
25.02
24.81
13.88
200 190 180 170 160 150 140 130 120 110 100 90 80 70 60 50 40 30 20 10 0
f1 (ppm)

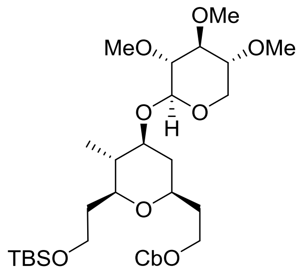
OMe
MeO
OMe
O
H
O
O
TBSO
CbO

14a

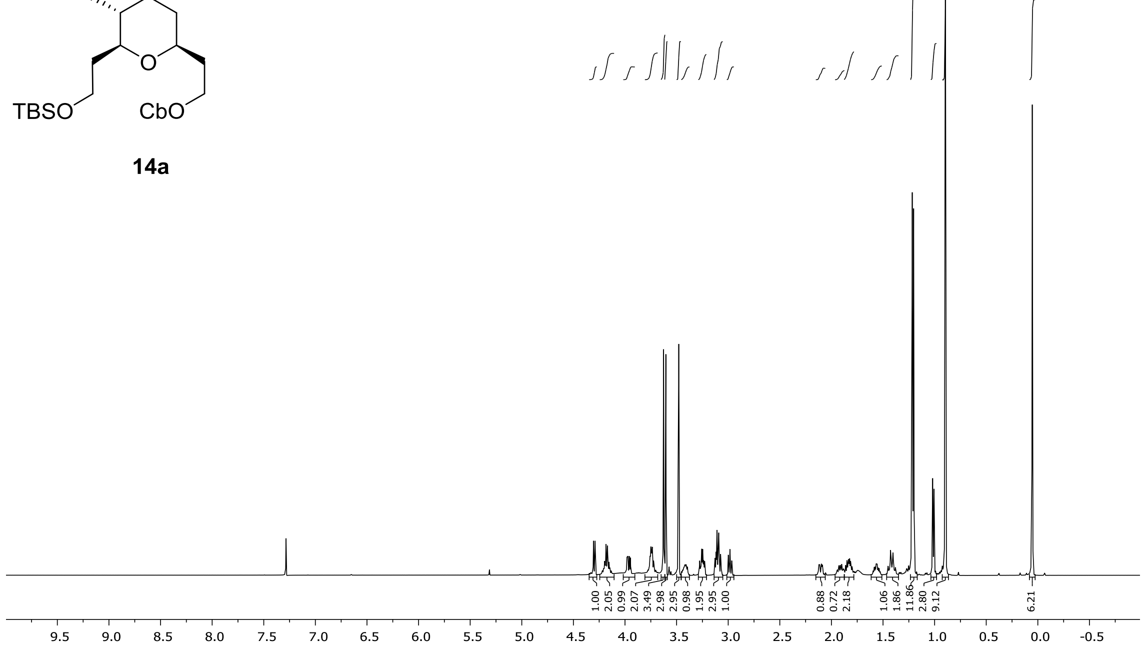
1.00
2.05
0.99
2.07
3.49
2.98
2.95
0.98
1.95
2.95
1.00
0.88
0.72
2.18
1.06
1.86
11.86
2.80
9.12
6.21
9.5 9.0 8.5 8.0 7.5 7.0 6.5 6.0 5.5 5.0 4.5 4.0 3.5 3.0 2.5 2.0 1.5 1.0 0.5 0.0 -0.5

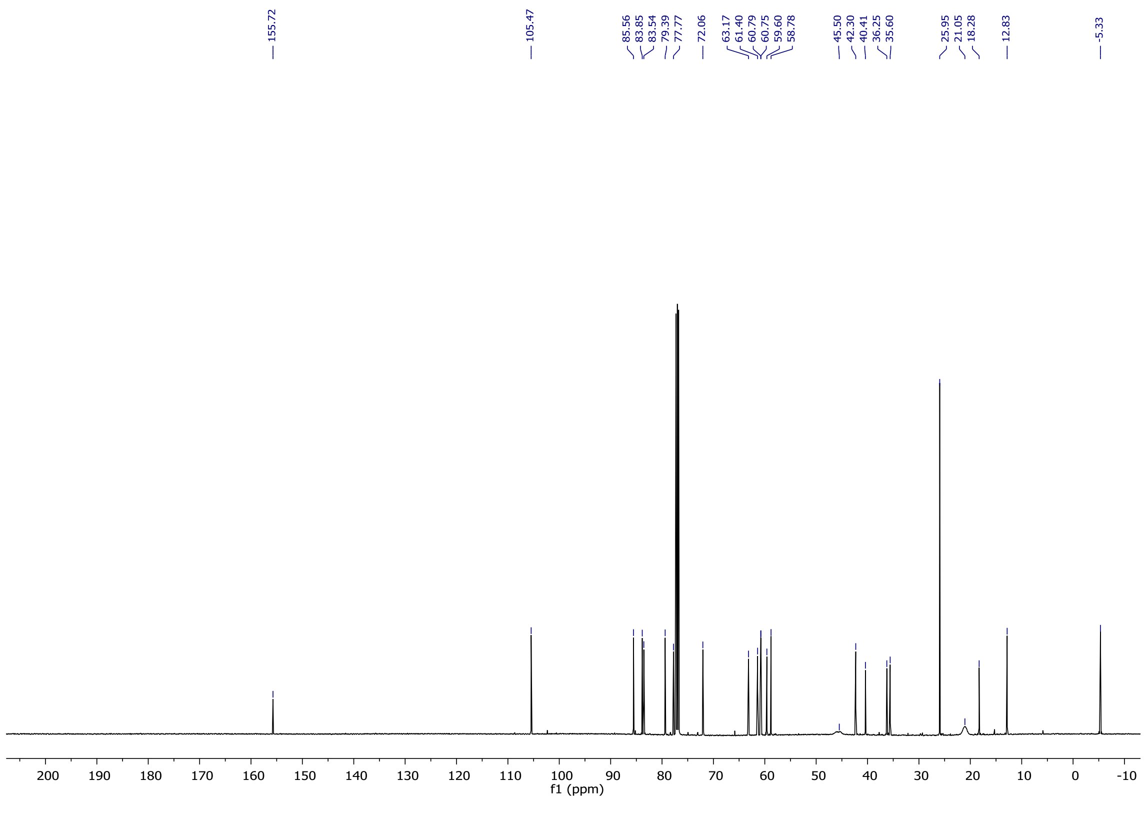
155.72
105.47
85.56
83.85
83.54
79.39
77.77
72.06
63.17
61.40
60.79
60.75
59.60
58.78
45.50
42.30
40.41
36.25
35.60
25.95
21.05
18.28
12.83
-5.33
200 190 180 170 160 150 140 130 120 110 100 90 80 70 60 50 40 30 20 10 0 -10
f1 (ppm)

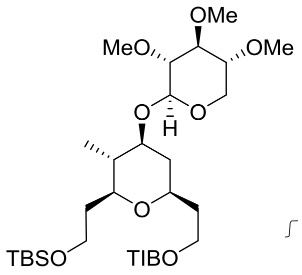

14b

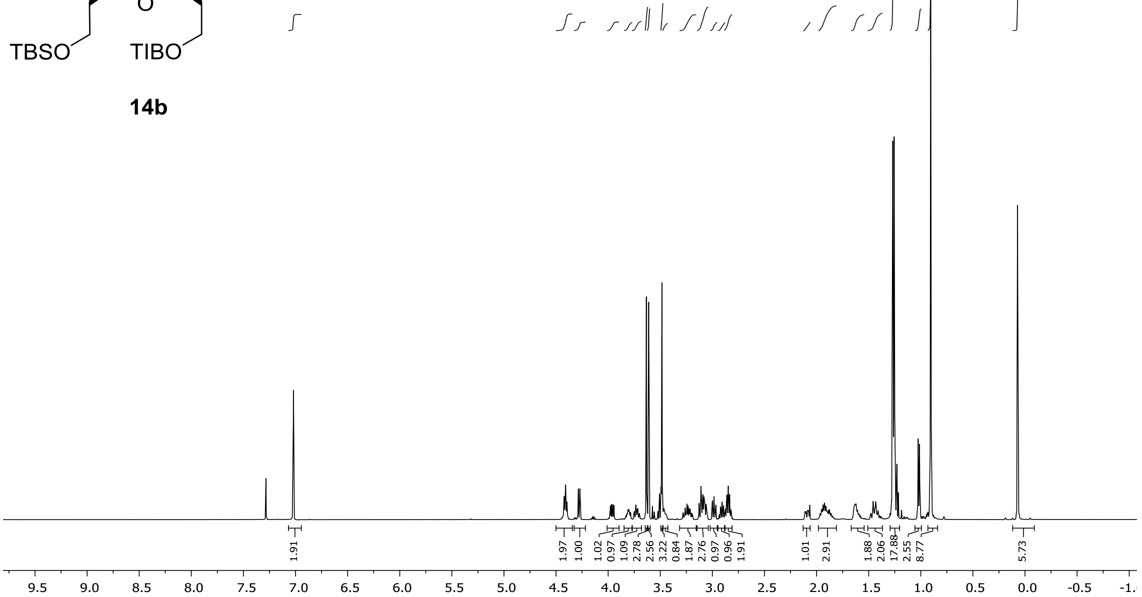

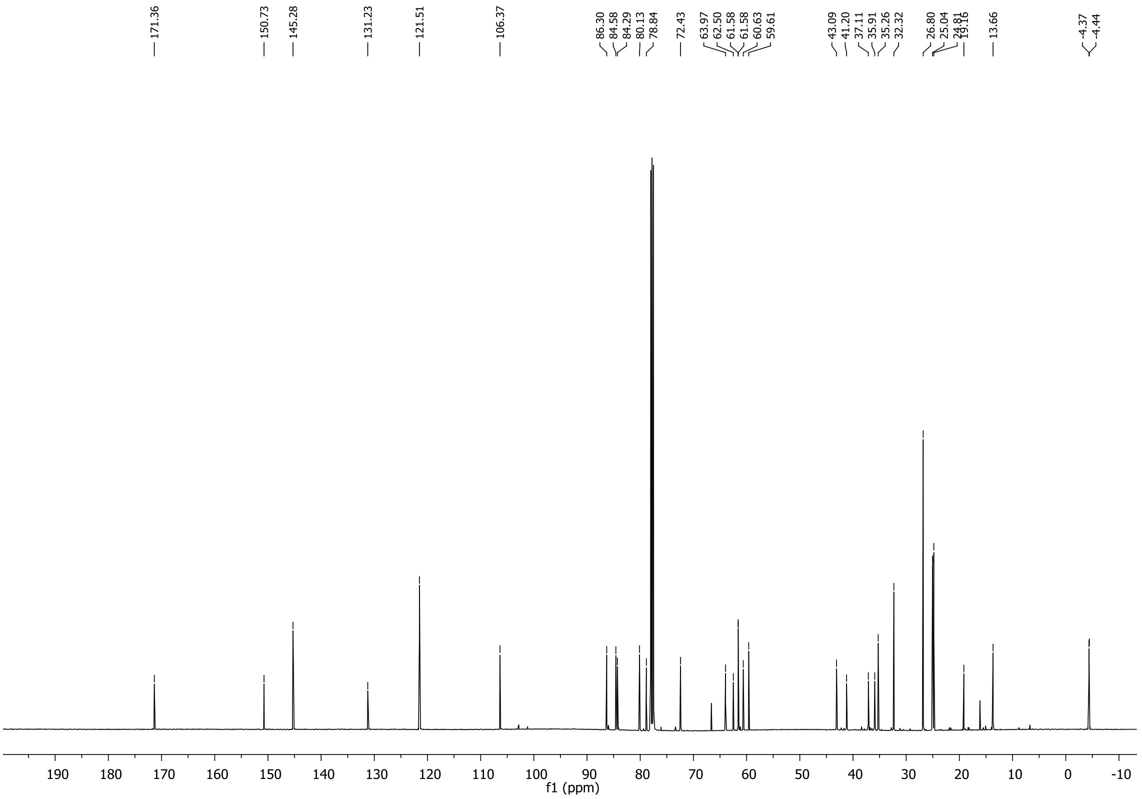

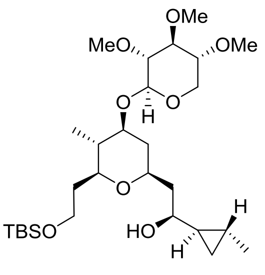
OMe
MeO
OMe
O
H
O
O
TBSO
HO
H
H

15a

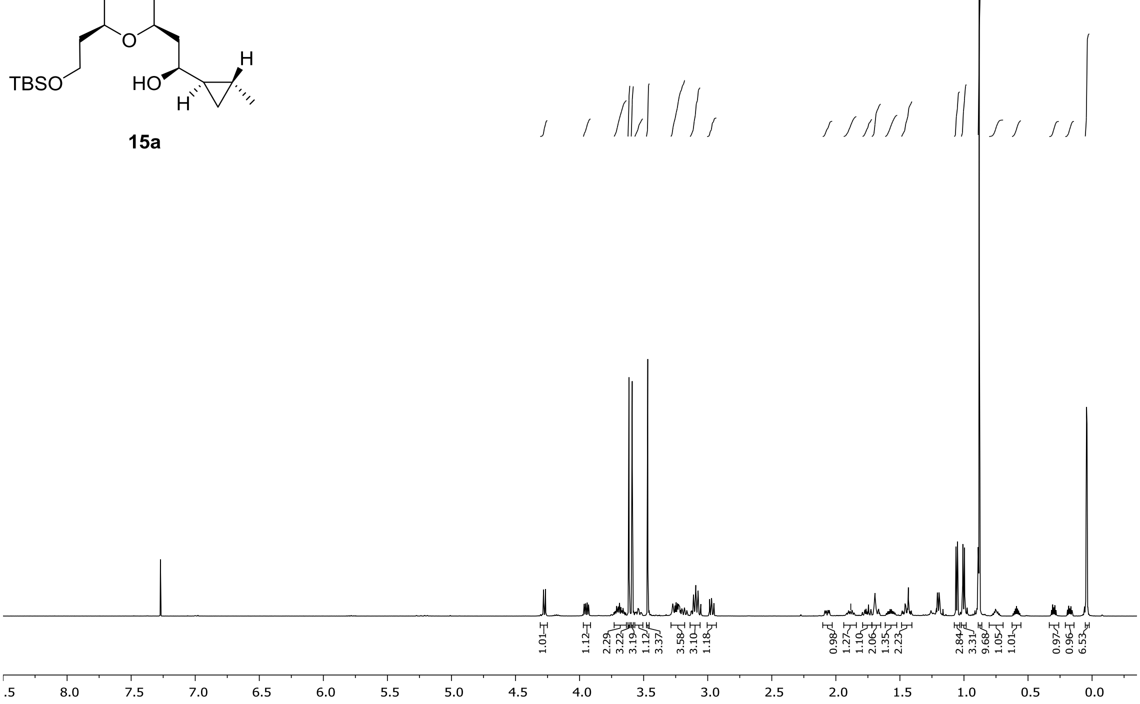

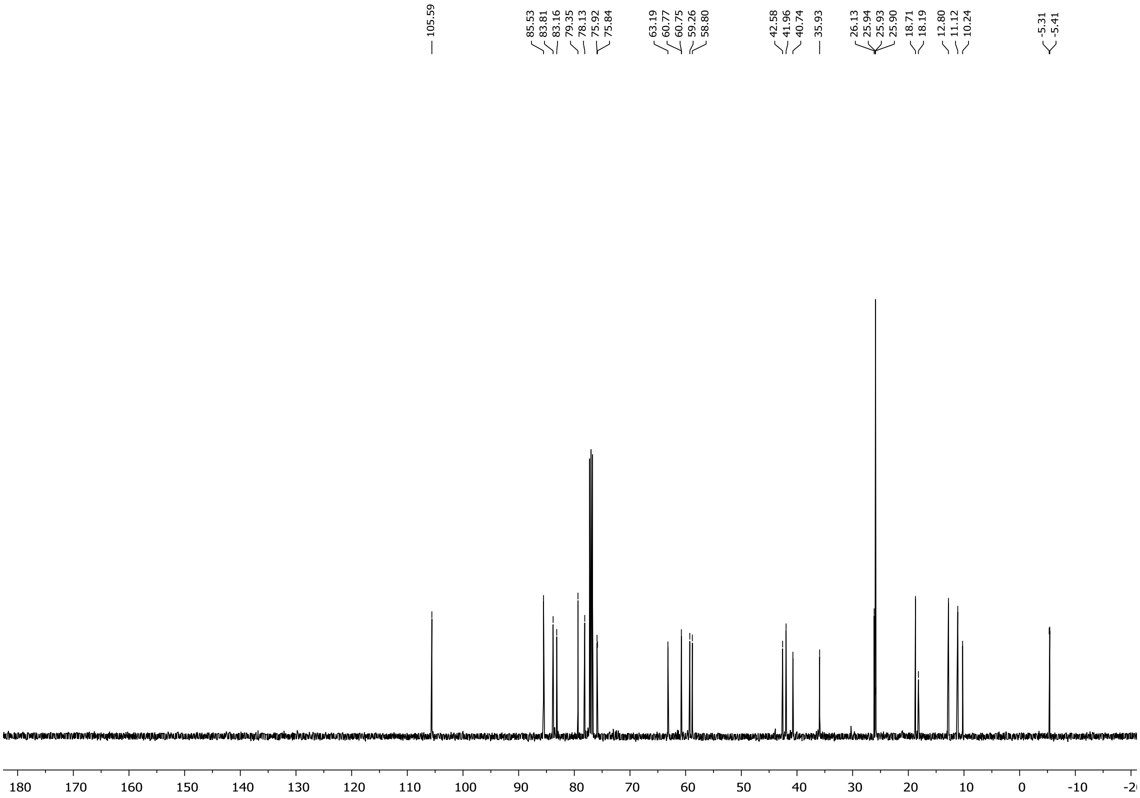
105.59
85.53
83.81
83.16
79.35
78.13
75.92
75.84
63.19
60.77
60.75
59.26
58.80
42.58
41.96
40.74
35.93
26.13
25.94
25.93
25.90
18.71
18.19
12.80
11.12
10.24
-5.31
-5.41

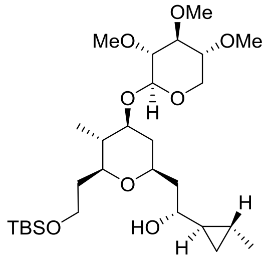
OMe
MeO
OMe
O
H
O
O
TBSO
HO
H
H

15b

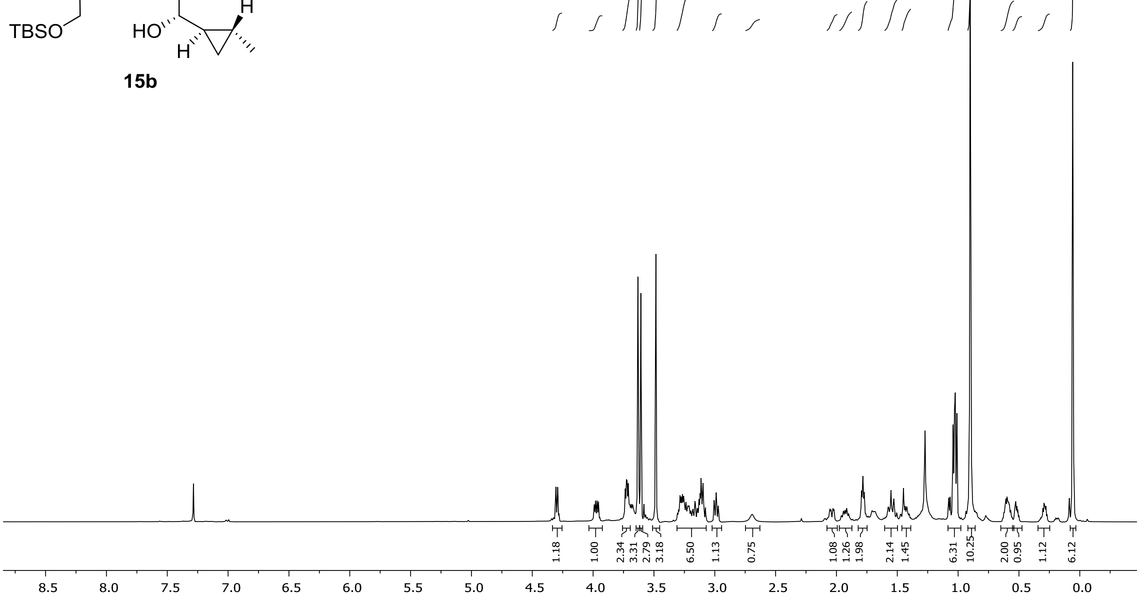
1.18
1.00
2.34
3.31
2.79
3.18
6.50
1.13
0.75
1.08
1.26
1.98
2.14
1.45
6.31
10.25
2.00
0.95
1.12
6.12
8.5 8.0 7.5 7.0 6.5 6.0 5.5 5.0 4.5 4.0 3.5 3.0 2.5 2.0 1.5 1.0 0.5 0.0

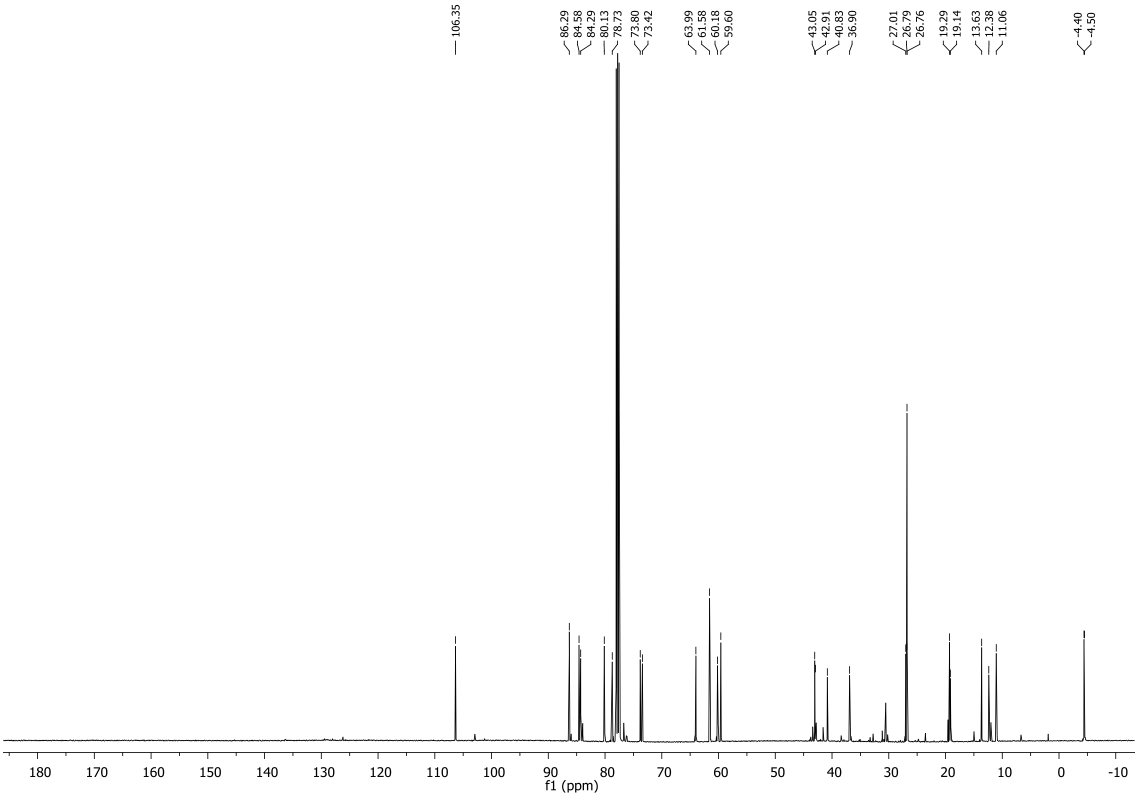
106.35
86.29
84.58
84.29
80.13
78.73
73.80
73.42
63.99
61.58
60.18
59.60
43.05
42.91
40.83
36.90
27.01
26.79
26.76
19.29
19.14
13.63
12.38
11.06
-4.40
-4.50
180 170 160 150 140 130 120 110 100 90 80 70 60 50 40 30 20 10 0 -10
f1 (ppm)

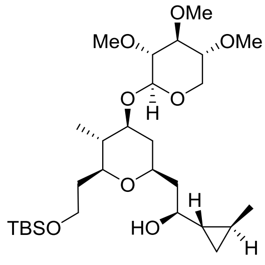
OMe
MeO
OMe
O
H
O
O
TBSO
HO
H
H

15c

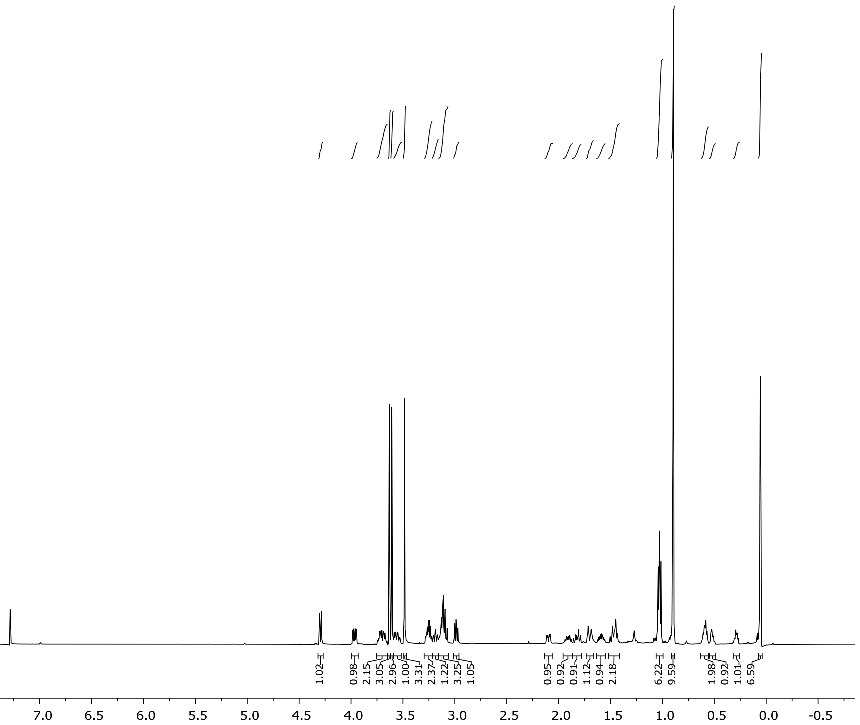
1.02
0.98
2.15
3.05
2.96
1.00
3.31
2.37
1.22
3.25
1.05
0.95
0.92
0.91
1.12
0.94
2.18
6.22
9.59
1.98
0.92
1.01
6.59

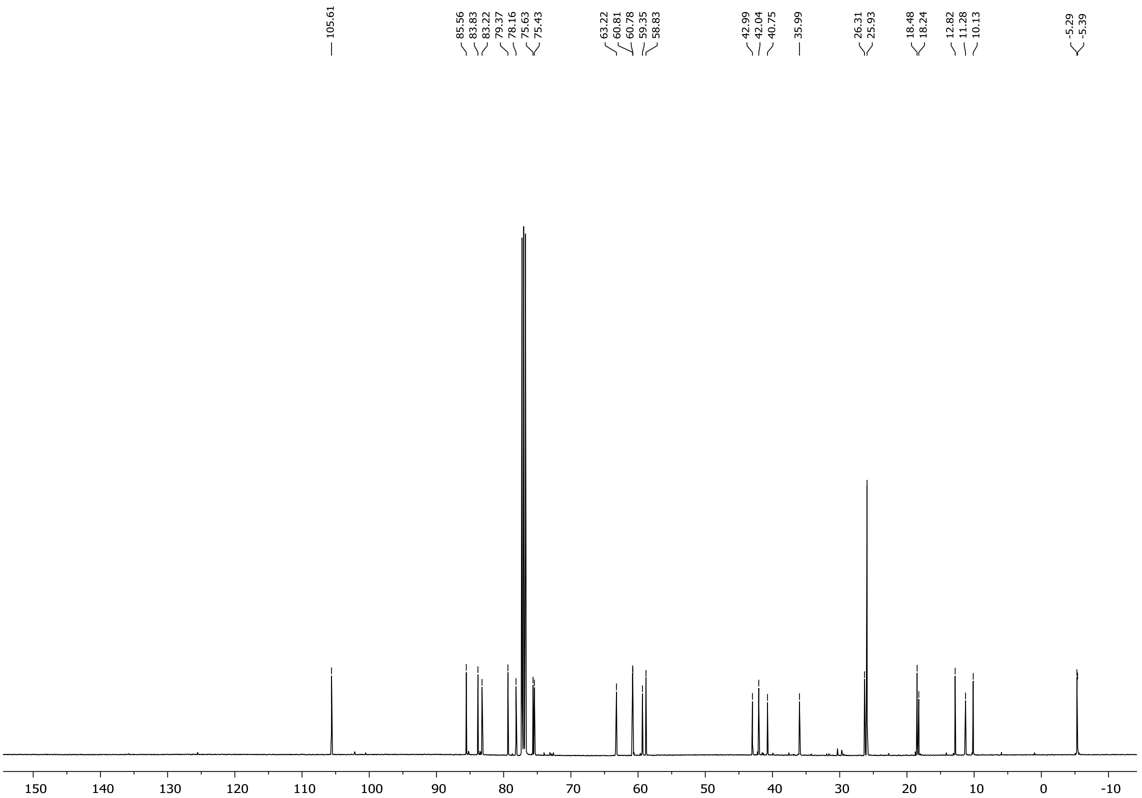
105.61
85.56
83.83
83.22
79.37
78.16
75.63
75.43
63.22
60.81
60.78
59.35
58.83
42.99
42.04
40.75
35.99
26.31
25.93
18.48
18.24
12.82
11.28
10.13
-5.29
-5.39

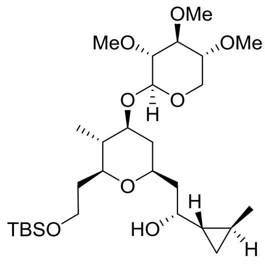
OMe
MeO
OMe
O
H
O
O
TBSO
HO
H
H

15d

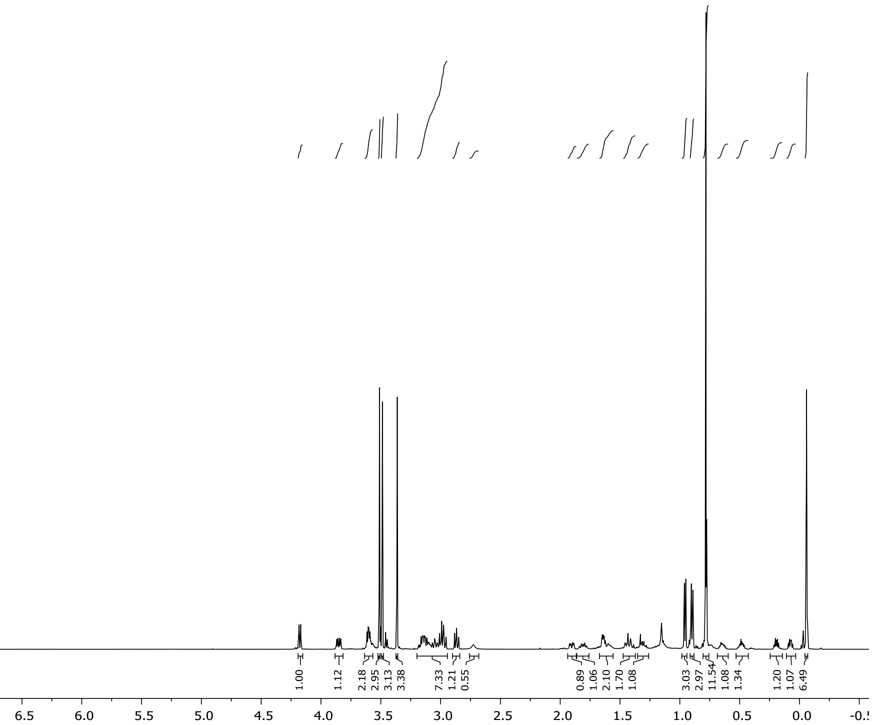
1.00
1.12
2.18
2.95
3.13
3.38
7.33
1.21
0.55
0.89
1.06
2.10
1.70
1.08
3.03
2.97
11.54
1.08
1.34
1.20
1.07
6.49
8.5 8.0 7.5 7.0 6.5 6.0 5.5 5.0 4.5 4.0 3.5 3.0 2.5 2.0 1.5 1.0 0.5 0.0

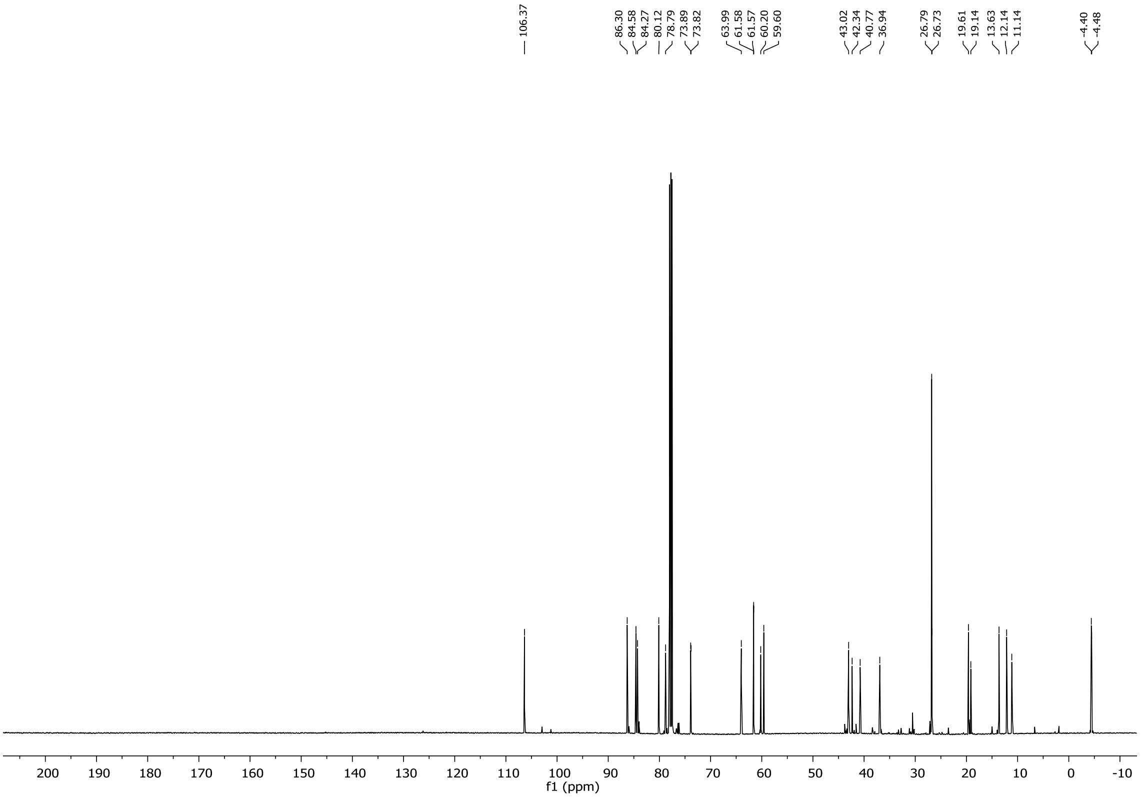
106.37
86.30
84.58
84.27
80.12
78.79
73.89
73.82
63.99
61.58
61.57
60.20
59.60
43.02
42.34
40.77
36.94
26.79
26.73
19.61
19.14
13.63
12.14
11.14
-4.40
-4.48
200 190 180 170 160 150 140 130 120 110 100 90 80 70 60 50 40 30 20 10 0 -10
f1 (ppm)

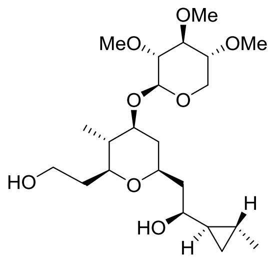
OMe
MeO
OMe
O
O
O
HO
HO
H
H

23

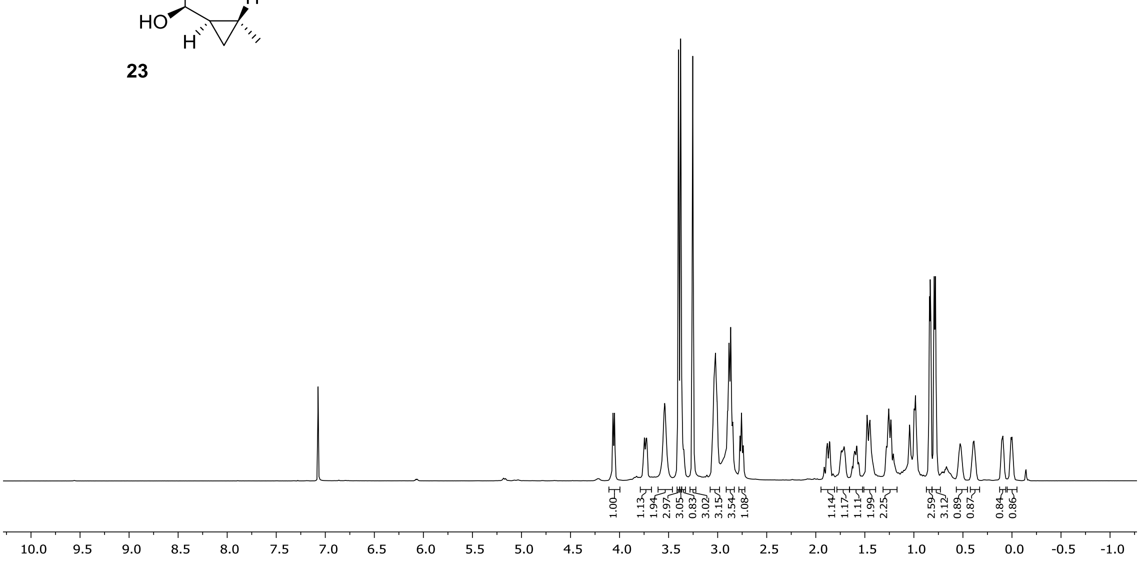
1.00
1.13
1.94
2.97
3.05
0.83
3.02
3.15
3.54
1.08
1.14
1.17
1.11
1.99
2.25
2.59
3.12
0.89
0.87
0.84
0.86
10.0 9.5 9.0 8.5 8.0 7.5 7.0 6.5 6.0 5.5 5.0 4.5 4.0 3.5 3.0 2.5 2.0 1.5 1.0 0.5 0.0 -0.5 -1.0

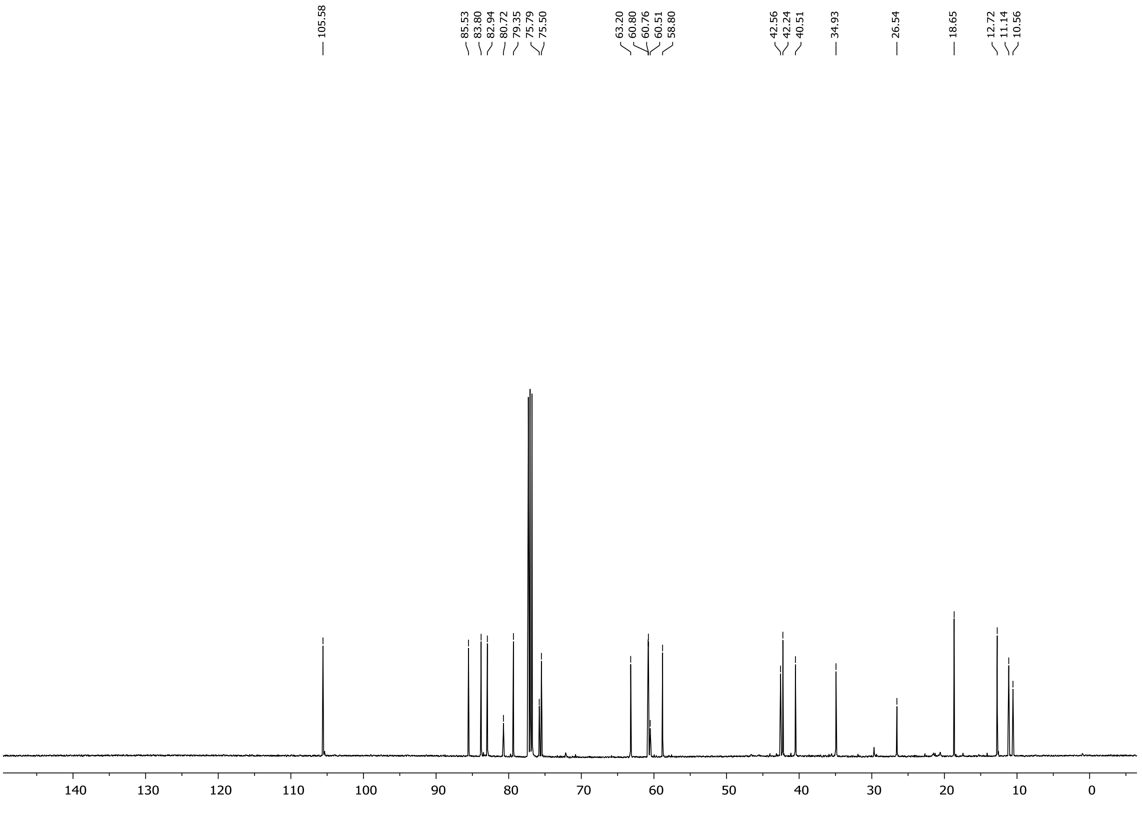
105.58
85.53
83.80
82.94
80.72
79.35
75.79
75.50
63.20
60.80
60.76
60.51
58.80
42.56
42.24
40.51
34.93
26.54
18.65
12.72
11.14
10.56
140 130 120 110 100 90 80 70 60 50 40 30 20 10 0

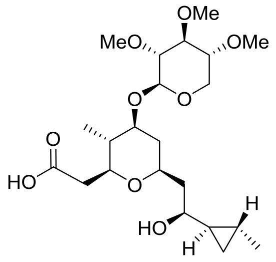
OMe
MeO
OMe
O
O
O
HO
O
HO
H
H

2

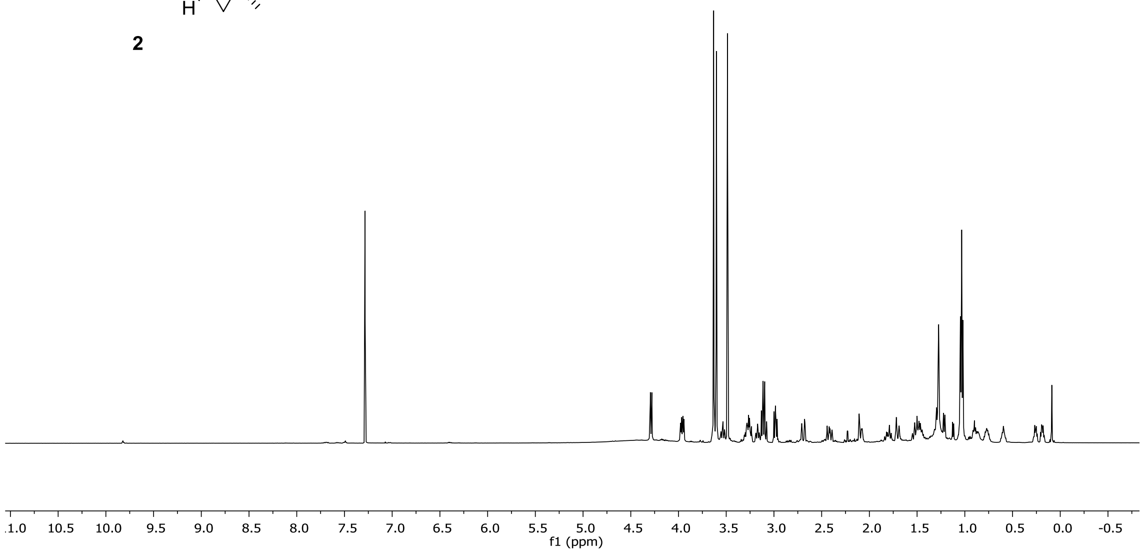
f1 (ppm)

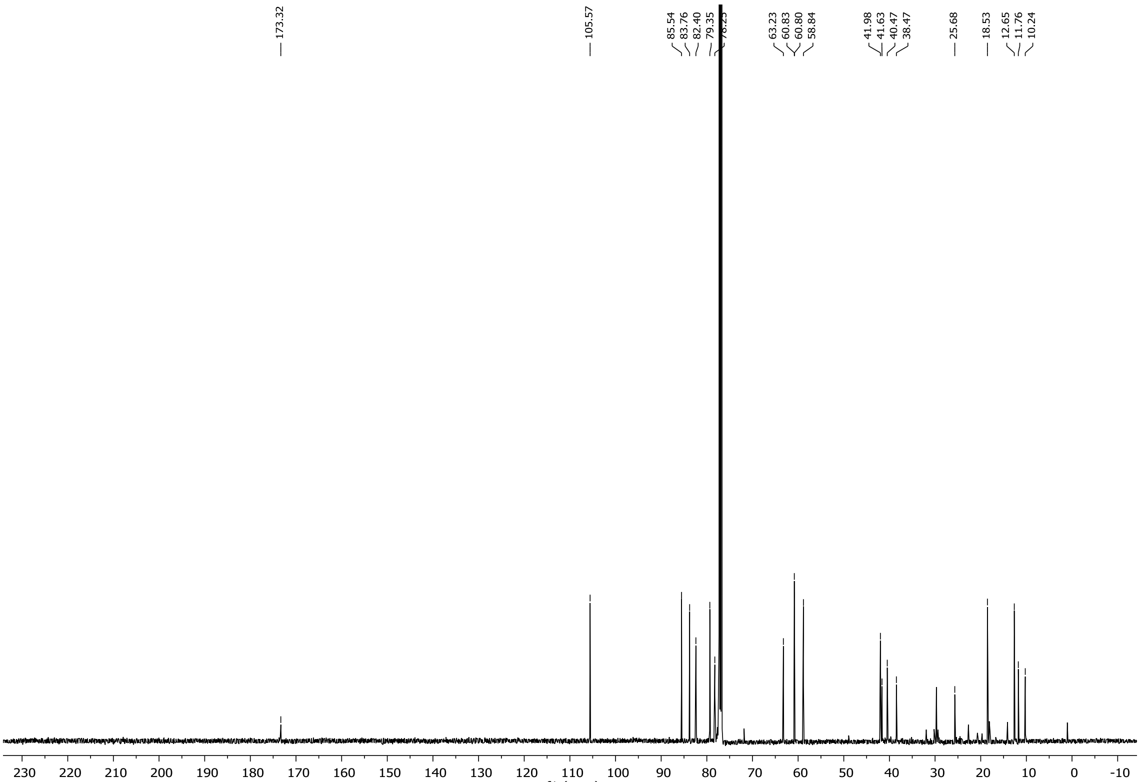
173.32
105.57
85.54
83.76
82.40
79.35
63.23
60.83
60.80
58.84
41.98
41.63
40.47
38.47
25.68
18.53
12.65
11.76
10.24
f1 (ppm)

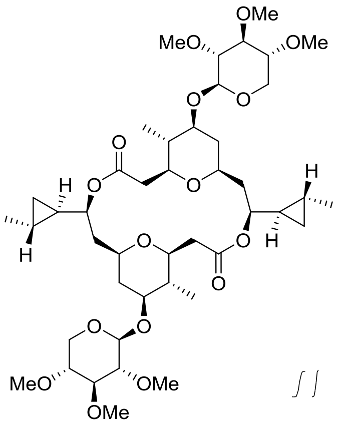
OMe
MeO
OMe
O
H
O
O
O
H
O
O
O
O
MeO
OMe
OMe

1

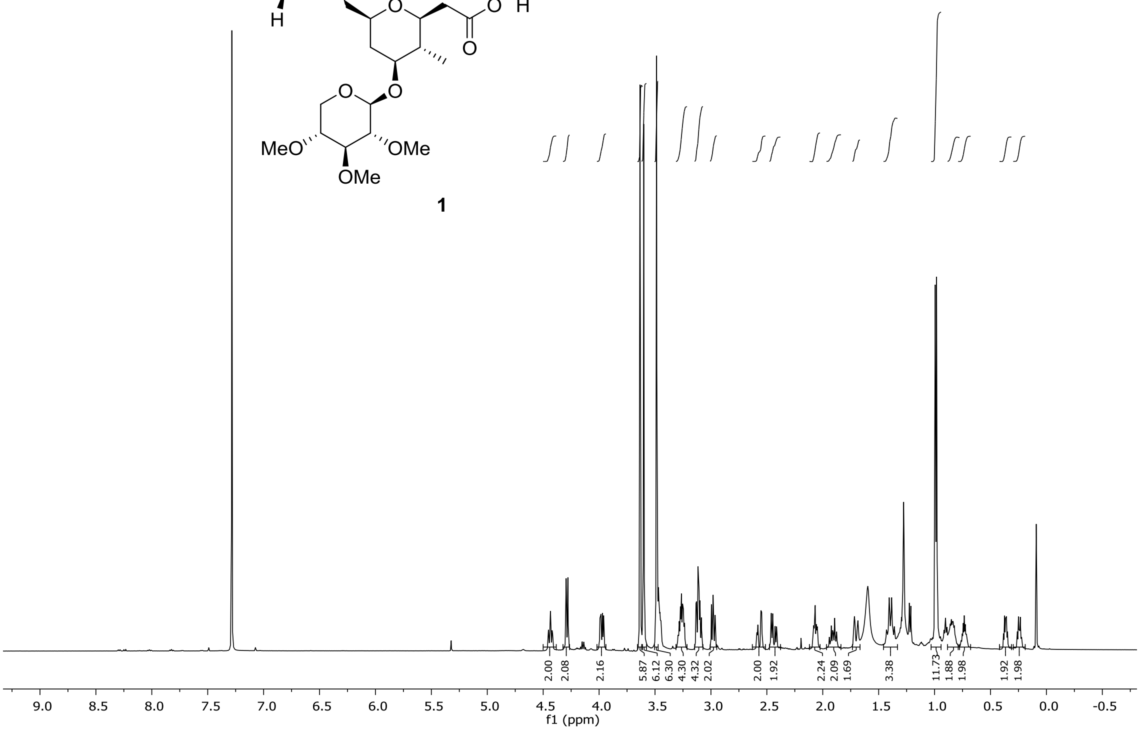
2.00
2.08
2.16
5.87
6.12
6.30
4.30
4.32
2.02
2.00
1.92
2.24
2.09
1.69
3.38
11.73
1.88
1.98
1.92
1.98
9.0 8.5 8.0 7.5 7.0 6.5 6.0 5.5 5.0 4.5 4.0 3.5 3.0 2.5 2.0 1.5 1.0 0.5 0.0 -0.5
f1 (ppm)

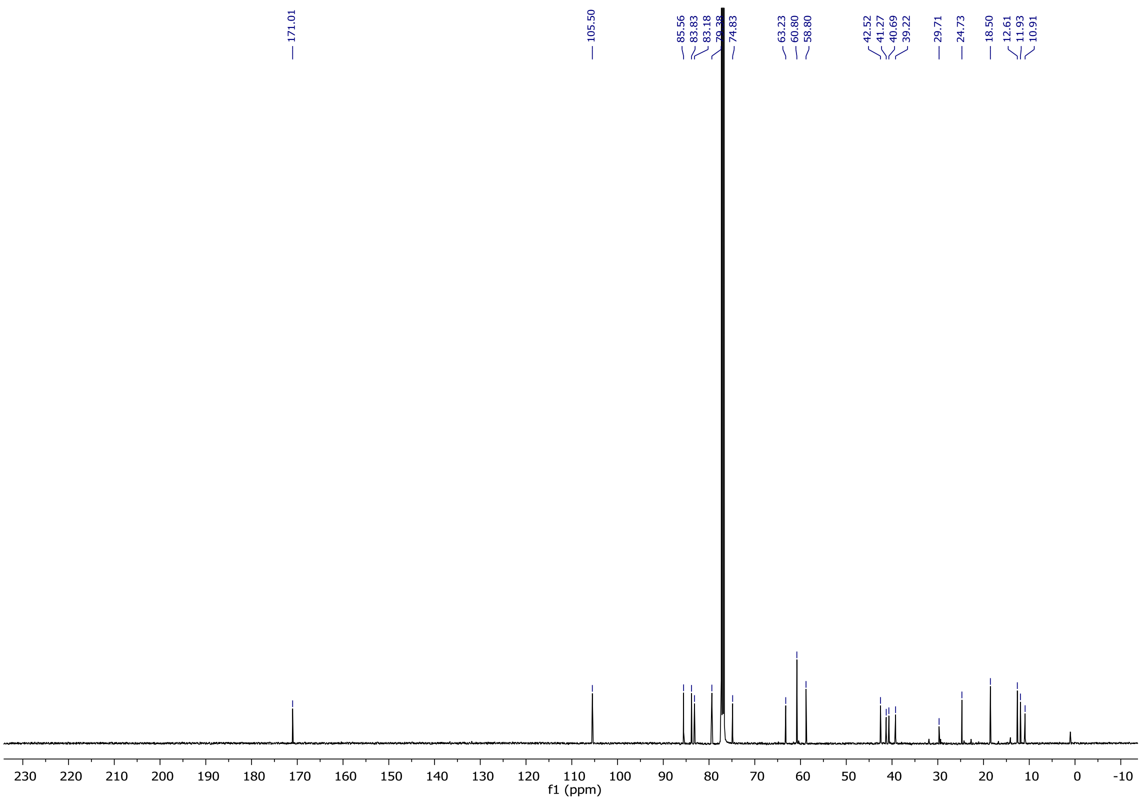
171.01
105.50
85.56
83.83
83.18
74.83
63.23
60.80
58.80
42.52
41.27
40.69
39.22
29.71
24.73
18.50
12.61
11.93
10.91
230 220 210 200 190 180 170 160 150 140 130 120 110 100 90 80 70 60 50 40 30 20 10 0 -10
f1 (ppm)

7. HPLC and GC analysis

Determination of dr for 8e by GC analysis

The dr of **8e** could not be determined by ^{1}H NMR analysis. The dr was determined by GC analysis.

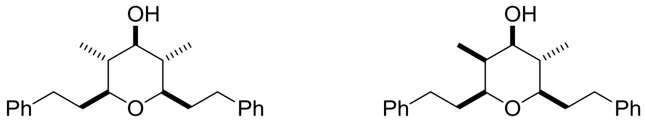

8e **9e**

GC (HP-5 column, 1.5 mL/min, 17.6 psi, ramp 10.0 °C/min from 70 °C to 100 °C then ramp 5.0 °C/min to 300 °C): t_R= 29.230 (major diastereomer **8e**), 29.853 (minor diastereomer **9e**), dr= 90:10.

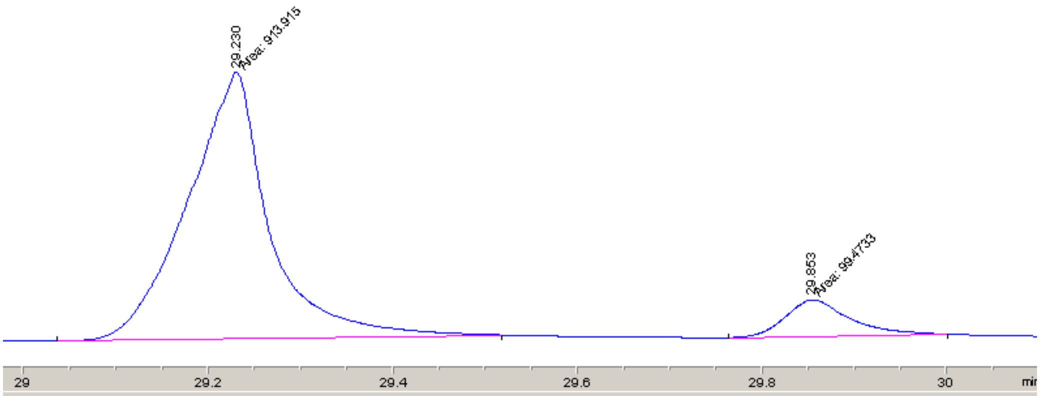

#	Time	Area	Height	Width	Area%	Symmetry
1	29.23	913.9	148.8	0.1024	90.184	1.268
2	29.853	99.5	20.6	0.0805	9.816	0.627

Derivatization of (R)-6a to determine er value by HPLC analysis

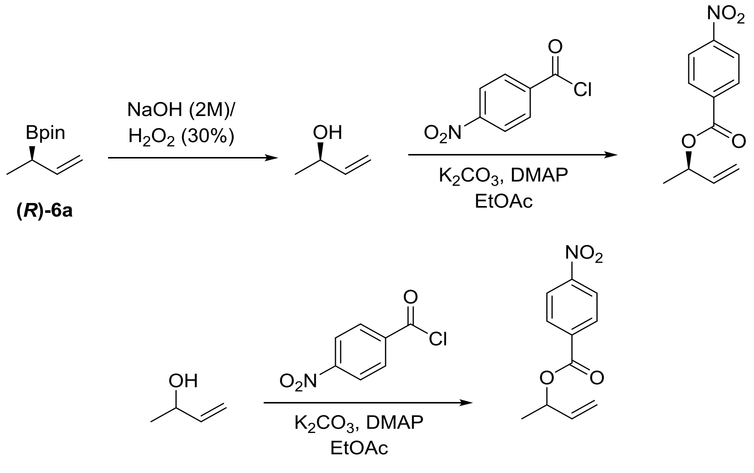

Boronic ester (*R*)-**6a** (100 mg, 0.549 mmol) was diluted in Et_2O (1 mL) and cooled to 0°C. A solution of NaOH (2M) and H_2O_2 (30%) (2:1, v/v, 2.2 mL) was added dropwise. This mixture was stirred for 2 h at rt and quenched with 2M NaOH. The layers were separated and the aqueous phase was extracted with Et_2O (x2). The combined organic layers were dried over $MgSO_4$ and

evaporated under reduced pressure at 0°C. The crude was submitted to the next step without further purification.

To a stirred solution of the crude alcohol in EtOAc (0.15 M) were added 4-nitrobenzoyl chloride (509 mg, 2.745 mmol, 5 equiv), anhydrous K_2CO_3 (378 mg, 2.745 mmol, 5 equiv) and N, N-dimethylaminopyridine (3 mg, 0.027 mmol, 0.05 equiv). The reaction was stirred at room temperature for 12 h. The mixture was filtered by suction using a sintered glass funnel and the solid residue was washed with EtOAc. The organic layer was evaporated under reduced pressure and the residue was purified by column chromatography eluting 5% EtOAc: hexane mixture. The final product was obtained as yellow solid. Yield was not determined.

Chiral HPLC (Chiralpak AD-H with guard, 0.5 ml/min, 0.5% IPA: hexane): t_R= 44.607 (major), 48.508 (minor), *er*= 96: 4

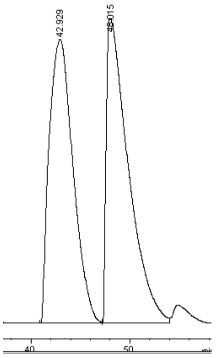

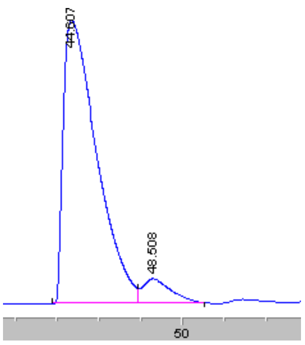

	Time	Area	Area%
Major	44.607	45007.4	95.901
Minor	48.508	1926.19	4.099

Derivatization of 4 and ent-4 to determine er value by HPLC analysis

Optical rotation values for **4** and *ent*-**4**

Compound **4: $[\alpha]_D^{22}$**= –29 (*c* 1, $CHCl_3$).

Compound *ent*- **4: $[\alpha]_D^{22}$**= +40 (*c* 1, $CHCl_3$)

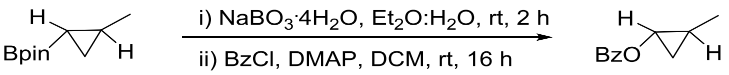

The corresponding boronic ester (100 mg, 0.552 mmol, 1 equiv.) was dissolved in Et_2O: H_2O (1:1, 2 mL) and $NaBO_3 \cdot 4H_2O$ was added. The reaction was stirred for 2 h at rt. Phases were separated and aqueous phase was extracted with Et_2O (x2), dried and evaporated under reduced pressure without heating. The crude was dissolved in DCM (2 mL) and DMAP (337 mg, 2.76 mmol, 5 equiv.), and BzCl (0.32 mL, 2.76 mmol, 5 equiv.) was added. The reaction was stirred overnight at rt. The mixture was diluted with DCM and washed with brine (x2), dried over $MgSO_4$ and

evaporated. The crude was purified by column chromatography (SiO_2, petroleum: EtOAc, 9:1). The final product was obtained as white solid. Yield was not determine.

Chiral HPLC (Chiralpak AS-H with guard, 0.5 ml/min, hexane)

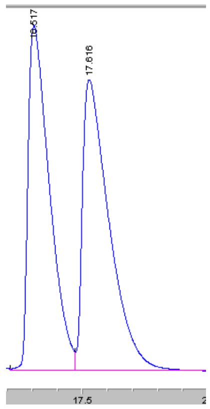

rac-**4**

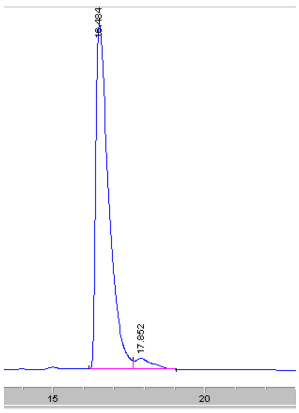

4

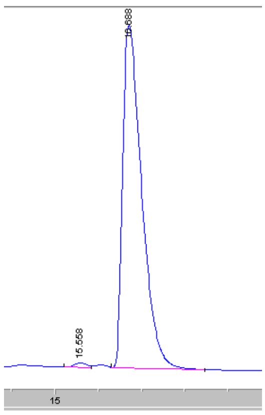

ent-**4**

For compound **4**: t_R= 16.484 (major), 17.852 (minor), *er*= 96: 4

	Time	Area	Area%
Major	16.484	9146.7	96.135
Minor	17.852	367.8	3.865

For compound *ent*-**4**: t_R= 15.558 (minor), 16.688 (major), *er*= 99: 1

	Time	Area	Area%
Minor	15.558	65.7	1.031
Major	16.688	6303.2	98.969

For *ent*-**4** doped with *rac*-**4**: t_R= 15.775 (minor), 16.235 (major), *er*= 96: 4

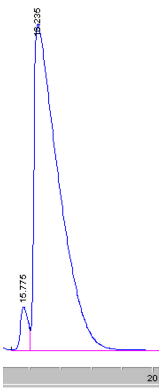

	Time	Area	Area%
Minor	15.775	4594.5	4.098
Major	16.235	107528.2	95.902
